# Prodrug-ML: prodrug-likeness prediction via machine learning on sampled negative decoys

**DOI:** 10.1007/s10822-025-00725-x

**Published:** 2026-01-10

**Authors:** Sadettin Y. Ugurlu, Shan He

**Affiliations:** 1 Novexus Ltd, 07058 Antalya, Turkey; 2 Department of Materials Science and Engineering, Faculty of Engineering, Akdeniz University, Dumlupinar Bulvari, 07058 Antalya, Turkey; 3 School of Computer Science, University of Birmingham, Edgbaston, Birmingham, B15 2TT UK

**Keywords:** Prodrugs, multimodel feature selection, sampling negative decoys, generating negative decoys

## Abstract

A prodrug is a pharmacologically inactive (or attenuated) derivative that undergoes bioreversible transformation in vivo to release an active parent drug, enabling temporary optimization of properties such as solubility, permeability, and targeting. Despite expanding catalogs of known prodrugs, *in silico* screening remains limited by the absence of reliable negative examples: training/evaluation sets often contain only positives or ad-hoc decoys, leading to class imbalance, property-mismatch shortcuts, and irreproducible benchmarks. Unfortunately, the limitation of reliable negatives has resulted in there being no efficient machine learning-based prodrug screening approach. Therefore, we introduce Prodrug-ML, an efficient *machine learning-based* screen for prodrug-likeness that prioritizes candidates rather than asserting mechanistic truth. *Prodrug-ML helps medicinal chemists* triage prodrugging ideas during hit-to-lead and lead optimization, filter enumerated libraries of promoiety–attachment variants before ADMET assays, and retrospectively mine internal/ChEMBL-like collections to surface likely prodrug chemotypes. *In practice*, users (i) generate or collect candidate structures (e.g., parent drug ± pro-moieties), (ii) score them with Prodrug-ML, and (iii) advance only high-scoring candidates to synthesis/assay, thereby reducing wet-lab load while maintaining chemical diversity. In order to achieve such practical usage, the Prodrug-ML framework, containing the default classifier, LightGBM, addresses these issues by (i) constructing three complementary, property-controlled negative cohorts (DUD-E–style near-misses, random ChEMBL, and strictly filtered ChEMBL), (ii) hardness control and label-noise guardrails on decoys, (iii) domain-bias control, and (iv) cross-decoy validation with multimodel feature selection. Produg-ML has been evaluated five times on hold-out data and an unseen test benchmark, after 80% of training data. In the benchmarks, the multimodel ensemble consistently improves early retrieval and overall discrimination, attaining $\textrm{EF}@1\%\approx 6\text {--}8$, $\textrm{EF}@5\%\approx 5\text {--}6$, $\textrm{BEDROC}_{20}\approx 0.78\text {--}0.82$, $\textrm{BEDROC}_{50}\approx 0.90\text {--}0.95$, and $\textrm{BEDROC}_{80}\approx 0.95\text {--}0.99$, alongside ROC AUC $\approx 0.86\text {--}0.87$, average precision $\approx 0.60\text {--}0.65$, and F1 $\approx 0.58\text {--}0.62$. As a result, these results, especially high BEDROC scores, are consistent with concentrating at least a prodrug within the top $\sim 2\text {--}3\%$ of ranked candidates, implying $\sim 97\text {--}98\%$ reductions in experimental time and cost when using standard wet-lab workflows that assay only the early tranche.

## Introduction

In 1958, Adrien Albert introduced the term “prodrug” framing a systematic design strategy for unmasking therapeutic activity within the body [1, 2]. A prodrug is a compound administered in an inactive or attenuated form that is converted *in vivo* into the active drug [3, 4]. The concept took shape in mid-20th-century medicinal chemistry [2, 5]. In contemporary terms, a prodrug is a deliberately engineered, bioreversible derivative of a parent drug that transiently modifies key developability attributes before controlled release of the active species [4, 6, 7]. Early precedents of prodrugs include prontosil, which is reductively transformed to sulfanilamide, and methenamine, which releases formaldehyde in acidic urine [8, 9]. Consequently, accumulated examples of prodrugs over the past couple of decades resulted in a systematic grouping to conduct deeper research on prodrugs.

Prodrug designs are conventionally grouped into two mechanistic classes: (i) carrier-linked derivatives and (ii) bioprecursors [10–13]. (i) In carrier-linked prodrugs, a temporary promoiety (e.g., esters, carbamates, carbonates, phosphates) is attached to mask polar or labile groups and improve absorption, stability, or distribution. *Activation* occurs when this bond is cleaved—usually by hydrolases (esterases, amidases, phosphatases) or by chemical hydrolysis—releasing the parent drug [11, 14, 15]. (ii) In bioprecursor designs, the drug scaffold itself is metabolically converted into the active species via oxidative, reductive, or eliminative steps, commonly mediated by enzymes such as cytochrome P450s, reductases, or microbiota-derived enzymes [10, 16–18]. Also, carrier-linked esters and phosphates are frequently deployed to enhance solubility and permeability for oral delivery, whereas bioprecursors are often exploited for tissue- or microenvironment-selective activation (e.g., colon-, hypoxia-, or microbiome-triggered release) [16, 17, 19, 20].

Besides group-based advantages, including enhancing solubility thanks to carrier-linked derivatives [19, 20] and microenvironment-selective activity with the help of bioprecursors [16, 17], prodrug strategies are effective because activation can be tuned to the biological context [7, 21]. Such flexibility yields several practical benefits: modulation of *in vivo* solubility [22–24] and permeability [25, 26]; higher oral bioavailability [24, 27] and metabolic stability (or, when desired, deliberate soft-drug behavior) [28]; reduced local toxicity and irritation [3, 29]; improved palatability [30, 31]; use of nutrient-mimetic transport pathways (e.g., peptide or amino-acid transporters) [32, 33]; and depot formation and long-acting delivery via lipophilic promoieties [34]. Local physicochemical conditions—pH, redox state, and ionic strength—often initiate or accelerate the key enzymatic or chemical steps (e.g., hydrolysis by esterases, amidases, or phosphatases; oxidative or reductive conversions), allowing control over the site, rate, and selectivity of release [14, 35, 36]. Because of these advantages, prodrugs have become standard tools for overcoming ADMET and delivery bottlenecks: they are considered early in lead optimization, evaluated alongside classical medicinal chemistry tactics, and supported by regulatory precedents and assay workflows that separately quantify prodrug, parent, and active exposures [7, 37, 38]. Consequently, these advantages have stimulated prodrug research and progressively expanded the available body of prodrug data.

The expansion of the available body of prodrug data results in curated repositories by collecting prodrug data. Curated repositories—most notably *smProdrugs: A Repository of Small-Molecule Prodrugs* [39]—aggregate more than 600 annotated prodrugs, including parent–prodrug pairs, activation routes, and ADMET/PK fields, creating a solid substrate for computation [39]. However, the absence of ground-truth negatives sharply limits machine learning–based prodrug screening. Specifically, supervised models require both positives and negatives to learn a robust decision boundary, calibrate probabilities, and set actionable thresholds [40, 41]. If only positives are available—or if negatives are assembled ad hoc—three issues follow: (i) severe class imbalance and label bias inflate apparent performance [42–45]; (ii) models exploit trivial bulk-property differences when decoys are not physicochemically matched (e.g., molecular weight, logP, ring count), yielding over-optimistic metrics that fail prospectively [46, 47]; and (iii) benchmarks become irreproducible across studies [48–50]. Therefore, standardized, property-matched decoy sets—together with explicit guardrails against label noise and domain bias—are prerequisites for fair evaluation and reliable “prodrug-likeness” scoring that can actually guide early triage, focus synthesis/ADMET resources, and improve prospective hit quality during prodrug screening. However, no open, efficient screen currently operationalizes these prerequisites for everyday medicinal chemistry use because of the “no-negative” bottleneck, which limits the build an effective, transparent classifier model. What is needed is a transparent classifier model that (i) learns from positives while sampling diverse, property-matched negatives, (ii) resists decoy “recipe” artifacts, and (iii) returns a calibrated score that teams can threshold according to project goals (precision vs. recall).

Here, we present Prodrug-ML: Prodrug-Likeness Prediction via Machine Learning on Sampled Negative Decoys. Prodrug-ML using LightGBM as the default classifier is positioned upstream of ADMET assays as an early machine-learning-based triage filter to retain highly positive prodrug structures. Specifically, typical scenarios include: (1) Lead optimization—screen enumerated promoiety variants of a known drug to prioritize a tractable subset for synthesis; (2) Library design—pre-filter virtual libraries to enrich for prodrug-like entries before docking/ADMET; (3) Retrospective analysis—scan legacy/internal collections to surface overlooked prodrug candidates for re-profiling. The model outputs a probability-like score; users set an operating threshold to balance yield vs. false positives according to project needs. To achieve such scenarios, the framework assembles three complementary negative cohorts—(i) DUD-E-style decoys, (ii) randomly sampled ChEMBL ligand compounds, and (iii) strictly filtered ChEMBL negative ligands constrained by medicinal chemistry rules and property windows. Using *three orthogonal decoy sources* efficiently samples the negative space while diluting source-specific artifacts, thereby reducing decoy bias and improving out-of-source transfer. After InChIKey de-duplication and parent–prodrug reconciliation, an untouched test set is created before any preprocessing or modeling to curb bias and leakage and to better approximate operational prodrug-screening conditions. Then, decoy quality is tightened via hardness control and label-noise guardrails (excluding near-duplicates of positives, removing trivially easy outliers, and suppressing source artifacts), followed by dataset composition controls that enforce realistic class ratios and balanced representation across decoy sources. A domain-bias-aware feature-selection screen then prunes features that act as recipe detectors; because this substantially limits per-view feature counts, a multimodel feature-selection strategy is applied to recover signal and improve stability [51, 52]. Robustness to negative “recipes” is stress-tested with cross-decoy validation and feature-set selection, retaining the combinations that transfer performance across sources. Final benchmarking on hold-out and unseen test set uses harmonized, stratified splits across multiple algorithmic families—Random Forest, ExtraTrees, k-Nearest Neighbors (k-NN), XGBoost, Bagging, and LightGBM—with evaluation centered on early recognition ($\textrm{EF}@1\%,@5\%$, BEDROC) and global metrics (ROC-AUC, Average Precision). Also, Y-randomization controls and external hold-outs are incorporated to guard against chance correlations. Consequently, the resulting pipeline directly addresses the negative-set bottleneck and supports a robust, data-driven prodrug-likeness score suitable for prospective screening. The codes, trained model, and Prodrug-ML framework are freely available for academic use: https://github.com/yauz3/prodrug-ml

## Materials and methods

**Fig. 1:**
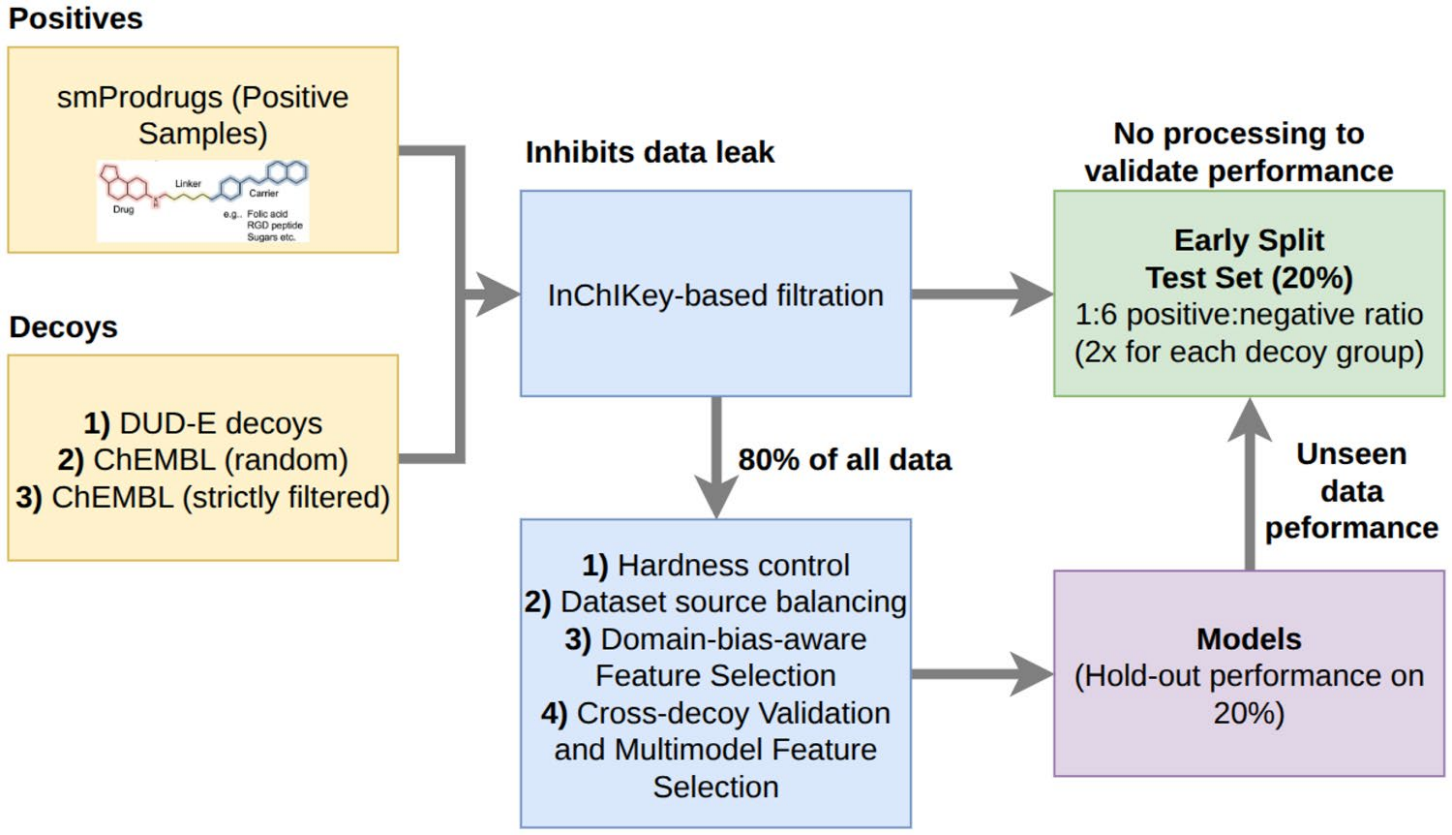
Workflow of the Prodrug-ML framework from preparation of decoys to validating on hold-out and unseen test data. Positive samples are collected from curated small-molecule prodrugs (smProdrugs), while negatives are drawn from three complementary decoy sources: (i) DUD-E property-matched decoys, (ii) randomly sampled ChEMBL compounds, and (iii) strictly filtered ChEMBL compounds. To prevent data leakage, InChIKey-based filtration is applied before any processing. An early-split test set (20% of all data) is carved out with a 1:6 positive-to-negative ratio, balanced across decoy groups (two negatives per source for each positive), and left completely untouched for final performance validation. The remaining 80% of the data undergoes hardness control, source balancing, domain-bias-aware feature selection, and cross-decoy validation with multimodel feature selection [51, 52]. Base models are then trained and benchmarked on this training portion, and the base models are linearly weighted (1:1) to build a final ensemble model architecture, with a LightGBM classifier as the default model. Finally, the final generalization is assessed exclusively on the unseen early-split test set for the base models and ensemble structures

**Fig. 2:**
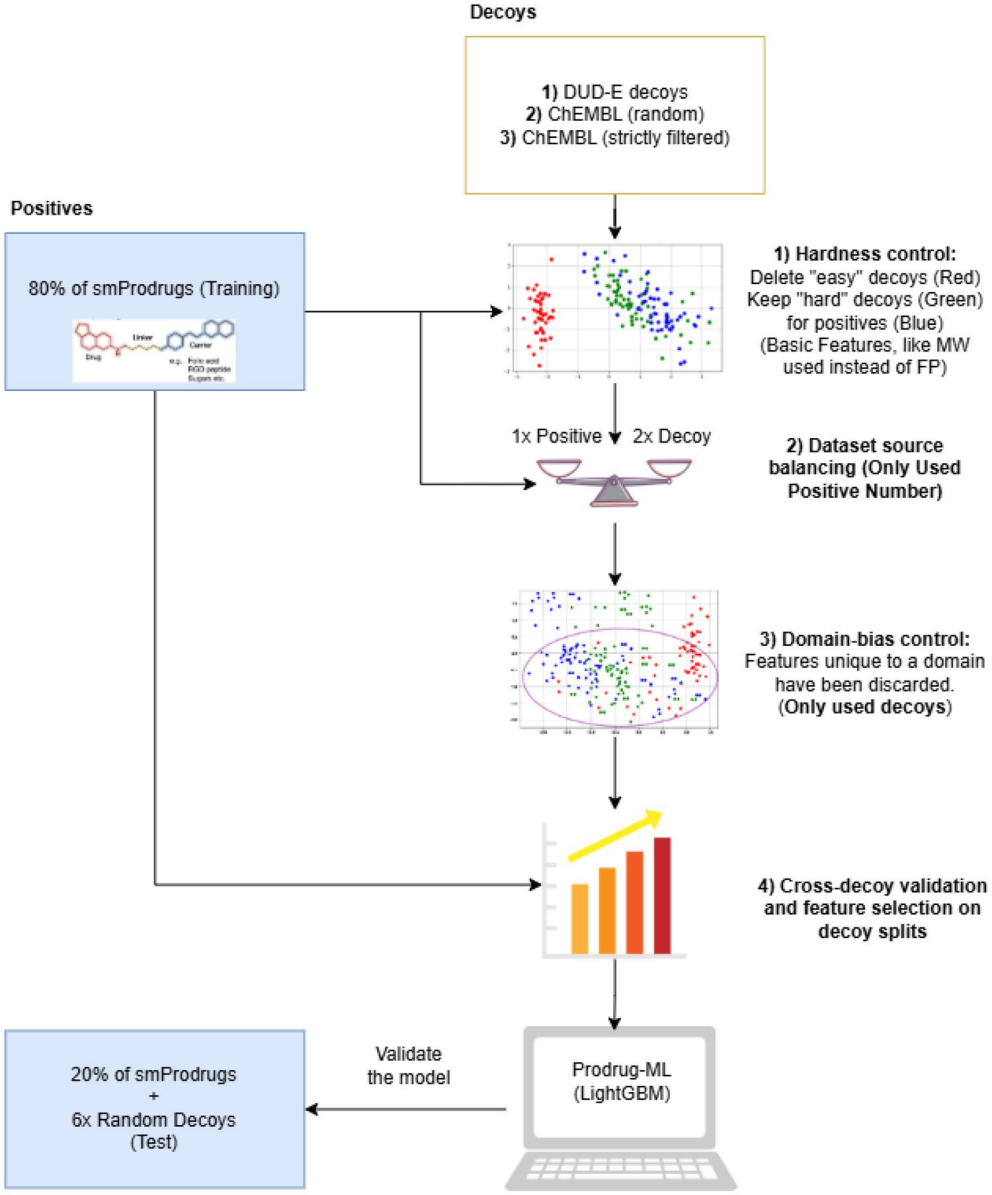
Schematic overview of the Prodrug-ML dataset construction and validation pipeline, showing the generation of property-matched decoys, hardness and domain-bias filtering, dataset balancing, and cross-decoy validation used for model training and evaluation. The figure outlines the building negative sample for Prodrug-ML. The left panel represents the positive set (experimentally verified prodrugs from smProdrugs), which is divided into 80% training and 20% testing subsets. The right panel shows the generation of three complementary negative cohorts (DUD-E, random ChEMBL, and strictly filtered ChEMBL). These decoys undergo successive filtering steps: (1) hardness control removes “easy” negatives based on simple physicochemical properties, (2) dataset source balancing equalizes class sizes according to the number of positives, and (3) domain-bias control discards features uniquely identifying dataset origin. The cleaned and balanced data ($1\times$ positive: $2\times$ decoy) are then used to train the LightGBM-based Prodrug-ML model, with performance validated using the reserved 20% test set and sixfold random-decoy augmentation without any preprocessing, such as hardness control, domain-bias control, and cross-validation

Prodrug discovery benefits from machine learning–based screening to conserve time and resources; however, existing prodrug repositories lack experimentally validated negatives. To address the lack of negative samples, which hinders the growth of prodrug studies and discovery, Prodrug-ML is introduced. The study of the Prodrug-ML framework by benefiting LightGBM as the default classifier is organized into three components: (i) *Building of Negative Samples*, covering data curation, principled construction of decoy/negative cohorts, overlap control, feature generation, and multimodel feature selection; (ii) *Model Training, Validation, and Testing*, detailing model development and evaluation together with the employed performance metrics; and (iii) *Analysis of Prodrug-ML fundamentals* that examines robustness, including label randomization, correlation heatmap, and hierarchical feature clustering to understand the mechanism of the Prodrug-ML (Figs. 1, and 2).

### Building of negative samples

Robust supervised learning requires high-quality negatives that do not trivially differ from positives and do not inadvertently leak label information. Accordingly, seven steps have been adopted to build a high-quality negative set (Fig. 2): (i) The Construction of Negative Decoy Sets; (ii) Overlap Control: InChIKey Matching; (iii) The Preparation of Fingerprint Features; (iv) Test-set Isolation: Early Split to Inhibit Bias; (v) Hardness Control and Label-noise Guardrails on Decoys; (vi) Dataset Source Balancing; (vii) Domain-bias-aware Feature Set Selection; and (viii) Cross-decoy Validation and Multimodel Feature Set Optimization.

#### The construction of negative decoy sets

Relying on a single negative source can yield deceptively high performance: decoys become easy to distinguish, selection bias increases, and domain-specific artifacts dominate. To mitigate these effects and to support cross-recipe generalization, three complementary decoy cohorts were assembled with fixed sizes and transparent sampling rules: DUD-E-style decoys at a 6:1 ratio per positive, random ChEMBL ($n=5000$), and strictly filtered ChEMBL ($n=5000$) (Fig. 2).

1. DUD-E-style decoys (“dude”): Property-matched decoys were generated with the public DUD-E workflow (https://dude.docking.org/generate), which returns $\approx 50$ purchasable ZINC compounds per input ligand (per protonation state) [53]. Matching is performed on core physicochemical axes-molecular weight, cLogP, hydrogen-bond donors/acceptors, rotatable bonds-and includes net formal charge. To minimize false decoys, candidates are forced to be topologically dissimilar to the query ligand (fingerprint-based filtering that retains only the most dissimilar fraction) [53]. Such a strategy yields “near-miss” molecules that mimic the bulk properties of prodrugs without sharing their chemotypes, producing challenging, bias-controlled negatives that prevent trivial separation by simple descriptors and support reproducible benchmarking of prodrug-likeness models [53] (Fig. 2).
2. Random ChEMBL (“chembl_random”, $n=5000$**):** Small molecules were sampled uniformly at random from ChEMBL via the REST API, restricted to entries of type “Small molecule” with defined structures (canonical SMILES/InChIKey available) [54]. No property windowing or target-based filtering was applied. Because ChEMBL aggregates compounds associated with both active and inactive bioactivity records, uniform sampling yields a chemically broad background that plausibly spans the spectrum from inactive to bioactive ligands. This cohort was therefore used to approximate the unlabeled library background against which prodrugs must be distinguished, helping to reduce covariate shift and to discourage trivial separability based on simple property ranges (Fig. 2).
3. Strictly filtered ChEMBL (“chembl_high_conf”, $n=5000$): To supply *high-confidence negatives*, a strictly filtered ChEMBL cohort was constructed as a complement to *chembl_random* [54]. Whereas *chembl_random* deliberately preserves broad background chemistry-including potential bioactives-to reflect real-library covariates, the filtered set prioritizes certainty over breadth by removing latent prodrug liabilities and assay-interfering/reactive motifs. Moreover, the two cohorts reduce negative-label noise while maintaining robustness against changes in background composition (Fig. 2).
     Construction began from a larger ChEMBL pool (“Small molecule” entries with defined structures), followed by RDKit/SMARTS filtering (Fig. 2): (a) element whitelist (C, H, N, O, S, P, F, Cl, Br, I, B, Si); (b) removal of labile *promoieties* and common bioactivation handles (e.g., esters, carbonates, carbamates, phosphate/phosphonate/sulfate esters; azo, nitro/nitroso, quinone-imine, catechol-like motifs); (c) exclusion of reactive/ligating warheads (e.g., aldehydes, acyl halides, anhydrides, isocyanates, epoxides/aziridines, Michael acceptors, vinyl sulfone/sulfonamide, chloroacetamide, sulfonyl chloride, imines/oximes, hydroxamic acids); (d) PAINS/Brenk (and NIH/ZINC, where available) alerts; and (e) drug-likeness windows approximating Lipinski/Veber (MW $\le 500$, HBD $\le 5$, HBA $\le 10$, $-1 \le \textrm{cLogP} \le 5$, TPSA $\le 140$, ROTB $\le 10$).

In summary, the three cohorts—property-matched near-misses (DUD-E), chemistry-agnostic background (random ChEMBL), and rule-hardened non-prodrugs (filtered ChEMBL)—offer complementary coverage that mitigates selection and domain bias while balancing *hardness*, *breadth*, and *label certainty* (Fig. 2). Specifically, DUD-E decoys frustrate trivial, property-based separability; random ChEMBL preserves real-library covariates by mixing potential actives and inactives; and filtered ChEMBL supplies conservative, low-noise negatives by excluding latent prodrug liabilities and assay-interfering motifs. In combination, this triad reduces overfitting to any single decoy recipe, enables robust cross-decoy validation under background shift, and provides a bias-aware foundation for training, early-enrichment assessment, and deployment to realistic screening libraries.

#### Overlap control: InChIKey matching

All structures were standardized in a single workflow and screened for cross-pool identity using the IUPAC InChIKey. The 14-character connectivity layer has been used to flag potential duplicates across the positive set (prodrugs and parents) and all decoy sources, then applied the full 27-character InChIKey to resolve stereochemical/tautomeric identity. Any molecule present in the positive set was removed from the decoy pools to prevent label leakage, and exact duplicates within a pool were collapsed to a single record. These steps ensure that every retained compound is unique with respect to both identity and dataset role; scaffold-aware splitting is handled downstream and described separately.

####  The preparation of fingerprint features

Binary substructure fingerprints were computed using the Avalon algorithm (RDKit, pyAvalonTools). Each molecule was represented as a 2,048-bit vector (Avalon_FP_*) that encodes hashed atom–bond environments and modular substructure keys. Unparsable SMILES were excluded after standardization to avoid sentinel patterns; all analyses operated only on valid Avalon fingerprints.

#### Test-set isolation: early split to inhibit bias

A test set must remain untouched by any data-dependent decisions to prevent optimistic bias and leakage. Early isolation of the test split ensures that similarity thresholds, representation choices, and domain-bias controls are derived solely from training data, so that reported generalization reflects performance on unseen chemistry. Therefore, from the curated positives ($$n_{P}=610$$), a stratified $$20\%$$ random sample defined the test–positive set $$\bigl (n_{P,\text {test}}\approx 122\bigr )$$ (Table 1). For each test positive, exactly two negatives were sampled from each decoy source—$$2\times $$dude, $$2\times $$chembl_random, and $$2\times $$chembl_high_conf—without replacement within source. Such a design enforces a per-positive 1 : 6 ratio and yields a source-balanced test mixture $$ ( \approx 2{\mkern 1mu} n_{{P,{\text{test}}}} \approx 244{\text{ negatives per source; }} $$$$ \approx 6{\mkern 1mu} n_{{P,{\text{test}}}} \approx 732{\text{ negatives in total}}) $$, thereby mimicking screening imbalance while preserving per-source parity. Such a 1:6 setting is a pre-registered, source-balanced compromise between (i) approximating early-screening class imbalance (few positives among many candidates), (ii) ensuring per-source parity to prevent decoy-recipe confounding, and (iii) controlling variance of early-recognition estimates with a fixed, integer-simple design (two negatives per source per positive). Importantly, the headline metrics emphasized in this work—ROC-AUC—are either prevalence-invariant (ROC-AUC) or explicitly normalized to the expected random early-ranking at the given prevalence (BEDROC), and EF is reported in its standard normalized form; accordingly, the 1:6 choice does not inflate apparent performance but stabilizes variance while preserving interpretability. Also, while Average Precision (AP) is prevalence-sensitive, keeping a fixed 1:6 prevalence across all test evaluations ensures fair comparability between models and decoy mixtures.

**Table 1:** Dataset composition under the early-split protocol: counts of positives and decoys in the training (80%) and fixed test (20%) partitions. A 1:6 positive-to-negative ratio is enforced by drawing exactly two negatives from each decoy source (DUD-E, ChEMBL_random, ChEMBL_high_conf) per positive, yielding source-balanced negatives in each split

Type	Training	Test
Positives	488	122
DUDE Decoys	976	244
ChEMBL random decoys	976	244
ChEMBL high conf. decoys	976	244
Total	**3416**	**854**

Counts reflect the planned split and sampling scheme: the test set contains 20% of positives (here 122) and, for each test positive, two negatives from each source (DUD-E, ChEMBL_random, ChEMBL_high_conf), giving 244 per source and 732 total negatives; the remaining 80% positives (488) follow the same 1:6 design for training (976 per source; 2928 total negatives). The per-positive “two-per-source” rule (i.e., 1:6 overall) yields a simple, reproducible integer design that equalizes source contributions per positive, simplifies stratified resampling/CI computation, and keeps the test distribution fixed—facilitating fair, prevalence-consistent comparisons across models and feature-set variants. Therefore, such construction mimics screening-class imbalance while equalizing decoy contributions and avoids leakage by isolating the test split before any data-dependent step

In summary, the early isolated test set preserves a fixed 1 : 6 positive:negative ratio with equal contribution from each decoy source and was used only once for final evaluation. Therefore, by preventing any test-time influence on thresholds, transforms, or feature masks, this protocol inhibits bias and yields an unbiased estimate of generalization to unseen chemistry. To operationalize this protocol without ever touching the test split during model or feature selection, we proceed as follows:

- Train-only preprocessing and audits. All preprocessing and selection steps—hardness control of decoys, domain-bias auditing, *k*-fold cross-validation, and per-view feature selection—are performed *exclusively within the training partition*. The isolated test set is not used for any threshold setting, model fitting, feature ranking, or hyperparameter adjustment, ensuring it remains an unbiased sample of chemical space.
- Nested training/hold-out evaluation plus one-shot test. After the train-only preprocessing, models are fit in a 5-fold scheme inside the training set (each fold trains on $80\%$ of the training data and evaluates on the remaining $20\%$ hold-out of the training data). Separately, the *same* trained models are evaluated once on the untouched test data. This yields paired performance traces on (i) processed, in-fold hold-outs and (ii) the independent, untouched test chemistry.
- Consistency across folds as evidence against memorization. When the performance on the five processed hold-out folds closely matches the performance on the untouched test split across EF, BEDROC, ROC AUC, and AP—with low variance across folds—this convergence indicates that the models are capturing transferrable prodrug-relevant structure rather than memorizing split- or recipe-specific artifacts. The agreement across all 5 folds and the final test reinforces Prodrug-ML’s validity for early prodrug screening.

Nevertheless, while the fixed 1 : 6 positive:negative ratio is a reasonable proxy for early screening imbalance and provides a controlled basis for comparison, real-world class balance will vary across projects and stages (e.g., focused libraries, hit-enriched cycles). In settings with a higher positive prevalence than 1 : 6, precision–recall behavior typically improves for a machine learning-based scorer like Prodrug-ML; conversely, more extreme imbalance can depress precision at fixed thresholds. More importantly, in such highly imbalanced regimes, rank-based early-retrieval metrics—EF@1%/EF@5% and BEDROC at multiple $\lambda$ (20, 50, 80)—provide more robust, threshold-insensitive summaries of screening utility than raw precision/recall at a fixed cutoff. Therefore, ROC, AP, F1, and EF@1%/EF@5% and BEDROC at multiple $\lambda$ (20, 50, 80) have been provided to validate Prodrug-ML performance on 1:6 imbalance benchmark as a reference point. Consequently, because Prodrug-ML outputs probability-like scores, users can retune operating points (thresholds, top-*k* selection) to their local prevalence, operation time, and cost structure.

#### Hardness control and label-noise guardrails on decoys

The goal of hardness control and label-noise guardrails on decoys is to prevent “too-easy” negatives from inflating apparent performance via memorization and to mitigate domain bias so that the classification task remains informative and useful in practice (Fig. 2). All hardness-control and guardrail steps (similarity-based trimming, source-predictability pruning, and cluster entropy filtering) were applied *exclusively within the training folds*. The external test split was left unchanged, ensuring an untouched, independently sampled evaluation set and preventing any leakage of evaluation information into training.

Curation operated entirely in the Avalon feature space: the full set of Avalon_FP_ columns was used both for similarity computations and as model features on only training data (Fig. 2). (1) A label-noise guardrail excluded near-duplicates of positives by applying an upper bound on each negative’s maximum Avalon-Tanimoto similarity to the positive pool (e.g., $>0.90$ excluded) and a lower bound (e.g., $<0.30$ excluded) to remove trivially dissimilar outliers; positives served only to compute these similarities and were applied uniformly in subsequent analyses. The upper bound prevents false negatives by removing near-duplicates of positives from the decoy pool, thereby avoiding mislabeled negatives. On the other hand, the lower bound addresses a different failure mode: even after property matching, some decoys can lie far outside the positives’ chemical neighborhood and become trivially separable, which inflates EF/BEDROC and exaggerates apparent generalization without improving task learning. Excluding only the most distant tail (here, $\textrm{max}\_\textrm{sim}<0.30$ to any positive) constrains negatives to a broad but relevant applicability domain while preserving diversity; it is verified that scaffold counts, similarity distributions, and low-dimensional embeddings of the retained negatives remain well dispersed relative to the positives. Therefore, bounds were derived from the empirical distribution of the training positives’ maximum Avalon–Tanimoto similarity to the training positive pool. (2) To suppress source artifacts, a domain classifier (ExtraTrees, out-of-fold probabilities) was trained on **negatives** to predict their source; candidates with high maximum predicted source probability were pruned, favoring negatives whose origin is difficult to infer. (3) Negatives were embedded via PCA of the Avalon bit vectors and clustered with *k*-means; clusters with low source entropy (dominated by a single “recipe”) or with low mean similarity to positives were removed, filtering recipe pockets and obvious outliers (Fig. 2). Consequently, the retained decoys occupy an intermediate similarity regime. They are difficult to attribute to any specific source, thereby reducing label noise and domain bias while preserving task difficulty and practical utility.

####  Dataset source balancing

Screening scenarios are heavily imbalanced toward non-prodrugs; a controlled class ratio and per-source balancing were imposed to reflect this setting while limiting domain overrepresentation (Fig. 2). Therefore, selection was capped at a positive:negative ratio of 1 : 6 and balanced across three decoy domains (approximately two negatives per domain per positive), with random sampling within each domain after filtering to maintain balance. As a result, the final set comprises 488 positives and 976 negatives: 976 DUD-E decoys, 976 ChEMBL random decoys, and 976 ChEMBL filtered decoys (Table 1).

#### Domain-bias-aware feature set selection

Features that reveal the source of a decoy (e.g., DUD-E, ChEMBL (random), ChEMBL (high_conf)) can produce a deceptive boost in performance by enabling recipe recognition rather than prodrug recognition. The aim is to reduce such recipe-specific artifacts while preserving signal relevant to the primary task (Fig. 2). Therefore, a multi-class domain classifier (ExtraTrees) was trained only on negatives in the Avalon fingerprint space (Avalon_FP_). Random subsets of these features were evaluated under cross-validation, and subsets that pushed domain prediction toward chance were preferred, implicitly penalizing features with strong source cues. Positives were *not* used to train the domain classifier or to choose the subset. A light compatibility check then verified that the selected subset preserved at least $95\%$ of the baseline ROC AUC on a small, fixed validation split for the primary task (prodrug vs. non-prodrug); any subset failing this check was discarded.

In order to maximize the number of usable features without introducing domain-based leakage, more than 30 different feature sets were assembled. To ensure that no spurious domain signal was inflating performance, we applied relaxed thresholds on auxiliary “domain classifiers”: specifically, a mean accuracy below 0.4, balanced accuracy below 0.4, macro-F1 below 0.4, and macro-AUC-OVR below 0.6. These thresholds were chosen relative to chance-level performance in a three-class setup (random expectation $\approx 0.333$), allowing for a small buffer above random while still ruling out any significant predictive power from domain labels. In other words, the feature sets retained under these criteria showed no considerable domain-based leakage, thereby reducing the risk of deceptively optimistic results. Therefore, adversarial screening against the domain classifier reduced source predictability toward chance while maintaining primary-task performance, yielding a representation that is minimally confounded by recipe artifacts yet retains prodrug-relevant information.

#### Cross-decoy validation and multimodel feature set optimization

Cross-decoy evaluation tests whether decision boundaries depend on a particular negative “recipe” (Fig. 2). If a model trained with one decoy source transfers its performance to other sources, the learned signal is less likely to reflect recipe artifacts and more likely to capture prodrug-relevant structure. Therefore, cross-decoy validation guards against spurious gains driven by decoy-source artifacts. Cross-decoy validation operated in the Avalon fingerprint feature sets (Avalon_FP_; which have been selected in the Domain-bias section. Using a fixed split of positives (80 : 20 for train:test), a baseline classifier (ExtraTrees, class-weighted, median imputation) was trained on *positives from the training split* plus *negatives from exactly one decoy source* (e.g., dude). Five-fold stratified CV on this mixture provided a within-recipe reference. The same model, refit on the full training mixture (pos_train + one decoy source), was then evaluated on *positives from the test split* combined with *negatives from each alternate decoy source* (e.g., chembl_random, chembl_high_conf). In the evaluation, primary metrics were ROC AUC and average precision. Consequently, feature sets that exhibited strong performance on one decoy source but only moderate or poor transfer to the others were discarded, while those 30 feature sets demonstrating consistent cross-decoy performance were retained for multimodel feature selection.

As for multimodel feature selection, to enhance the robustness of the final model, feature sets were deliberately constrained in size to mitigate the risk of domain bias. Instead of merging or artificially expanding these sets, we implemented a random search strategy across 30 candidate feature groups, each assessed using a domain-based hold-out. Individual models were trained on distinct feature sets, and the combinations achieving the highest mean ROC AUC values were retained. For instance, feature groups such as list_7 and list_12 were employed to train separate classifiers, after which their outputs were integrated through late fusion by linearly weighting (1:1) the model predictions. This ensemble strategy effectively attenuated domain-specific artifacts while maintaining high predictive accuracy and stability. In summary, cross-validation combined with multimodel feature selection thus ensured the elimination of decoy-driven bias, validating Prodrug-ML’s performance as both robust and practically applicable.

### Model training, validation and testing

Publicly accessible prodrug resources remain limited, whereas *smProdrugs*—published in 2023—curates the largest and most up-to-date collection with $\sim$600+ prodrugs and enables ongoing community submissions. To reduce positive-class heterogeneity and selection bias across disparate sources—and to keep the methodological focus on the principled construction of *negative* cohorts—the positive class was drawn exclusively from smProdrugs [39]. Although the absolute number of positives is modest by modern machine-learning standards, it represents the most comprehensive and actively maintained corpus available. Accordingly, the study design prioritized robustness under limited-data conditions: an early, stratified 80:20 train–test split with a frozen test set, feature selection restricted strictly to the training pool, and ensemble modeling choices aimed at transparent and reproducible evaluation.

The most updated dataset [39] was first *early split* into a stratified $80\%{:}20\%$ train–test partition; the $20\%$ test split remained untouched for any data–dependent choice (Fig. 1). All training, model selection, and feature decisions were confined to the $80\%$ pool. Within this pool, two complementary protocols were applied and, in each case, performance was also recorded on the fixed $20\%$ test split for descriptive stability (test results were not used for tuning or selection) (Table 1).

Models were trained under a standardized pipeline on structure-standardized molecules using predefined fingerprint feature lists (Fig. 1). Although binary inputs do not inherently contain missing values, a SimpleImputer (median) was retained as a robustness safeguard (operating as a no-op under normal conditions). The scaling to [0, 1] was included only for base learners that require it, with scalers fitted strictly within cross-validated training folds. Finally, base models have been linearly weighted (1:1) to construct the ensemble structure.

As for the ensemble structure, feature lists were first identified via Domain-bias-aware Feature Set Selection, and candidate combinations of lists were evaluated in Cross-decoy Validation and Feature Set Optimization. To assess a multimodel signal without mixing classifier families, homogeneous ensembles were constructed by training the *same* base classifier separately on multiple feature views and summing to one linear combination (1:1) of their scores to build a multimodel ensemble feature selection framework [51, 52]. As a result of multimodel ensemble feature selection, diversity thus arises solely from the feature view; also, naïve pre-merging of views into a single feature set (i.e., concatenation akin to “multimodel ensemble feature selection”) was avoided due to its propensity to inflate apparent performance under domain/decoy shift [51, 52]. Consequently, the selected single-view list and late fusion feature lists (multimodel configuration) were used to train base models and construct ensemble frameworks by linearly weighting the predictions of base models.

Five-fold hold-out and test performance: The $80\%$ pool was partitioned into five label-stratified folds. In each iteration, a model was fit on 4/5 of this pool ($\approx 64\%$ of all data) and validated on the remaining 1/5 ($\approx 16\%$). For each such model, predictions were additionally generated on the untouched $20\%$ early test split to quantify variability of test-set performance across training resamples (Fig. 1). After cross-validation, a final model was refit on the full $80\%$ pool and evaluated once on the early test split.

#### Performance metrics

The evaluation of machine learning models in virtual screening requires metrics that capture both early recognition ability and global classification performance, since the latter is critical in prioritizing a small subset of compounds from large libraries.

Enrichment factors (EF@1%, EF@5%). The enrichment factor quantifies how many more actives are found within the top-ranked fraction of a screened library compared with random expectation. EF@1% and EF@5% measure retrieval at the top 1% and 5% of the ranked list, respectively, thereby emphasizing early discovery of true positives under realistic screening conditions.

Higher EF is better, and widely used EF classes define practical meaning: $\textrm{EF}<2$ = depletion/minimal; $2 \le \textrm{EF}<5$ = moderate; $5 \le \textrm{EF}<20$ = significant; $20 \le \textrm{EF}<40$ = very high; $\textrm{EF} \ge 40$ = extremely high [55–60]. On this scale, EF@5% $\ge 5$ already denotes significant enrichment, making it a conservative acceptance bar for early retrieval at 5%. Likewise, EF@1% $\ge 5$ indicates significant enrichment, with higher values moving toward very high and extremely high classes [55–60]

Boltzmann-enhanced discrimination of ROC (BEDROC): BEDROC ($\lambda = 20, 50, 80$) introduces an exponential weight to prioritize hits recovered early in the ranking [61–63]. Different values of $\lambda$ determine the degree of emphasis on the top of the list: higher $\lambda$ values increase the penalty for late recognition. BEDROC thus complements EF by providing a continuous, normalized measure of early enrichment.

BEDROC lies in [0, 1] and increases as actives move earlier in the ranking; unlike EF, it exponentially weights *all* ranks with rate $\lambda$. There are no universally accepted thresholds; instead, $\lambda$ serves as a tuning parameter that controls the emphasis placed on the extreme top of the ranking. A useful rule of thumb is that about $80\%$ of the BEDROC weight comes from the first $f \approx \ln (5)/\lambda$ fraction of the list; hence $\lambda {=}20 \Rightarrow f{\approx }8\%$, $\lambda {=}50 \Rightarrow f{\approx }3.2\%$, and $\lambda {=}80 \Rightarrow f{\approx }2\%$.

Examples:

- If $\textrm{BEDROC}_{20}=0.80$, the ranking shows significant early recognition concentrated mostly within the top $\sim 8\%$.
- If $\textrm{BEDROC}_{50}=0.80$, the same high score is achieved under a stricter focus (top $\sim 3.2\%$), indicating even tighter clustering of actives near the top.
- If $\textrm{BEDROC}_{80}=0.80$, reaching 0.80 with emphasis on only the top $\sim 2\%$ implies extremely front-loaded recognition.

Average precision (AP): AP summarizes the precision–recall trade-off by averaging precision across all recall levels. It is particularly suitable for imbalanced datasets, where precision–recall curves better reflect model performance than ROC curves.

Area under the ROC curve (ROC AUC): ROC AUC measures the ability to distinguish actives from inactives independently of threshold choice, serving as a global indicator of discriminative capacity.

F1 score: The F1 score, defined as the harmonic mean of precision and recall, provides a balanced measure of classification effectiveness when both false positives and false negatives are relevant.

In summary, these metrics provide complementary perspectives: EF and BEDROC quantify early-recognition performance that is decisive in practical screening, whereas ROC AUC and AP characterize global discriminative quality, and F1 summarizes the precision–recall balance at a given operating point. Fortunately, a fixed 1 : 6 positive:negative benchmark is adopted to emulate early-stage screening imbalance and enable like-for-like comparisons. Under heavy imbalance, rank-based, threshold-insensitive measures—EF@1%/EF@5% and BEDROC at multiple $\lambda$—are the most robust indicators of early-retrieval utility; ROC AUC is likewise robust to class prevalence and threshold choice, reflecting overall ranking quality. By contrast, AP and F1 depend on prevalence and the selected threshold, and are therefore reported alongside the robust metrics to illuminate operational precision–recall trade-offs. In combination, such a panel reduces the bias of any single metric and ensures that both global discrimination and early enrichment are rigorously assessed.

#### Comparison analysis

As Prodrug-ML with the default classifier, LightGBM, represents the pioneering framework for prodrug-likeness modeling, no alternative benchmark exists for a complete comparative analysis against state-of-the-art methods. Therefore, in total, **15** fingerprint-based classifiers were implemented, spanning linear, kernel, probabilistic, tree/ensemble, neural, and meta-learning families: Logistic Regression (L2), Elastic-Net Logistic Regression, SVM (RBF), k-NN, Gaussian Naïve Bayes, Decision Tree, Random Forest, ExtraTrees (ET), Gradient Boosting, AdaBoost, Bagging, MLP, soft Voting, Stacking, and LightGBM. The variety of model performances ensures that global performance is achieved without model-selection bias.

Among the 15 classifiers, ET has been employed primarily as a *domain classifier* to identify and up-weight *hard negatives*—i.e., decoy compounds that are difficult to distinguish from positives—while down-weighting trivially separable decoys. Concretely, ET is trained within the 80% training pool (stratified 5-fold CV) to discriminate positives vs. each negative cohort; out-of-fold ET probabilities then guide hard-negative sampling for subsequent model training. Such a procedure reduces decoy-source “recipe” artifacts and enforces a more challenging decision boundary. Acknowledging the trade-off, hard-negative induction based on ET performance can modestly reduce absolute EF on the untouched test in exchange for cross-decoy robustness. Consequently, EF is reported to show that Prodrug-ML’s early-retrieval performance stems from the ET-guided, robustness-oriented training regimen rather than from EF-specific optimization.

As for clarity in the main text, three representative models were highlighted—k-NN, LightGBM (default classifier in Prodrug-ML framework), and Bagging—as they cover distinct inductive biases (instance-based, gradient-boosted trees, and variance-reducing bootstrap ensembles). Inclusion of k-NN is deliberate as a contrast baseline for similarity-based inference. In parallel, LightGBM and Bagging are non-similarity, tree-based learners—LightGBM captures non-linear, combinatorial interactions across fingerprint bits, and Bagging reduces variance via bootstrap aggregation of high-variance base learners. This triad (instance-based vs. interaction-focused vs. variance-reduced trees) enables a fair comparison under an identical pipeline, and the results consistently favor non-similarity approaches. Also, all 15 models were trained under the same preprocessing and evaluation protocol (median imputation, min–max scaling, stratified 5-fold validation on the training pool, and testing on the early-isolated split) and were evaluated with both global and early-recognition metrics (ROC AUC, AP, F1; EF@1%, EF@5%, BEDROC with $\lambda \in \{20,50,80\}$). Consequently, comprehensive results for the remaining 12 models (per-fold, out-of-fold/test bootstrap CIs, and early-enrichment statistics) are provided in the Supplementary Information to enable full reproducibility and independent comparison.

### Analysis of Prodrug-ML fundamentals

In order to understand performance improvement reasons and feature-binding site relationships, several analytical methods are employed to assess feature importance and relationships:

- Label randomness analysis: Evaluates the extent to which predictive performance exceeds that of randomized labels, thereby establishing a baseline and guarding against spurious correlations.
- Dimensionality reduction with PCA: Principal Component Analysis (PCA) linearly projects high-dimensional features into orthogonal components, enabling visualization of dominant variance directions and identification of potential redundancy.
- Non-linear feature projection with t-SNE: t-distributed Stochastic Neighbor Embedding (t-SNE) provides a non-linear projection of high-dimensional data into two or three dimensions, facilitating the exploration of class separability and latent structure.
- Feature Importance via ANOVA F-test: The ANOVA F-test quantifies the statistical significance of individual features by comparing variance across groups or classes, supporting the selection of features with high discriminative power.
- Model interpretability with SHAP: SHAP (SHapley Additive exPlanations) attributes a model’s prediction to individual features using game-theoretic Shapley values, yielding additive, signed contributions per feature for each molecule. Identifies which fingerprint bits/descriptors most strongly push predictions toward “prodrug-like” or “non-prodrug,” clarifies directionality (feature increases that help/hurt), supports cohort-robust importance ranking, and enables molecule-level rationalization to guide chemical hypothesis generation (e.g., which substructure patterns are repeatedly associated with prodrug-like scores).
- Correlation heatmap: A heatmap visualization of pairwise feature correlations highlights redundant variables, multicollinearity, and potential clusters of related descriptors. (Supplementary Information)
- Hierarchical feature clustering (dendrogram): Groups features according to correlation similarity and presents relationships in a hierarchical tree, enabling identification of redundant subsets or complementary clusters of descriptors. (Supplementary Information)
- Multivariate feature visualization with radViz: Radial visualization (RadViz) projects multivariate data points into a two-dimensional circle, where features are placed as anchors on the circumference. This facilitates intuitive inspection of feature contributions and class separability. (Supplementary Information)

These analyses collectively provide a deeper understanding of the feature space underlying prodrug-likeness prediction. By examining label robustness, reducing dimensionality, probing non-linear structures, and identifying feature relevance and redundancy, the methods clarify why Prodrug-ML achieves strong predictive performance. Beyond model validation, they also illuminate key descriptors and structural patterns associated with prodrug characterization, thereby offering guidance for refining feature engineering strategies and informing future studies on rational prodrug design.

### Summary of Prodrug-ML development and intended deployment

The Prodrug-ML framework utilizes the most comprehensive openly curated small-molecule prodrug dataset currently available (released in 2023), which aggregates parent–prodrug relationships and basic ADMET/PK annotations [39]. Despite such a resource, the field faces methodological gaps that hinder reliable *in silico* screening: (i) the absence of experimentally verified negatives, (ii) the lack of models that estimate activation mechanisms, conversion ratios/rates, or species-specific biotransformation, (iii) limited attention to applicability domain and uncertainty, and (iv) the absence of a standardized, calibrated prodrug-likeness score suitable for early triage of large libraries while preserving chemical diversity.

To address the “no-negatives” problem, Prodrug-ML is a machine-learning–based, early-stage screen designed to prioritize candidate prodrug designs before synthesis and ADMET assays. The input is a set of standardized SMILES strings (enumerated or hand-designed). The output is a calibrated, probability-like prodrug-likeness score (binary labels used for training; soft scores used for ranking) that supports threshold- or top-*k*–based selection according to program goals (precision- vs. recall-oriented operation). Also, Prodrug-ML operationalizes negative sampling through three complementary, property-controlled cohorts that broaden the non-prodrug space while suppressing recipe-specific artifacts: (i) DUD-E–style near-miss decoys to enforce physicochemical matching; (ii) randomly sampled ChEMBL small molecules to approximate real-library background; and (iii) strictly filtered ChEMBL negatives constrained by medicinal-chemistry rules and property windows to reduce latent prodrug liabilities and assay-interfering motifs. An early, source-balanced test split is isolated before any data-dependent step; InChIKey-based overlap control removes identity leakage; hardness control and label-noise guardrails prevent trivial separability; and domain-bias–aware feature screening suppresses features acting as decoy-recipe detectors. Cross-decoy validation favors feature sets and classifiers that transfer across negative sources, while homogeneous late-fusion ensembling over complementary feature views stabilizes ranking without concatenating features into a leakage-prone aggregate. Together, these steps yield a calibrated score that can eliminate low-priority structures in early triage while maintaining chemical breadth by overcoming the no-negative problem.

Typical use of Prodrug-ML cases includes: (1) Lead optimization—screen enumerated promoiety variants to prioritize a tractable synthesis list; (2) Library design—pre-filter virtual libraries to enrich for prodrug-like entries prior to docking/ADMET assays; and (3) Retrospective mining—rank legacy/internal collections to surface overlooked prodrug candidates. Operating points are selected via validation curves: higher thresholds for precision-oriented shortlists, lower thresholds for recall-oriented library curation, or top-*k* ranking for review lists.

In summary, Prodrug-ML, the first step of the long-term development for prodrug screening, provides five contributions to the prodrug research area. (i) Prodrug-ML demonstrates a reproducible route to address the “no-negatives” bottleneck in prodrug screening by combining three orthogonal decoy cohorts with cross-decoy validation. (ii) The calibrated score and operating guidance are practically useful for early screening, enabling list-size control and focused deployment of synthesis and ADMET resources. (iii) Component analyses (feature-set curation, correlation structure, dimensionality reductions, and cross-recipe transfer) illuminate prodrug-relevant patterns and provide actionable diagnostics for future mechanism-aware modeling; additional advantages include bias-aware dataset construction, early isolated testing, reproducible pipelines, and deployable threshold guidance. (iv) The domain-bias–aware feature-selection pipeline surfaces stable, cross-decoy substructure signals that align with known prodrug design motifs (e.g., ester/phosphate masking handles and attachment-site contexts), yielding chemotype-level hypotheses from hashed fingerprints and thus bridging from black-box ranking toward mechanism-aware interpretation and future SMARTS/cleavage-aware descriptors. (v) Most importantly, as one of the first openly documented machine-learning frameworks for structure-based prodrug triage following the 2023 dataset release, Prodrug-ML furnishes a reference point for subsequent studies; even with acknowledged limitations, it establishes a transparent baseline to accelerate future, mechanism-informed extensions.

## Results and discussion

Prodrug-ML using LightGBM as the default classifier is an end-to-end prodrug-likeness framework that curates complementary negative sets, guards against hardness and domain bias with cross-decoy checks, and linearly ensembles models trained on distinct feature views to build multimodel feature selection; and finally, evaluation stresses early enrichment alongside global discrimination. To validate and test Prodrug-ML, five sections have been designed. (i) Comparative Analysis, used to show how multimodel feature selection and linear weighting improve performance over single-view baselines under matched splits. (ii) Validation of the Dataset, representing domain-bias and cross-decoy validation. (iii) Analysis of Prodrug-ML—Factors Behind the Improvement: the sources of gain via targeted diagnostics (e.g., feature importance, and feature correlation) to confirm robustness rather than artifact. (iv) Case Study: Classifier Choice, Fragment-Level Signals, and Prodrug-Oriented Chemical Suggestions: the example of how the Prodrug-ML framework can be useful on unseen data. Finally, (v) Challenges and Future Opportunities, remaining limitations in data curation and representation, and sketch directions—richer features and prospective validation—to further strengthen Prodrug-ML.

### Comparative analysis

The comparative analysis centers on three representative families—Bagging, k-Nearest Neighbors (k-NN), and LightGBM (the default classifier in Prodrug-ML)—with results for an additional 13 models provided in the Supplementary Information. k-NN is included *deliberately as a contrast baseline*, acknowledging its mechanistic limitations for prodrug design where discrete, bioreversible masks (e.g., phosphate esters) can flip class labels with minimal scaffold change and are not well captured by distance-based similarity. The goal is not to endorse k-NN for this task but to demonstrate that the study’s conclusions are *not model-contingent*: performance patterns and early-enrichment gains persist across conceptually distinct learners. Also, beside the base model performance of three classifier, the multimodel ensemble for them under matched splits, focusing on two complementary aspects: (i) *early recognition*, where success is defined by how strongly true prodrugs are concentrated at the extremely top of the ranked list (quantified by EF@1%, EF@5%, and $\textrm{BEDROC}_\lambda$), and (ii) *global/thresholded discrimination*, where overall ranking quality and operating performance are captured by ROC AUC, Average Precision (AP), and F1. The first part (EF and BEDROC) supports practical list-sizing and early-batch planning; the second part (ROC AUC, AP, F1) assesses broader separation and default-threshold behavior.

#### Early enrichment: EF@1% and EF@5%

**Fig. 3:**
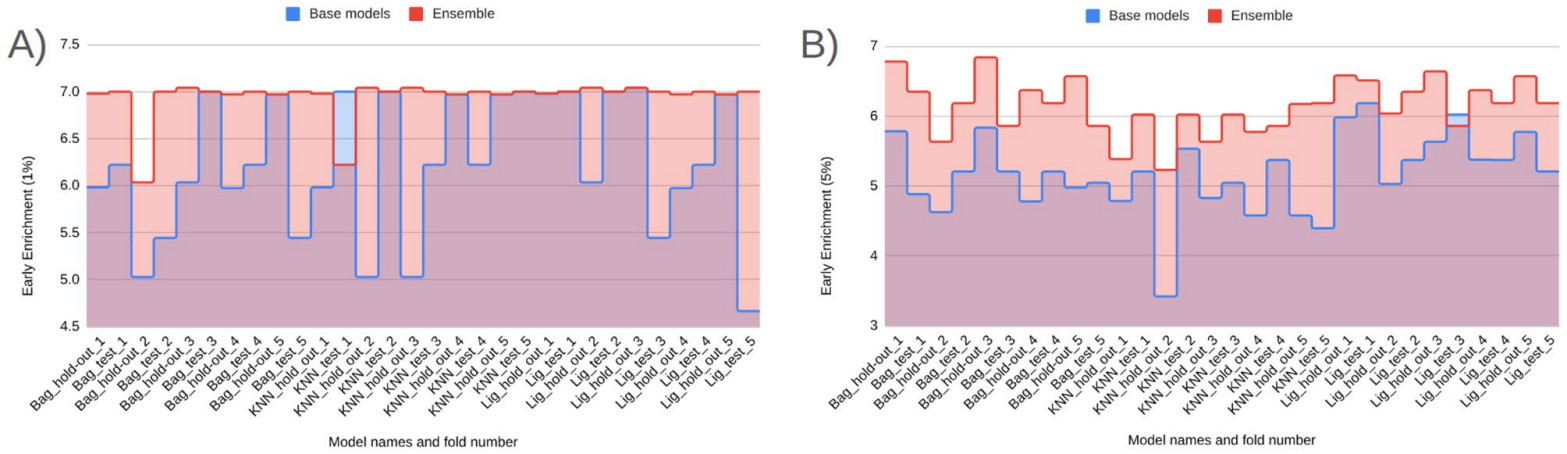
Performance of three model families (Bagging, k-NN, and LightGBM; default classifier), comparing base models (blue) and the equal-weight ensemble (red) for early enrichment at 1% and 5% across all folds. The figure contains two panels: (A) reports EF@1%, and (B) reports EF@5%. The x-axis lists the model family and split/fold identifiers (e.g., Bag_hold-out_1–5 and Bag_test_1–5, followed by k-NN_* and LightGBM_*), and the y-axis shows the early enrichment (EF) value. In each panel, the blue trace summarizes the base-model EF per fold, the red trace shows the corresponding ensemble EF, and the purple overlap indicates agreement between the two. Ensembles are computed by a 1:1 (equal-weight) linear average of base-model outputs, intentionally avoiding additional meta-parameters to keep the focus on addressing the “no-negative” limitation

Early enrichment at the top of the ranked list is critical for virtual screening because it measures how many true actives appear among the very first compounds a decision-maker would test. Accordingly, it has been reported that both EF@1% and EF@5% capture ultra-early and early retrieval quality, respectively. The comparative performance of the three model families—Bagging, k-NN, and LightGBM (default classifier)—on these two metrics across all folds is summarized in Fig. 3 (Panel A: EF@1%; Panel B: EF@5%).

From the base models (blue) (Fig. 3), visual averages indicate that LightGBM (default classifier) attains the strongest early enrichment overall (EF@1% typically in the high 5 s to near 7; EF@5% around mid 5 s to mid 6 s). Also, Bagging follows closely (EF@1% roughly mid 5 s to low 6 s; EF@5% about high 4 s to mid 5 s), while k-NN lags and exhibits larger variance (notable dips near  5 for EF@1% and down to  4 for EF@5%). Fortunately, most of the model’s performance on the hold-out and unseen early split test set is higher than 5 for EF@1%. Fortunately, such higher than 5 forEF@1% indicates a significant early detection performance in prodrug screening (Fig. 3) [55–60]. Although three models provide significant early detection in prodrug screening, across families, hold-out and test folds show consistent ordering, with LightGBM > Bagging > k-NN on average, and k-NN contributing the most fold-to-fold fluctuation. Beyond these three selected classifiers, the broader model battery (Supplementary Information) also achieves *significant* early recognition. On EF@1%, eight learners exceed the practical threshold of 5, including XGBoost ($\bar{E}F@1\%\approx 6.17$), CatBoost ($\approx 6.13$), ExtraTrees ($\approx 6.01$), Random Forest ($\approx 6.01$), Bagging ($\approx 6.03$), Voting ($\approx 5.91$), MLP ($\approx 5.92$), and SVM (RBF) ($\approx 5.20$). Importantly, this signal persists when the enrichment window is widened: EF@5% means remain at or near the desirable 5–6 range for several tree/ensemble and neural baselines—CatBoost ($\bar{E}F@5\%\approx 5.30$), ExtraTrees ($\approx 5.21$), XGBoost ($\approx 5.19$), Bagging ($\approx 5.16$), MLP ($\approx 5.08$), and Random Forest ($\approx 5.01$); the simple Voting scheme is competitive ($\approx 4.80$) (Supplementary Information). The presence of multiple, conceptually distinct learners performing at this level supports the absence of model-selection bias and indicates that early enrichment is *not* tied to a single algorithmic family. As expected, the use of harder, property-matched decoys can moderate enrichment on the unseen test split; this is an intentional stress test to prioritize generalization. Taken together, these results—at both EF@1% and EF@5%—indicate that the Prodrug-ML framework provides robust early detection behavior for prodrug screening.

Comparing base (blue) to ensemble (red) (Fig. 3), the red traces sit consistently at or above the blue across nearly all folds and in both the hold-out and the untouched early-split test, with a visibly larger margin at EF@5% (Panel B) than at EF@1% (Panel A). In the Fig. 3, when the bases dip to approximately $\sim 5$ at EF@1% and $\sim 4{-}4.5$ at EF@5%, the ensembles remain near or above $\ge 6$ and $\ge 5$, respectively—an uplift on the order of $+\!0.5$ to $+\!1.0$ EF points that also smooths the worst-case blue dips (Fig. 3). Although the base models already achieve significant early enrichment (EF$\ge 5$) in many folds, the multimodel feature-selection and ensembling further raise the early-enrichment plateau and reduce variance, yielding more reliable retrieval of actives in the top 1–5% of the ranked list. Taken together, these ensemble EF gains indicate that Prodrug-ML offers meaningful, operationally relevant improvement and has strong potential for prodrug screening with the help of multimodel feature selection.

As for the interpretation of the EF results, enrichment factors translate directly into expected hit counts in the shortlists that practitioners actually synthesize or assay. If a library of size *N* contains a fraction *p* of true prodrugs (unknown in deployment but estimable from prior campaigns), the random expectation in the top fraction *f* is $N\!\times \!f\!\times \!p$; with $\textrm{EF}@f$, the expected true prodrugs retrieved is $\textrm{EF}@f \times N\!\times \!f\!\times \!p$. Using the observed ranges (EF@1% $\approx 6$–8; EF@5% $\approx 5$–6), three common scenarios are illustrative: (i) *Lead optimization* (enumerations, $N{=}300$, assume $p{=}10\%$): screening the top $1\%$ ($f{=}0.01$, $\approx 3$ designs) at EF@1%$\,{=}\,7$ yields $\approx 7 \times 300 \times 0.01 \times 0.10 \approx 2.1$ true prodrugs in just three syntheses, i.e., a $\sim 7\times$ uplift over random and a tractable shortlist for rapid SAR; (ii) *Library design* (virtual library, $N{=}20{,}000$, assume $p{=}1\%$): testing only the top $5\%$ ($f{=}0.05$, 1, 000 compounds) at EF@5%$\,{=}\,5.5$ returns $\approx 5.5 \times 20{,}000 \times 0.05 \times 0.01 \approx 55$ true prodrugs versus $\approx 10$ at random, concentrating effort $20\times$ while recovering $\sim 5.5\times$ more actives within the same budgeted panel; (iii) *Retrospective mining* (legacy set, $N{=}2{,}000$, assume $p{=}3\%$): a top $1\%$ review list ($f{=}0.01$, 20 compounds) at EF@1%$\,{=}\,7$ yields $\approx 7 \times 2{,}000 \times 0.01 \times 0.03 \approx 4.2$ true prodrugs among 20 candidates, turning an unmanageable archive into a focused, high-yield re-profiling slate. In each case, EF directly governs list size and expected yield, allowing teams to select *f* (e.g., $1\%$ vs. $5\%$) to balance synthesis throughput against discovery rate for the task at hand.

In summary, the early–recognition results are consistent across folds and the fixed test split. At *EF@1%*, several conceptually distinct learners exceed the practical threshold of 5 (e.g., LightGBM, XGBoost, ExtraTrees, Random Forest, Bagging, MLP, SVM–RBF, Voting), indicating that strong ultra-early retrieval is *not* confined to a single modeling family. When the window widens to *EF@5%*, the signal largely persists (notably for tree/ensemble and neural baselines such as LightGBM, ExtraTrees, XGBoost, Bagging, MLP, Random Forest), supporting the absence of model-selection bias and the robustness of the screening behavior. Crucially, the late-fusion ensemble sits *at or above* the corresponding base models across most folds—often by $\sim \!+0.5$ to $+\!1.0$ EF points—with a more pronounced margin at EF@5% than EF@1%, thereby raising the early-enrichment plateau and reducing variance. In aggregate, LightGBM (the framework default) and ExtraTrees are among the strongest single-model baselines, yet the simple 1:1 ensemble generally matches or surpasses both. Overall, Prodrug-ML, especially late fusion multimodel feature selection (default), delivers reliable early detection at the top 1–5% of ranked lists and is well-suited for operational prodrug screening.

#### Rank concentration: $\textrm{BEDROC}_\lambda$ ($\lambda \in \{20,50,80\}$)

**Fig. 4:**
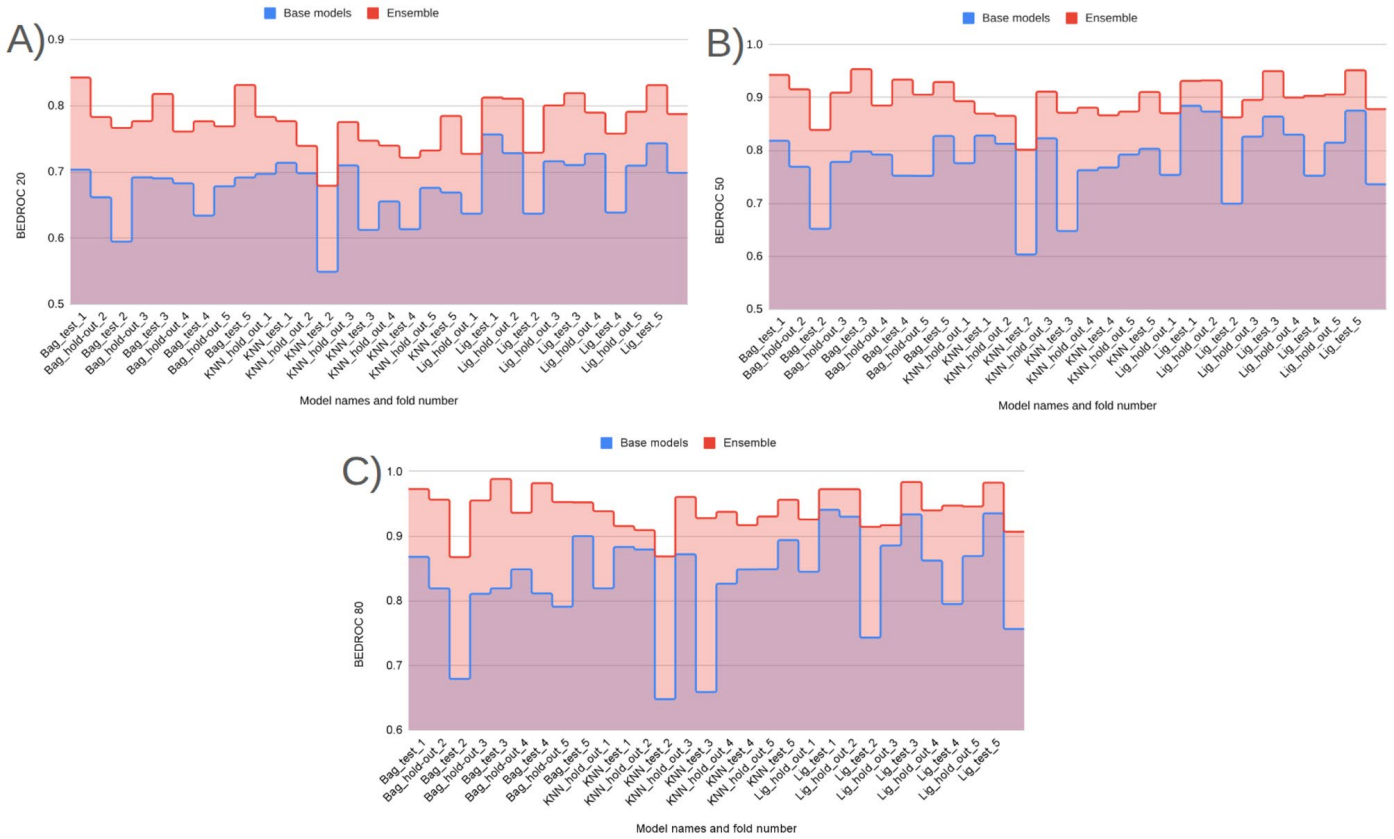
BEDROC at three values of $\lambda$: (A) $\lambda \!=\!20$, (B) $\lambda \!=\!50$, and (C) $\lambda \!=\!80$, comparing base models (blue) and the multimodel ensemble (red) for Bagging, k-NN, and LightGBM (default classifier) over all hold-out and early-split test folds. The figure has three panels: A reports $\textrm{BEDROC}_{20}$, B reports $\textrm{BEDROC}_{50}$, and C reports $\textrm{BEDROC}_{80}$. The x-axis lists model family and split/fold identifiers (e.g., Bag_hold-out_1...5, Bag_test_1...5, then k-NN_*, then Lig_*), while the y-axis shows the BEDROC value in [0, 1]. In each panel, the blue line/area summarizes the base models per fold, the red line/area shows the corresponding ensemble, and the purple overlap indicates agreement; the legend at the top labels “Base models” (blue) and “Ensemble” (red). To minimize meta-model complexity and keep the focus on the main contribution—principled construction of negatives—the ensemble combines base learners using a simple 1:1 linear averaging scheme

BEDROC performance of Prodrug-ML (Fig. 4), visual averages across the fixed early-split protocol (unchanged hold-out and external test) show a consistent family-wise ordering: LightGBM > Bagging > k-NN, so LightGBM is the default classifier in the Prodrug-ML framework. For $\lambda {=}20$ the base scores cluster around $\textrm{BEDROC}_{20}\!\approx \!0.70{-}0.75$ (LightGBM near $\sim 0.75$, Bagging $\sim 0.72$, k-NN $\sim 0.66$ with occasional dips to $\sim 0.55$). These figures, particularly the $\textrm{BEDROC}_{20}$ values for LightGBM, indicate that a true prodrug is typically retrieved within the top $\sim$8–10% of ranked candidates. Fortunately, for $\lambda {=}50$ they rise to $\textrm{BEDROC}_{50}\!\approx \!0.78{-}0.83$ (LightGBM $\sim 0.83$, Bagging $\sim 0.80$, k-NN $\sim 0.73$ with dips near $\sim 0.60$). These results of the $\textrm{BEDROC}_{50}$ values for LightGBM, suggest that a true prodrug is commonly ranked within the top $\sim$5% of candidates, Also, for $\lambda {=}80$ to $\textrm{BEDROC}_{80}\!\approx \!0.84{-}0.89$ (LightGBM $\sim 0.89$, Bagging $\sim 0.86$, k-NN $\sim 0.80$ with dips $\sim 0.65$). Besides the performance of three classifiers, several non-similarity learners achieve a strong BEDROC across the fixed early-split protocol (Supplementary Information). Averaged over folds, XGBoost attains ${\textrm{BEDROC}}_{20/50/80}\approx 0.70/0.80/0.84$, CatBoost 0.69/0.78/0.82, ExtraTrees 0.68/0.78/0.83, Bagging 0.67/0.77/0.82, and MLP 0.67/0.77/0.82; Random Forest and Voting follow closely ($\sim 0.66/0.76/0.80$ and $\sim 0.66/0.75/0.79$, respectively) (Supplementary Information). These values indicate that multiple, conceptually distinct models concentrate true prodrugs near the top of the ranking not only at $\lambda {=}20$ but also at stricter early-recognition settings ($\lambda {=}50,80$). Linear baselines and shallow trees show lower averages (e.g., Logistic regression families $\sim 0.48{-}0.52$, single Decision Tree $\sim 0.45$ at $\lambda {=}80$), reinforcing that interaction-capable ensembles and neural models are better aligned with the task’s discrete, bioreversible modification patterns. Also, these BEDROC trends mirror the EF@1%/EF@5% findings, support the absence of model-selection bias, and substantiate that Prodrug-ML’s late-fusion ensemble sits at or above strong single-model baselines such as LightGBM and ExtraTrees across early-recognition regimes. Overall, at this highest early-recognition stringency, the $\textrm{BEDROC}_{80}$ values demonstrate that Prodrug-ML consistently ranks true prodrugs within the highly top tier of candidates (often top $\sim$3-4%), underscoring its capacity to sharply concentrate relevant molecules at the forefront of virtual screening libraries. Given that real screening libraries often contain thousands of compounds, Prodrug-ML thus substantially narrows the search space by eliminating the majority (more than 95%) of non-prodrugs, thereby facilitating efficient identification of promising candidates. Overall, the base families exhibit robust and significant early recognition, with k-NN showing the largest fold-to-fold variability.

The ensemble traces lie at or above the bases across nearly all folds in all three panels (Fig. 4), with absolute gains of roughly $+\!0.05{-}0.10$ at $\lambda {=}20$, $+\!0.10{-}0.15$ at $\lambda {=}50$, and $+\!0.08{-}0.12$ at $\lambda {=}80$. For example, where bases dip to $\sim 0.55{-}0.60$ at $\textrm{BEDROC}_{20}$ (some k-NN folds), the ensemble remains around $\sim 0.75$; and where LightGBM (default classifier) bases reach $\sim 0.88{-}0.90$ at $\textrm{BEDROC}_{80}$, the ensemble commonly approaches $\sim 0.95{-}0.99$. These uplifts reflect the multimodel feature-selection aggregating complementary signals and damping model-specific errors, which raises the early-ranking plateau and reduces variance. Practically, the ensemble’s higher $\textrm{BEDROC}_{20/50/80}$ implies that actives are pushed even closer to the extreme top ($\sim 8\%\!\rightarrow \!\sim 3\%\!\rightarrow \!\sim 2\%$ windows), indicating that Prodrug-ML further strengthens early-retrieval performance over already-strong bases and is well-suited for prodrug screening thanks to significant BEDROC values (Fig. 4).

As for the interpretation of the BEDROC results, BEDROC provides a direct way to plan *how early* to test by tying the emphasis parameter $\lambda$ to an effective top-fraction window (about $f \approx \ln (5)/\lambda$; i.e., $\lambda {=}20 \Rightarrow f{\sim }8\%$, $\lambda {=}50 \Rightarrow f{\sim }3.2\%$, $\lambda {=}80 \Rightarrow f{\sim }2\%$). The observed ensemble scores (e.g., $\textrm{BEDROC}_{20}{\approx }0.78$–0.82, $\textrm{BEDROC}_{50}{\approx }0.90$–0.95, $\textrm{BEDROC}_{80}{\approx }0.95$–0.99) indicate progressively tighter concentration of true prodrugs within those early windows. Practically:

- Lead optimization (small enumerations): With $N{=}300$ enumerated variants and a synthesis budget near $3\%$ ($\approx 9$ designs), targeting high $\textrm{BEDROC}_{50}$ (e.g., $\ge 0.90$) justifies testing only the top ${\sim }3\%$, because the ranking concentrates actives within that window. Fortunately, with $\textrm{BEDROC}_{50}\!\approx \!0.90$, Prodrug-ML is expected to retain at least one true prodrug in the top ${\sim }3\%$ while excluding the remaining ${\sim }97\%$, thereby saving ${\sim }97\%$ of synthesis time and funds.
- Library design (medium/large screens): For $N{=}20{,}000$ and a first-pass assay budget of $2\%$ ($\approx 400$ compounds), $\textrm{BEDROC}_{80}{\ge }0.95$ supports an *experimentally-early* pull (top ${\sim }2\%$), prioritizing compounds most likely to be prodrugs while deferring the long tail. Operationally, this strategy avoids assaying ${\sim }98\%$ of the library at this stage, translating to ${\sim }98\%$ savings in initial screening cost and time.
- Retrospective mining (archive triage): For $N{=}2{,}000$ legacy compounds and a review quota of $2\%$ (40 entries), a strong $\textrm{BEDROC}_{80}$ allows curators to restrict manual inspection to a compact, high-yield slice at the extreme top of the ranking. This limits curation to only ${\sim }2\%$ of entries and can defer ${\sim }98\%$, yielding ${\sim }98\%$ savings in immediate review effort while preserving the most promising candidates.

Unlike EF, which translates directly into expected uplift in the top-*f* set, BEDROC is a *planning dial*: choose $\lambda$ to match the budgeted top-fraction and use the achieved $\textrm{BEDROC}_\lambda$ to judge whether actives are sufficiently front-loaded to justify that shortlist size. In practice, high $\textrm{BEDROC}_{50/80}$ permits aggressive early pulls (top $3\%$ or $2\%$), whereas more modest $\textrm{BEDROC}_{20}$ would motivate slightly broader early windows (top ${\sim }8$–$10\%$) to maintain recall in the first batch.

In summary, across the fixed early–split protocol, Prodrug-ML exhibits strong rank concentration of true prodrugs at all three emphasis levels. Base learners follow a consistent ordering (LightGBM > Bagging > k-NN), with LightGBM delivering the highest $\textrm{BEDROC}_{20/50/80}$ and k-NN showing the greatest fold-to-fold variability. Crucially, this pattern is not unique to a single family: multiple non-similarity baselines (e.g., XGBoost, CatBoost, ExtraTrees, Random Forest, MLP, Voting (Supplementary Information)) achieve high BEDROC, with mean $\textrm{BEDROC}_{50}$ and $\textrm{BEDROC}_{80}$ typically in the $\sim \!0.75{-}0.85$ and $\sim \!0.82{-}0.90$ bands, respectively, corroborating that early rank concentration is *not* model-contingent. Moreover, the late-fusion ensemble (1:1 linear averaging) consistently lifts BEDROC over its constituent bases—by roughly $+\!0.05{-}0.15$ depending on $\lambda$—with the margin most pronounced at $\lambda \!=\!50$ and $\lambda \!=\!80$. In practical terms, ensemble $\textrm{BEDROC}_{50/80}$ in the upper-0.8s implies that true prodrugs are regularly front-loaded into the top $\sim \!3\%\!-\!5\%$ of ranked candidates, sharply narrowing the experimental search space. These results confirm that Prodrug-ML delivers reproducible, high-stringency early recognition and that simple ensembling over strong neural/tree baselines provides a robust advantage for prodrug-screen prioritization.

#### Global and thresholded metrics: ROC AUC, average precision, and F1

**Fig. 5:**
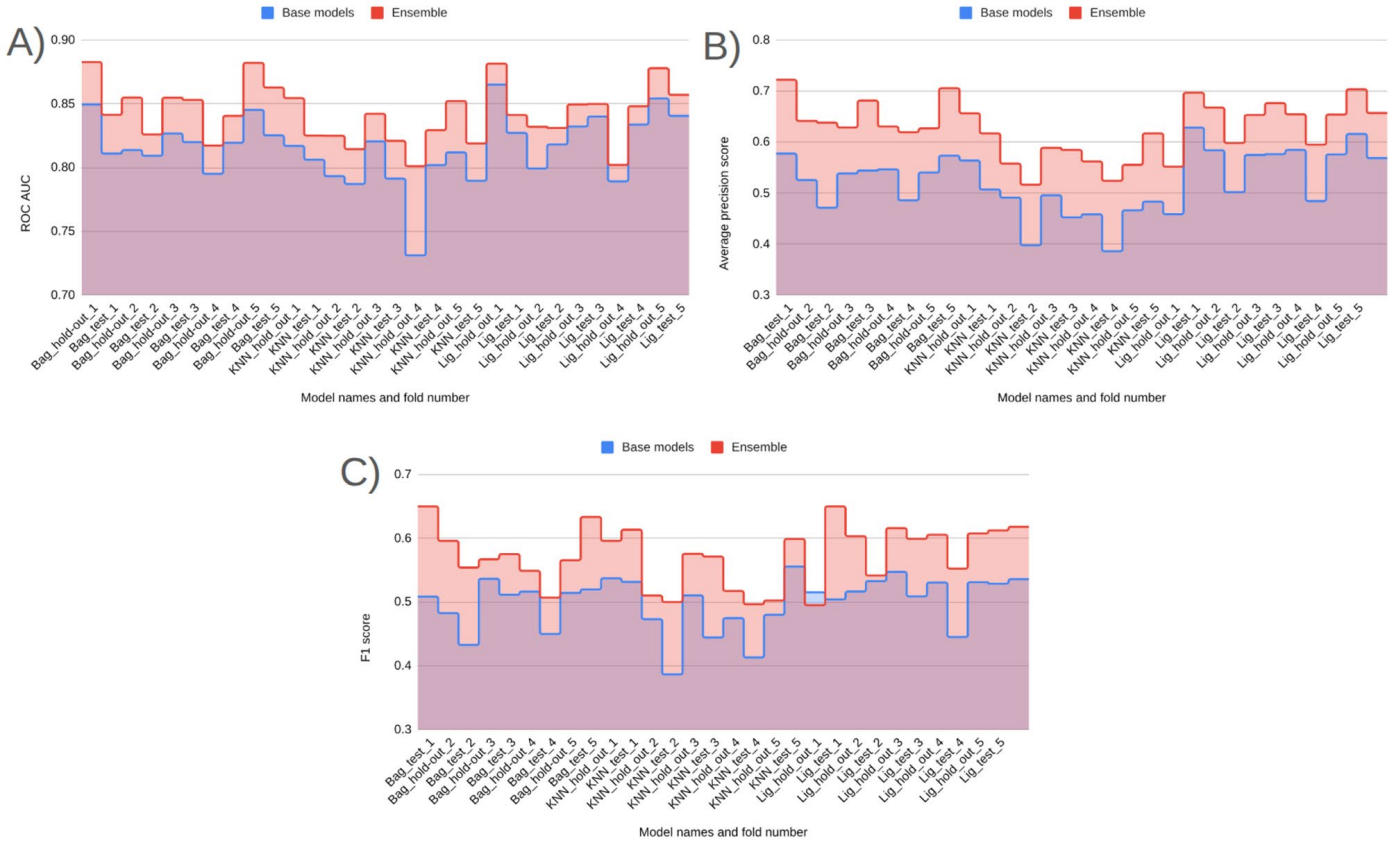
Model-discrimination and classification metrics for three model families (Bagging, k-NN, and LightGBM (default classifier in Prodrug-ML)): base models (blue) versus the multimodel ensemble (red) over all hold-out and early-split test folds. The figure has three panels: A reports ROC AUC (threshold-free ranking quality), B reports Average Precision (area under the precision–recall curve, informative under class imbalance), and C reports F1 score computed at the default decision threshold (0.5). The x-axis lists model family and split/fold identifiers (e.g., Bag_hold-out_1...5, Bag_test_1...5, then k-NN_*, then Lig_*), while the y-axis shows the corresponding metric value. In each panel, the blue line/area summarizes base-model performance per fold; the red line/area shows the ensemble; the purple overlap indicates agreement. Ensemble outputs are computed as a simple equal-weight (1:1) linear average of the base learners, intentionally forgoing meta-level tuning to maintain transparency and keep the spotlight on the study’s primary contribution—constructing reliable negative cohorts

As for the model metrics (Fig. 5), across the fixed early-split protocol (unchanged hold-out and external test), the base families show a consistent ranking with LightGBM (default classifier) highest, Bagging close, and k-NN lower with larger variance. In Fig. 5 Panel A (ROC AUC), bases cluster around $\sim 0.80{-}0.85$ overall, with LightGBM reaching $\sim 0.85$ and k-NN dipping near $\sim 0.75$ in some folds. Also, Fig. 5 Panel B (AP) shows wider spread under class imbalance: typical base values are $\sim 0.50{-}0.60$, with occasional k-NN dips to $\sim 0.45{-}0.48$ and LightGBM around $\sim 0.58{-}0.62$ (Fig. 5). Moreover, Fig. 5 panel C (F1 at threshold 0.5) places most base points in the $\sim 0.48{-}0.55$ band, with sporadic lows near $\sim 0.42{-}0.45$ (Fig. 5). Taken together, the bases provide strong ranking quality (ROC AUC), moderate precision–recall performance (AP), and mid$-$0.5 F1, with k-NN contributing the largest fold-to-fold fluctuations.

As for the contribution of multimodel features selection, in Panel A (Fig. 5), ROC AUC typically rises by about $+\,\!0.02\,{-}\,0.04$ (e.g., moving $\sim 0.83\!\rightarrow\, \!\sim 0.86\,{-}\,0.87$); in Panel B the lift is larger—about $+\,\!0.08\,{-}\,0.15$ AP—particularly where base k-NN dips ($\sim 0.45\,{-}\,0.50$) but the ensemble remains near $\sim 0.60\,{-}\,0.65$; and in Panel C F1 improves by roughly $+\,\!0.05\,{-}\,0.10$, with many folds advancing from $\sim 0.50$ to $\sim 0.58\,{-}\,0.62$. These gains reflect that the multimodel feature-selection enhanced the performance of Prodrug-ML by overcoming domain bias and leaking challenges. Therefore, Prodrug-ML, as a pioneer model, shows promising and robust performance in prodrug screening.

In summary, as the first dedicated prodrug-likeness screening framework, Prodrug-ML delivers consistently strong early-recognition and discrimination across the fixed early-split protocol. The ensemble surpasses the bases in nearly every fold, clearing practical enrichment bars with EF@1% $\ge 6$ and EF@5% $\ge 5$ while adding roughly $+\,\!0.5\,{-}\,1.0$ EF points on average; BEDROC further corroborates the early-rank concentration with ensemble scores around $\textrm{BEDROC}_{20}\!\approx \!0.78{-}0.82$, $\textrm{BEDROC}_{50}\!\approx \!0.90{-}0.95$, and $\textrm{BEDROC}_{80}\!\approx \!0.95{-}0.99$. Threshold-free and classification metrics align with these gains, with typical lifts of $+\,\!0.02\,{-}\,0.04$ AUC, $+\,\!0.08\,{-}\,0.15$ AP, and $+\,\!0.05\,{-}\,0.10$ F1, alongside reduced fold-to-fold variance. Collectively, these results indicate that Prodrug-ML achieves significant, operationally meaningful performance for prodrug screening. Although the performance of Prodrug-ML has been validated using different metrics, the benchmarks and decoys should be validated to confirm performance enhancement.

### Validation of the dataset

In prodrug-likeness, apparent gains may arise from (i) Controlling Performance of No-Feature Selection Pipeline: trains the LightGBM classifier on the full 2048-bit Avalon fingerprint to probe whether high scores can be produced by memorizing split/recipe artifacts; (ii) Domain-bias Analysis: fits an auxiliary “origin” classifier to predict the feature-view/recipe source and quantifies Accuracy, Balanced Accuracy, Macro-F1, and Macro AUC (OVR) to detect learnable domain cues; (iii) Cross-decoy Validation and Feature Set Selection: evaluates transfer when the decoy recipe changes between train and test, guiding the selection of robust views; (iv) Evaluation of Feature Set Selection (EF & BEDROC across Top-6 Ensembles): validates the chosen ensemble by reporting EF@1/5% and $\text {BEDROC}_{\{20,50,80\}}$ for the six best feature-set combinations, using equal linear weighting within each ensemble; (v) Summary of the Validation: synthesizes the above evidence to justify the reliability of the dataset and the suitability of the final Prodrug-ML configuration for early prodrug screening. To ensure that the results reflect genuine capability rather than dataset shortcuts, Prodrug-ML has been validated along five complementary axes: (i) Controlling Performance of No-Feature Selection Pipeline, (ii) Domain-bias Analysis, (iii) Cross-decoy Validation and Feature Set Optimization, (iv) Evaluation of Feature Set Optimization, and (v) Summary of the Validation of the Dataset.

#### Controlling performance of no-feature selection pipeline

Training the default Prodrug-ML classifier (LightGBM) on the full 2048-bit Avalon fingerprint (i.e., without feature selection) yields uniformly strong early-recognition and global scores across all fixed early-splits. On the *external tests* (splits 0–4) (Table 2), the per-split performance concentrates at $\mathrm {EF@1}\%$ = 7000 for every split; $\mathrm {EF@5}\%$ =6.58 ± 0.13 (range 6.349–6.674); $\textrm{BEDROC}_{20}$ = 0.899 ± 0.009, (0.891–0.914) $\textrm{BEDROC}_{50}$ = 0.962 ± 0.004 (0.955–0.966) , and $\textrm{BEDROC}_{80}$ = 0.983 ± 0.003 (0.978–0.987) (Table 2). Global classification metrics are likewise high with $\textrm{ROC}\ \textrm{AUC}=0.947\pm 0.011$ $(0.925\text {--}0.957)$, $\textrm{AP}=0.826\pm 0.017$ $(0.805\text {--}0.852)$, and $\textrm{F1}=0.738\pm 0.016$ $(0.712\text {--}0.757)$ (Table 2). Hold-out folds show a similar plateau (e.g., mean $\textrm{ROC}\ \textrm{AUC}=0.949$, $\textrm{AP}=0.838$, $\textrm{BEDROC}_{50}=0.980$, $\textrm{BEDROC}_{80}=0.995$) (Table 2). Numerically, these all-feature results sit *above* both the LightGBM base model (with selected features) and Prodrug-ML (the late-fusion ensemble with LightGBM) reported in the comparative analysis. However, a no-feature selection pipeline can suffer from the leak between groups in domains, which results in high performance. Since the full 2048-bit representation is rich enough to encode split- or source-specific artifacts, and an all-feature LightGBM can inadvertently learn dataset provenance cues, yielding deceptively high early-window and global scores. Therefore, it should be validated in a domain-bias leak to validate its high performance than the other pipelines, such as the LightGBM base (with selected features) and the late-fusion ensemble models.

**Table 2:** Per-split early-recognition and global metrics for the LightGBM default classifier in Prodrug-ML trained with the full 2048-bit Avalon fingerprint feature set (no feature selection), evaluated under the fixed early-split protocol with unchanged hold-out and external test partitions

Split	EF@1%	EF@5%	BEDROC 20	BEDROC 50	BEDROC 80	ROC AUC	AP	F1
1 (Hold-out)	6.980	6.980	0.936	0.992	0.999	0.959	0.875	0.759
1 (Test)	7.000	6.512	0.891	0.965	0.987	0.944	0.814	0.712
2 (Hold-out)	7.041	6.438	0.887	0.959	0.984	0.944	0.815	0.713
2 (Test)	7.000	6.674	0.892	0.962	0.983	0.925	0.805	0.757
3 (Hold-out)	7.041	7.041	0.914	0.984	0.997	0.955	0.843	0.747
3 (Test)	7.000	6.674	0.905	0.961	0.980	0.955	0.838	0.730
4 (Hold-out)	6.969	6.969	0.889	0.976	0.995	0.943	0.807	0.679
4 (Test)	7.000	6.674	0.914	0.966	0.985	0.957	0.852	0.752
5 (Hold-out)	6.969	6.969	0.926	0.988	0.998	0.946	0.851	0.766
5 (Test)	7.000	6.349	0.893	0.955	0.978	0.952	0.824	0.738

Reported metrics are EF@1%, EF@5%, BEDROC at $\lambda \in \{20,50,80\}$, ROC AUC, Average Precision (AP), and F1 for splits 0-4. The table documents, for a single model configuration (LightGBM with identical hyperparameters across splits and all Avalon bits), the complete set of ranking-focused (EF, BEDROC) and classification-focused (ROC AUC, AP, F1) metrics on both the hold-out and the external test for each split. Presenting the per-split values provides transparent traceability of the evaluation protocol, enables exact reproduction, and allows independent aggregation or secondary analyses without introducing additional assumptions. No feature selection is applied here by design, and the same preprocessing/evaluation pipeline used throughout the study is retained so that these results are directly comparable in format to the main experiments

#### Domain-bias Analysis

Domain bias arises when an auxiliary “origin” classifier can tell which split or decoy recipe a molecule belongs to from non-task-relevant cues (e.g., scaffold leakage, series effects, or imbalanced physicochemical profiles), thereby inflating apparent performance. For the single-view feature sets (Table 3), *List_1* and *List_2*, chance accuracy for a three-class origin task is $$\approx 0.33$$; the observed means ($$\textrm{Accuracy}\approx 0.37{-}0.39$$, $$\textrm{Balanced}\,\textrm{Accuracy}\approx 0.37{-}0.39, $$$$\textrm{Macro}\,\textrm{F1}\approx 0.37\,{-}\,0.39$$) sit only slightly above this baseline with small standard deviations, while $$\textrm{Macro}\,\textrm{AUC}\,\textrm{OVR}\approx 0.56{-}0.57$$ is close to the 0.50 no-skill level (Table 3). Taken together, these near-chance results are consistent with effectively random discrimination and provide no substantive evidence of domain bias in the individual views.

By contrast, naïve early fusion (*List_1+List_2*) elevates the origin signal: Accuracy $\approx 0.462$, Balanced accuracy $\approx 0.462$, Macro F1$\approx 0.461$, and Macro AUC OVR $\approx 0.636$ (Table 3). The figures indicate that concatenating raw features before training can expose weak-to-moderate domain-specific regularities. Fortunately, the multimodel ensemble strategy used in Prodrug-ML—feature selection per view followed by linear weighting at the score level (late fusion)—mitigates such exposure by aggregating complementary signals while averaging out spurious, view-specific artifacts. Although the *List_1+List_2* block shows more domain signal than single views, the magnitude remains tolerable and non-critical domain bias. Therefore, it can be an alternative version of Prodrug-Ml with an acceptable but higher domain bias model.

**Table 3:** The summary of the domain-bias audit using Accuracy, balanced accuracy, macro F1, and macro AUC (one-vs-rest) for each feature view (List_1, List_2), their combination (List_1+List_2), and the full fingerprint set (all data, 2048 features)

List names		Accuracy	Balanced accuracy	Macro F1	Macro AUC OVR
List_1	Mean	0.369	0.369	0.369	0.560
	Std	0.027	0.027	0.027	0.021
List_2	Mean	0.390	0.390	0.390	0.573
	Std	0.013	0.013	0.013	0.020
List_1+List_2	Mean	0.462	0.462	0.461	0.636
	Std	0.025	0.025	0.026	0.021
All data (2048)	Mean	0.597	0.597	0.595	0.787
	Std	0.006	0.006	0.007	0.010

Each block corresponds to a feature configuration used by the auxiliary classifier in the domain-bias audit: List_1 and List_2 are separate views; List_1+List_2 is their concatenation; All data (2048) uses the full 2048-dimensional fingerprint set. Within each block, Mean and Std are the average and standard deviation computed over the cross-validation folds/runs. Accuracy is the overall proportion of correct assignments across classes; Balanced Accuracy is the average of per-class recalls (accounts for class imbalance); Macro F1 is the unweighted mean of per-class F1 scores; and Macro AUC OVR is the macro-averaged area under the ROC curve using a one-vs-rest formulation

Using the full fingerprint space (*All data (2048)*) produces a clearly concerning level of origin predictability: $\textrm{Accuracy}\approx 0.597$, $\textrm{Balanced}\,\textrm{Accuracy}\approx 0.597$, $\textrm{Macro}\,\textrm{F1}\approx 0.595$, and $\textrm{Macro}\,\textrm{AUC}\,\textrm{OVR}\approx 0.787$. Such values imply that high-dimensional representations contain abundant dataset-specific cues that a model could exploit, emphasizing that judicious feature selection and multimodel ensembling are not optional embellishments but central mechanisms in Prodrug-ML for suppressing domain bias and preserving trustworthy generalization. Consequently, the full fingerprint space results prove that Prodrug-ML, a well-designed framework, has been learning data instead of memorizing with the help of domain bias.

As for the the confusion matrices to deeply investigate domain-bias for each domain source, Table 4 shows that for the single-view configurations, *List_1* and *List_2*, the confusion matrices are close to chance: in *List_1*, the diagonal entries are 359/976 = 36.8% (ChEMBL_high_conf), 373/976 = 38.2% (ChEMBL_random), and 348/976 = 35.7% (DUDE); in *List_2*, they are 389/976 = 39.9%, 377/976 = 38.6%, and 375/976 = 38.4%, respectively. Off-diagonal counts remain comparable across columns, and column totals are broadly balanced, indicating no critical domain-specific signal in either view. If any bias appears, it is mild and most visible on the ChEMBL_random row (e.g., 373/976 and 377/976), yet still near the 33% chance level and thus not operationally concerning. Therefore, the performance of Prodrug-ML on the unseen test set has been validated by controlling domain bias in the data.

**Table 4:** The confusion matrices of predicted origin class versus ground-truth origin class for each feature configuration (List_1, List_2, List_1+List_2, and All features, 2048)

List number	Ground truth	Prediction (ChEMBL_high_conf)	Prediction (ChEMBL_random)	Prediction (DUDE)
List_1	True (ChEMBL_high_conf)	359	304	313
	True (ChEMBL_random)	303	373	300
	True (DUDE)	336	292	348
List_2	True (ChEMBL_high_conf)	389	287	300
	True (ChEMBL_random)	315	377	284
	True (DUDE)	327	274	375
List_1+List_2	True (ChEMBL_high_conf)	439	241	296
	True (ChEMBL_random)	242	495	239
	True (DUDE)	315	243	418
All features (2048)	True (ChEMBL_high_conf)	543	186	247
	True (ChEMBL_random)	154	689	133
	True (DUDE)	297	164	515

Each block is a $3\times 3$ confusion matrix produced by the auxiliary “origin” classifier used in the domain-bias audit. Rows encode the ground-truth origin class (*ChEMBL_high_conf*, *ChEMBL_random*, *DUDE*); columns encode the predicted origin class under the same three labels. Cell entries are sample counts; values on the main diagonal (top-left to bottom-right) correspond to correct origin assignments, while off-diagonal entries indicate cross-confusions between origins. The four stacked blocks correspond to different feature configurations evaluated by the origin classifier: *List_1* and *List_2* are single-view feature sets, *List_1+List_2* is their concatenation (early fusion), and *All features (2048)* uses the full 2048-dimensional fingerprint vector. Within each block, the three rows sum to the total number of ground-truth instances across the origin classes, and the three columns sum to the total number of predictions made for each origin class

Naïve early fusion by concatenation (*List_1+List_2*) amplifies domain signal: the diagonal rises to $439/976=45.0\%$ (ChEMBL_high_conf), $495/976=50.7\%$ (ChEMBL_random), and $418/976=42.8\%$ (DUDE) (Table 4). The ChEMBL_random row illustrates the effect clearly, with correct-origin assignments increasing by $\Delta = +122$ (vs. *List_1*) and $\Delta = +118$ (vs. *List_2*), while off-diagonals drop to 242 and 239, indicating stronger separability by origin. This pattern is consistent with domain-based knowledge leaking into the learned representation when views are merged before training: once a feature becomes correlated with a particular decoy domain, a classifier can over-rely on it and more readily assign the corresponding (often negative) label. In contrast, the multimodel ensemble strategy employed in Prodrug-ML—feature selection within each view and linear weighting at the score level—aims to aggregate complementary signals while averaging out view-specific artifacts, thereby reducing the opportunity for such leakage.

Using the full 2048-dimensional fingerprint set (*All features (2048)*) produces a clearly concerning level of domain predictability: diagonals reach $543/976=55.6\%$ (ChEMBL_high_conf), $689/976=70.6\%$ (ChEMBL_random), and $515/976=52.8\%$ (DUDE)—roughly $1.5{-}2.1\times$ chance (Table 4). Relative to the single views, the ChEMBL_random row increases from 373/976–377/976 ($\approx 38\%$) to 689/976 ($\approx 71\%$), a $\sim$1.8$\times$ jump (Table 4). These counts indicate that high-dimensional, unfocused representations encode abundant domain-specific cues; accordingly, feature selection and late-fusion ensembling are not merely convenient but essential components of Prodrug-ML for suppressing domain bias and preserving trustworthy generalization.

In summary, the domain-bias audit indicates that the single-view configurations (List_1, List_2) behave near chance across accuracy, balanced accuracy, and macro F1, with confusion matrices close to uniform; naïve early fusion (List_1+List_2) introduces a modest origin signal, and the full 2048-feature representation reveals strong domain cues. Because Prodrug-ML employs per-view feature selection with late-fusion ensembling, the deployed system does not exhibit concerning domain bias; accordingly, the reported EF and BEDROC gains reflect genuine early-recognition capability rather than dataset artifacts, supporting the validity and robustness of the overall performance.

#### Cross-decoy validation and feature set optimization

Cross-decoy results show the expected gap between in-recipe validation and true transfer, with Average Precision (AP) being more sensitive to recipe shifts than ROC AUC (Table 5). For List_1, same-recipe *cv_holdout* reaches AUC/AP of 0.84/0.71 (ChEMBL_high_conf), 0.801/0.627 (ChEMBL_random), and 0.829/0.671 (DUDE), whereas cross-decoy *external_test* drops AP to 0.256-$-$0.377 despite AUC staying in the 0.78–0.813 range (e.g., train DUDE $\rightarrow$ test ChEMBL_random: 0.787/0.256; train ChEMBL_high_conf $\rightarrow$ test DUDE: 0.811/0.377). List_2 exhibits stronger transfer: in-recipe *cv_holdout* sits at 0.808/0.675 (ChEMBL_high_conf), 0.79/0.657 (ChEMBL_random), and 0.804/0.688 (DUDE), while cross-decoy *external_test* maintains comparatively high AUC and notably higher AP than List_1 under the same shifts (e.g., train ChEMBL_random $\rightarrow$ test DUDE: 0.836/0.446; train ChEMBL_high_conf $\rightarrow$ test ChEMBL_random: 0.81/0.432; train DUDE $\rightarrow$ test ChEMBL_high_conf: 0.824/0.417) (Table 5). The best off-diagonal AUC appears for List_2 when training on ChEMBL_random and testing on ChEMBL_high_conf (0.845) with AP 0.473, indicating robust rank preservation and useful precision–recall behavior under recipe shift. Consequently, cross-decoy validation provides no evidence of problematic domain leakage, supporting the robustness of the training data and affirming that Prodrug-ML generalizes reliably to the independent test set.

**Table 5:** Cross-decoy validation for two feature views (List_1 and List_2): ROC AUC and average precision (AP) when training on one decoy recipe and evaluating on the same or a different recipe, with *cv_holdout* denoting within-fold validation and *external_test* denoting the fixed early-split test

List ID	TrainNeg	TestNeg	Test Type	ROC AUC	AP
List_1	ChEMBL_high_conf	ChEMBL_high_conf	cv_holdout	0.84	0.71
		ChEMBL_random	external_test	0.786	0.297
		DUDE	external_test	0.811	0.377
	ChEMBL_random	ChEMBL_high_conf	external_test	0.789	0.305
		ChEMBL_random	cv_holdout	0.801	0.627
		DUDE	external_test	0.780	0.295
	DUDE	ChEMBL_high_conf	external_test	0.813	0.305
		chembl_random	external_test	0.787	0.256
		DUDE	cv_holdout	0.829	0.671
List_2	ChEMBL_high_conf	ChEMBL_high_conf	cv_holdout	0.808	0.675
		ChEMBL_random	external_test	0.810	0.432
		DUDE	external_test	0.833	0.443
	ChEMBL_random	ChEMBL_high_conf	external_test	0.845	0.473
		ChEMBL_random	cv_holdout	0.790	0.657
		DUDE	external_test	0.836	0.446
	DUDE	ChEMBL_high_conf	external_test	0.824	0.417
		chembl_random	external_test	0.798	0.370
		DUDE	cv_holdout	0.804	0.688

Each block (List_1, List_2) enumerates all train$\rightarrow$test decoy pairings for three decoy recipes: *ChEMBL_high_conf*, *ChEMBL_random*, and *DUDE*. The *TrainNeg* column specifies the decoy recipe used to fit the model; *TestNeg* is the recipe used at evaluation. Rows labeled *cv_holdout* in *Test Type* correspond to same-recipe validation (the “diagonal”: TrainNeg = TestNeg) performed on cross-validated hold-out folds, while rows labeled *external_test* correspond to cross-decoy transfer (the “off-diagonals”: TrainNeg $\ne$ TestNeg) evaluated on the fixed, untouched early-split test set. *ROC AUC* reports threshold-free ranking quality, and *AP* (average precision) summarizes precision-recall performance under class imbalance. Thus, for each List ID, the table presents nine outcomes (3 train $\times$ 3 test) that jointly characterize in-recipe performance and robustness to shifts in the negative-set construction on only training data

Cross-decoy results (Table 5 show the expected gap between in-recipe validation and true transfer, with Average Precision (AP) being more sensitive to recipe shifts than ROC AUC. For List_1, same-recipe *cv_holdout* reaches AUC/AP of 0.84/0.71 (ChEMBL_high_conf), 0.801/0.627 (ChEMBL_random), and 0.829/0.671 (DUDE), whereas cross-decoy *external_test* drops AP to 0.256-$-$0.377 despite AUC staying in the 0.78–0.813 range (e.g., train DUDE $\rightarrow$ test ChEMBL_random: 0.787/0.256; train ChEMBL_high_conf $\rightarrow$ test DUDE: 0.811/0.377). List_2 exhibits stronger transfer: in-recipe *cv_holdout* sits at 0.808/0.675 (ChEMBL_high_conf), 0.79/0.657 (ChEMBL_random), and 0.804/0.688 (DUDE), while cross-decoy *external_test* maintains comparatively high AUC and notably higher AP than List_1 under the same shifts (e.g., train ChEMBL_random $\rightarrow$ test DUDE: 0.836/0.446; train ChEMBL_high_conf $\rightarrow$ test ChEMBL_random: 0.81/0.432; train DUDE $\rightarrow$ test ChEMBL_high_conf: 0.824/0.417). The best off-diagonal AUC appears for List_2 when training on ChEMBL_random and testing on ChEMBL_high_conf (0.845) with AP 0.473, indicating robust rank preservation and useful precision–recall behavior under recipe shift. Thus, cross-decoy evaluation establishes that List_2, unlike List_1, generalizes reliably across alternative negative constructions, ensuring that Prodrug-ML captures prodrug-relevant structure rather than recipe-specific artifacts.

Table 6 shows *feature-set selection* rather than cross-decoy transfer to design a multimodel feature selection structure. Using the codebase identifiers (*list_7*=*list_1*, *list_12*=*list_2*), the highest single-view ROC AUC appears at *list_7* (0.838) but with EF@1%=4.359, whereas *list_12* attains a markedly stronger early enrichment (EF@1%=5.633) at a similar AUC level (0.821) (Table 6). Simple early fusion of two or more views raises EF@1% relative to *list_7* while leaving AUC essentially flat in the 0.82-$-$0.83 band (e.g., *list_7_12*: AUC=0.830, EF@1%=4.996; *list_30_7_17_12*: AUC=0.824, EF@1%=4.954), and adding additional lists yields diminishing returns or even mild dilution (e.g., *list_26_17_16_28_7*: AUC=0.821, EF@1%=4.707). Overall, the figures indicate that (i) early enrichment is the dimension that benefits most from combining views, (ii) AUC saturates across configurations, and (iii) the most efficient and stable basis for Prodrug-ML is the two-view design centered on *list_7* (*list_1*) and *list_12* (*list_2*), which preserves top-tier AUC while securing higher EF@1% with minimal complexity.

**Table 6:** Mean ROC AUC and EF@1% for single- and multimodel feature configurations, using the list numbering from the codebase (note: *list_7* corresponds to *list_1* in the manuscript, and *list_12* corresponds to *list_2*)

List_number	Mean_ROC_AUC	Mean_EF@1
list_7	0.838	4.359
list_7_12	0.830	4.996
list_7_31	0.830	4.782
list_7_30	0.829	4.826
list_25_7_31	0.826	4.792
list_13_31_7	0.826	4.908
list_30_28_7	0.826	4.934
list_30_7_17_12	0.824	4.954
list_17_13_31_7	0.824	4.814
list_28_29_14_7	0.823	4.992
list_7_28_2_29_31	0.822	4.962
list_13_31_7_2_29	0.822	4.964
list_12	0.821	5.633
list_31	0.821	5.206
list_26_17_16_28_7	0.821	4.707

Each row denotes a feature configuration evaluated under the same protocol: a single view (e.g., *list_7*, *list_12*, *list_31*) or an underscore-delimited combination of views (e.g., *list_7_12*, *list_30_7_17_12*). The column *Mean_ROC_AUC* reports the average area under the ROC curve (threshold-free ranking quality), while *Mean_EF@1* reports the average enrichment factor at the top 1% of the ranked list (early-retrieval emphasis); means are computed across the evaluation folds/runs. The list identifiers match those in the accompanying Python script; for consistency with the rest of the manuscript, *list_7* maps to *list_1* and *list_12* maps to *list_2*

In summary, cross-decoy validation confirms robust transfer: while List_1 loses AP under recipe shift (down to 0.256–0.377 at AUC 0.78-$-$0.81), List_2 maintains stronger off-diagonal performance (AUC up to 0.845 with AP 0.473), indicating recipe-agnostic signal. Multimodel feature selection further shows that early enrichment is strengthened without inflating AUC—*list_12* (*list_2*) achieves EF@1%=5.633 at AUC=0.821 and simple fusions hover near EF@1%$\approx$5.0 with AUC $\approx$0.82-$-$0.83. Based on these results, there is no concerning recipe-specific dependence, and the Prodrug-ML design (two-view core) yields valid, reliable performance for prodrug-likeness screening.

#### Evaluation of feature set optimization

Prodrug-ML employs a linearly weighted ensemble that combines the two feature subsets list_7 and list_12, with equal weights (1:1), selected according to their hold-out performance on the training set with the help of an applied cross-decoy validation approach (Table 6). To validate that this choice is consistent with the cross-validation–driven feature selection, the six highest-ranked ensemble feature sets were further examined. All of these candidates satisfy strict domain-bias controls, exhibiting domain-classifier mean accuracy $<0.40$, balanced accuracy $<0.40$, macro-F1 $<0.40$, and macro-AUC-OVR $<0.60$, indicating negligible leakage from dataset-specific artifacts into the learned representations.

**Fig. 6:**
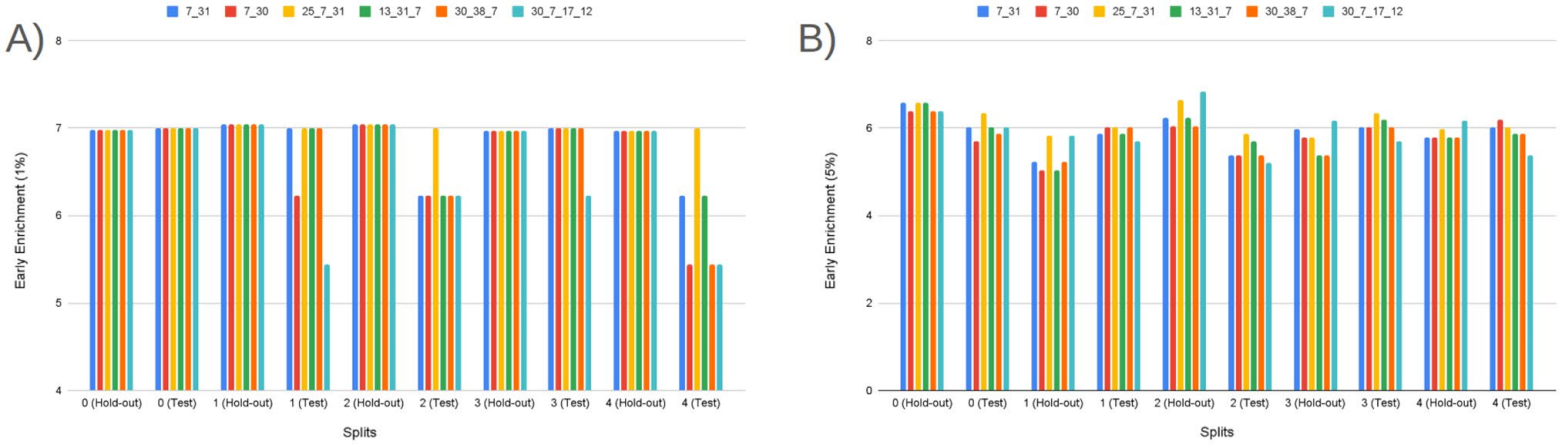
Early enrichment (EF) of the ensemble prodrug-likeness models across cross-validation splits, shown at 1%** A** and 5%** B** screening depths. The figure has two panels:** A** EF@1% and** B** EF@5%. The x-axis lists the five cross-validation splits, each shown for the corresponding “Hold-out” and “Test” evaluations. The y-axis reports the Early Enrichment score. Each color-coded bar group represents an ensemble configured with a specific feature-set combination; labels (e.g., 7_31, 25_7_31) denote the feature-set identifiers chosen during the cross-validation and feature-selection stage. Within each ensemble, model outputs are linearly averaged with equal weights based on ensemble size (e.g., two models: 0.5/0.5; four models: 0.25/0.25/0.25/0.25)

Early enrichment behavior for the chosen ensemble is summarized in Fig. 6: EF@1% values cluster near the upper portion of the axis (approximately $\sim 7$ across splits), and EF@5% values remain within a narrow band around $\sim 6$. The ensemble constructed using 25_7_31, the fifth option on cross-decoy validation results (Table 6), provided $\sim 7$ of EF@1% for all splits, not only on hold-out data, but also on test data. Having such an impressive performance from the fifth option on decoy-cross validation performance shows that the feature selection used in the Prodrug-ML framework has the opportunity to improve. Notably, of the three ensemble components (25_7_31), two (25_31) are not used elsewhere in Prodrug-ML, indicating that the gains arise from the ensemble architecture rather than dependence on any particular feature set. Overall, even if the other specific feature subsets used in the ensemble were to be replaced by alternative top-ranked combinations, the Prodrug-ML framework consistently yields strong early-detection performance suitable for rapid prodrug screening.

**Fig. 7:**
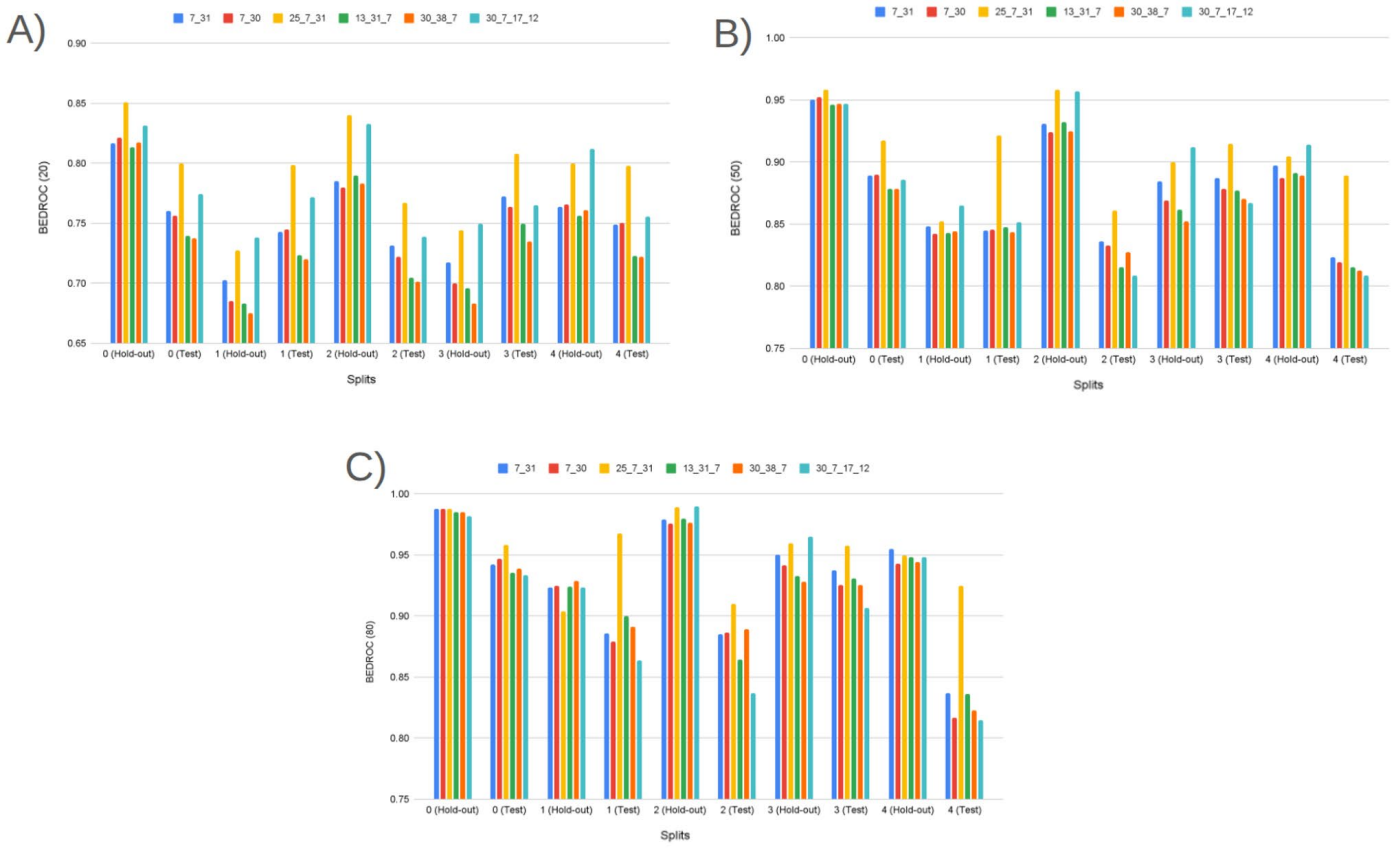
BEDROC-based early enrichment of the ensemble prodrug-likeness models across cross-validation splits at three concentration parameters: 20** A**, 50** B**, and 80** C**. The figure comprises three panels reporting BEDROC with $\alpha =20$** A**, $\alpha =50$** B**, and $\alpha =80$** C**. On the x-axis, the five cross-validation splits are listed, each shown for the corresponding “Hold-out” and “Test” evaluations. The y-axis displays the BEDROC score. Each color-coded bar group corresponds to an ensemble configured with a specific feature-set combination; labels (e.g., 7_31, 25_7_31) denote the identifiers of the feature sets selected during the cross-validation and feature-selection stage. Within every ensemble, model outputs are combined by equal linear weighting according to ensemble size (e.g., two models: 0.5/0.5; four models: 0.25/0.25/0.25/0.25)

In Fig. 7, Prodrug-ML (linearly weighting list_7 and list_12 with equal coefficients) attains BEDROC values in the ranges reported in Fig. 6: approximately 0.70-$-$0.85 at $\alpha \!=\!20$, around 0.90 at $\alpha \!=\!50$, and $>\!0.90$ at $\alpha \!=\!80$, where list_7+list_12 was selected due to the highest cross-decoy validation performance on the training set. To validate this selection, Fig. 7 compiles BEDROC results for the six top-ranked ensemble structures (7_31, 7_30, 30_38_7, 25_7_31, 17_13_31_7, 30_7_17_12). On the *test* partitions, BEDROC@20 spans $\sim$0.73$-$0.77 for 7_31, $\sim$0.72$-$0.75 for 7_30, $\sim$0.70$-$0.73 for 30_38_7, $\sim$0.77$-$0.81 for 25_7_31, $\sim$0.68$-$0.72 for 17_13_31_7, and $\sim$0.74$-$0.77 for 30_7_17_12. At $\alpha \!=\!50$, the corresponding test ranges are $\sim$0.82$-$0.89 (7_31), $\sim$0.82$-$0.89 (7_30), $\sim$0.81$-$0.88 (30_38_7), $\sim$0.89$-$0.92 (25_7_31), $\sim$0.79$-$0.87 (17_13_31_7), and $\sim$0.81$-$0.89 (30_7_17_12). At $\alpha \!=\!80$, test BEDROC values lie around $\sim$0.84$-$0.94 (7_31), $\sim$0.82$-$0.95 (7_30), $\sim$0.82$-$0.94 (30_38_7), $\sim$0.93$-$0.97 (25_7_31), $\sim$0.81$-$0.92 (17_13_31_7), and $\sim$0.82-$-$0.93 (30_7_17_12). Also, across $\alpha \!=\!20,50,80$, the alternative top-6 ensembles exhibit BEDROC profiles within high and relatively narrow bands, confirming that the feature-selection stage yields multiple competitive configurations. Consequently, even if the specific feature sets used by the production ensemble were replaced by another top-ranked combination, the Prodrug-ML framework would still deliver significant early-recognition capability for prodrug screening.

#### Summary of the validation of the dataset

The dataset was deeply investigated to ensure that the Prodrug-ML framework is valid and its reported gains reflect genuine prodrug-likeness rather than dataset shortcuts. The main findings are:

- All-features control exposes leakage: Training a LightGBM on the full 2048 -bit Avalon fingerprint (no feature selection) yields the highest headline metrics (Table 2) but exhibits strong domain predictability, indicating susceptibility to domain leakage and split/recipe artifacts (Tables 3, and 4).
- Confusion-matrix inspection aligns with metrics: Single-view confusion matrices remain close to chance across origin classes, while naïve early fusion increases origin predictability; Prodrug-ML avoids this by per-view feature selection and late fusion at the score level (Table 4).
- Leakage control via domain audit: The feature sets used in the final pipeline satisfy near-chance origin prediction: mean Accuracy $<0.40$, Balanced Accuracy $<0.40$, Macro-F1 $<0.40$, and Macro-AUC-OVR $<0.60$, evidencing domain-leak–free behavior under the auxiliary origin classifier (Tables 3 and 4).
- Cross-decoy validation guides selection. Models trained on one decoy recipe and tested on another maintain robust ROC AUC and practical AP for the selected views, demonstrating transferability beyond a single negative-set construction and guarding against recipe-specific shortcuts (Tables 5, and 6).
- Late fusion improves early recognition without inflating leakage: To enhance performance, base feature sets are combined by *linearly weighted late fusion* (equal weights per member); this boosts EF and BEDROC while preserving the low domain-bias profile seen in single-view audits (Figs. 3, 4, and 5).
- Top-6 ensemble consistency: EF@1%/5% and $\text {BEDROC}_{20/50/80}$ computed for the six highest-ranked ensembles show tightly clustered, high early-enrichment across hold-out and external tests, indicating that multiple feature-set combinations yield comparable behavior and the results are not brittle to a specific choice (Figs. 6, and 7).

Collectively, these checks—domain-bias auditing, cross-decoy transfer, late-fusion validation across top-ranked ensembles, and uncertainty quantification on fixed splits—establish that the dataset is appropriately controlled and that Prodrug-ML operates on chemically meaningful signal. The validated dataset, therefore, supports Prodrug-ML as a solid and reliable framework for early prodrug screening.

### Analysis of Prodrug-ML: factors behind the performance improvement

Understanding the fundamentals of Prodrug-ML contributes to the prodrug research and indicates further research by validating its performance and effectiveness. Therefore, five approaches have been used: (i) Label Randomness Analysis, (ii) Dimensionality Reduction with PCA, (iii) Non-Linear Feature Projection Using t-SNE, (iv) Model Interpretability with SHAP, and (v) Feature Importance Analysis (ANOVA F-test). Also, an extended analysis of Prodrug-ML has been completed under three analysis techniques, including (i) Correlation Heatmap, (ii) Hierarchical Feature Clustering (Dendrogram), and (iii) Multivariate Feature Visualization with RadViz (Supplementary Information). Finally, the analysis of Prodrug-ML has been summarized before the case study and limitations of the Prodrug-ML framework, as future opportunities.

#### Label randomness analysis

The label-randomization experiment demonstrates that the pipeline behaves exactly as expected in the absence of a learnable signal. Across families and splits, discrimination metrics hover at chance: ROC AUC $\approx 0.50$ (e.g., Bagging 0.501±0.033 hold-out; LightGBM (default classifier in Prodrug-ML) 0.510±0.037 test), Balanced Accuracy $\approx 0.50$, and MCC $\approx 0$ (Table 7). AP closely tracks the class prevalence (Random: 0.150 hold-out, 0.168 test), and the fitted models stay within that neighborhood (e.g., ListGBM 0.152±0.017 hold-out; 0.174±0.027 test). Early-enrichment metrics collapse to their null: EF@1% and EF@5% $\approx 1$ on average (with large variance at 1% due to tiny *k*), and $\text {BEDROC}_{20/50/80}$ concentrates near $\sim 0.15$ (e.g., Random 0.152; Bagging 0.154 ± 0.045 hold-out), matching the analytical expectation (Table 7). Small fluctuations (e.g., Bagging EF@1% $2.035\pm 1.269$ on test) reflect finite-sample effects and thresholding noise rather than genuine skill, consistent with AUC/BAL ACC staying at chance. These results (Table 7) jointly indicate that there is no leakage or hidden shortcut in the evaluation pipeline: when labels are randomized, models cannot extract signal and perform at chance across ranking, classification, calibration, and early-enrichment metrics. Consequently, the substantial gains observed with true labels (high EF@1/5%, elevated BEDROC, and improved AUC/AP) arise from patterns learned from the training set that transfer to unseen data, supporting the validity of Prodrug-ML’s reported performance.

**Table 7:** Label-randomization sanity check performance of three base families (Bagging, k-NN, LightGBM (default classifier in Prodrug-ML)) under shuffled labels on hold-out and test splits, reported as mean ±  SD across folds, alongside the analytic random-guess baseline (Random)

Metric	Bagging (hold-out)	Bagging (test)	k-NN (hold-out)	k-NN (test)	LightGBM (hold-out)	LightGBM (Test)	Random (hold-out)	Random (test)
ROC AUC	0.501 ± 0.033	0.504 ± 0.035	0.499 ± 0.030	0.501 ± 0.047	0.502 ± 0.033	0.510 ± 0.037	0.500	0.500
AP	0.152 ± 0.017	0.177 ± 0.028	0.147 ± 0.011	0.153 ± 0.018	0.152 ± 0.017	0.174 ± 0.027	0.150	0.168
F1	0.095 ± 0.036	0.147 ± 0.048	0.022 ± 0.021	0.031 ± 0.028	0.152 ± 0.035	0.191 ± 0.041	0.150	0.168
Precision	0.144 ± 0.052	0.230 ± 0.070	0.144 ± 0.133	0.201 ± 0.153	0.144 ± 0.033	0.182 ± 0.039	0.150	0.168
Recall	0.071 ± 0.029	0.108 ± 0.037	0.012 ± 0.012	0.017 ± 0.016	0.162 ± 0.039	0.202 ± 0.044	0.150	0.168
BAL ACC	0.501 ± 0.015	0.524 ± 0.020	0.500 ± 0.006	0.503 ± 0.008	0.501 ± 0.022	0.525 ± 0.025	0.500	0.500
Brier	0.155 ± 0.005	0.152 ± 0.006	0.136 ± 0.003	0.135 ± 0.005	0.184 ± 0.007	0.180 ± 0.008	0.128	0.140
MCC	0.001 ± 0.041	0.067 ± 0.054	0.002 ± 0.038	0.019 ± 0.047	0.001 ± 0.041	0.049 ± 0.049	0.000	0.000
Ef_1pct	1.104 ± 0.976	2.035 ± 1.269	1.036 ± 0.941	1.487 ± 1.046	1.036 ± 0.916	2.000 ± 1.227	1.000	1.000
Ef_5pct	1.004 ± 0.419	1.672 ± 0.572	1.001 ± 0.394	1.246 ± 0.473	1.038 ± 0.426	1.517 ± 0.508	1.000	1.000
BEDROC_20	0.154 ± 0.045	0.233 ± 0.061	0.153 ± 0.042	0.183 ± 0.053	0.155 ± 0.047	0.219 ± 0.059	0.152	0.152
BEDROC_50	0.149 ± 0.069	0.256 ± 0.095	0.146 ± 0.066	0.191 ± 0.082	0.148 ± 0.068	0.241 ± 0.090	0.143	0.143
BEDROC_80	0.152 ± 0.089	0.273 ± 0.121	0.146 ± 0.085	0.201 ± 0.105	0.147 ± 0.084	0.262 ± 0.117	0.143	0.143

Entries reflect a label-randomness experiment in which task labels are permuted to remove learnable signal; thus, well-behaved pipelines should yield chance-level metrics. Columns list common discrimination, calibration, and early-enrichment metrics: ROC AUC, AP (average precision), F1, Precision, Recall, Balanced Accuracy (BAL ACC), Brier score, MCC, EF_1pct, EF_5pct, and $\text {BEDROC}_{20/50/80}$. The three model families are evaluated on cross-validated hold-out folds and on the fixed test split

#### Dimensionality reduction with PCA

**Fig. 8:**
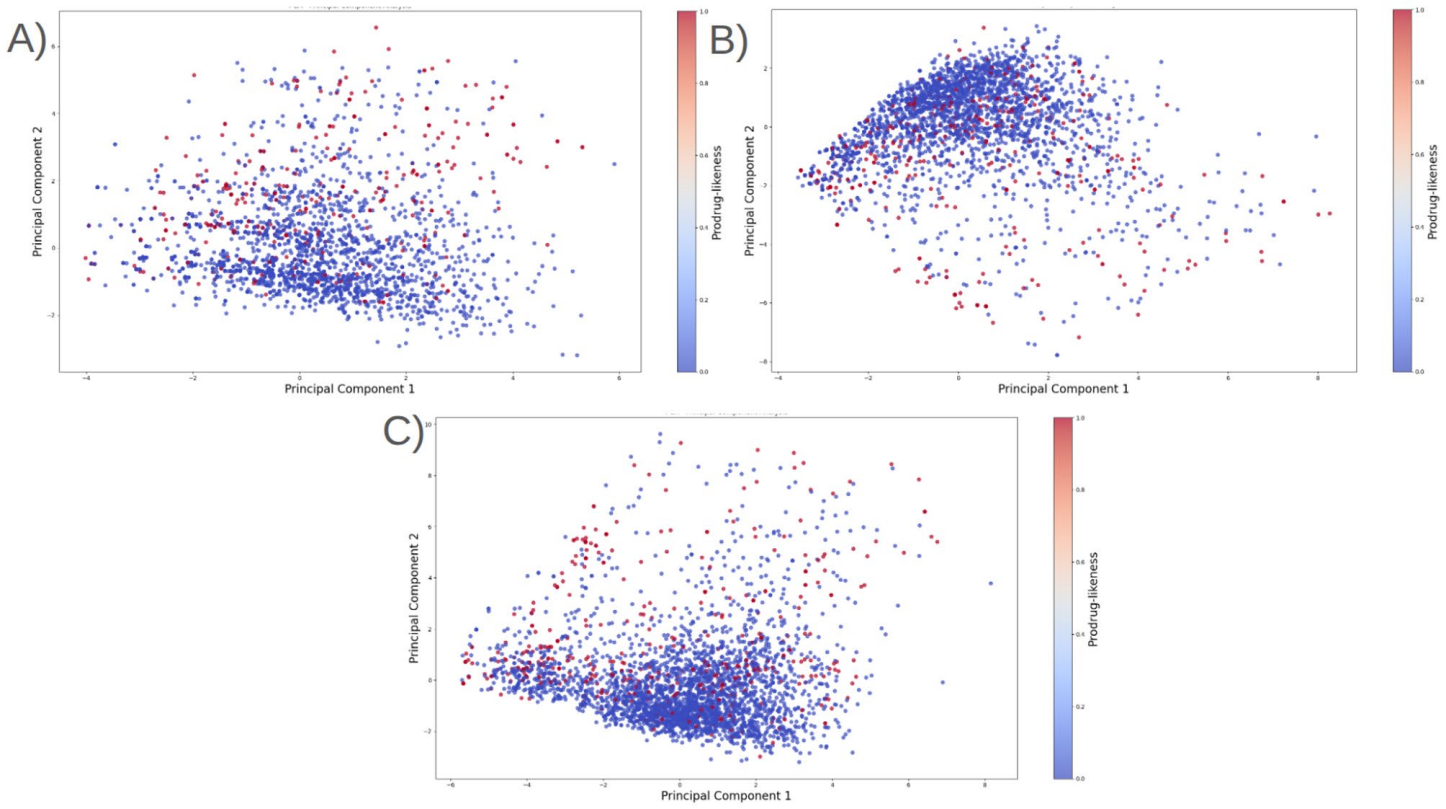
PCA projections (PC1 vs. PC2) for *List_1*** A**, *List_2*** B**, and the concatenated view *List_1+List_2*** C**; points are colored by the Prodrug-ML prodrug-likeness score scaled to [0, 1] (blue = lower score, red = higher score). Each panel presents a two-dimensional PCA embedding of the corresponding standardized feature set, with the first principal component (PC1) on the x-axis and the second principal component (PC2) on the y-axis. Panels A and B show the single-view feature spaces (List_1 and List_2), whereas Panel C shows the early-fusion representation formed by concatenating List_1 and List_2 (List_1+List_2). The color bar encodes the model-predicted prodrug-likeness score, enabling visual assessment of how higher-scoring molecules (warmer colors) are distributed across the chemical feature space. Across all views, high scores are broadly dispersed rather than concentrated in a single compact region. The fused view (Panel C) spans a wider range along the principal directions, consistent with additional variance captured when the two views are combined

Figure 8 illustrates the two-dimensional PCA projections of the Avalon fingerprint feature space for (A) *List_1*, (B) *List_2*, and (C) the early-fused combination (*List_1+List_2*). Each point corresponds to a molecule, color-coded by its predicted prodrug-likeness score (blue: low; red: high). These visualizations provide an unsupervised overview of how each feature representation captures structural variance and distributes prodrug-likeness across chemical space.

Panels A and B reveal distinct structural manifolds. *List_1* shows a more compact and isotropic distribution with moderate overlap between low and high-scoring compounds, indicating that its selected features capture a constrained yet chemically coherent subspace. In contrast, *List_2* spans a broader chemical subspace, with a clearer gradient along the principal components: molecules with higher prodrug-likeness scores concentrate toward the upper and outer regions of the manifold. This suggests that *List_2* contains features that align more directly with the model’s predictive direction, consistent with its stronger early enrichment in single-view analyses.

The fused view in Panel C (*List_1+List_2*) integrates both manifolds, expanding the variance along the principal axes and enhancing the overall score gradient. Regions that were ambiguous in either single view now show a denser distribution of high-scoring compounds, indicating that combining features introduces complementary chemical information. However, the elongated structure and directional gradient visible in the fused embedding also highlight the emergence of mild domain clustering—consistent with the domain-bias analysis that cautions against early fusion. Thus, while early fusion broadens coverage, it also increases the risk of domain-dependent correlations.

In summary, the PCA projections collectively demonstrate that (i) *List_2* offers a more discriminative single-view representation, (ii) the fused *List_1+List_2* view spans a wider chemical space and strengthens score gradients, and (iii) Prodrug-ML’s late-fusion strategy effectively leverages complementary information from multiple feature subsets while minimizing domain leakage that can arise from naive feature concatenation.

#### Non-linear feature projection using t-SNE

**Fig. 9:**
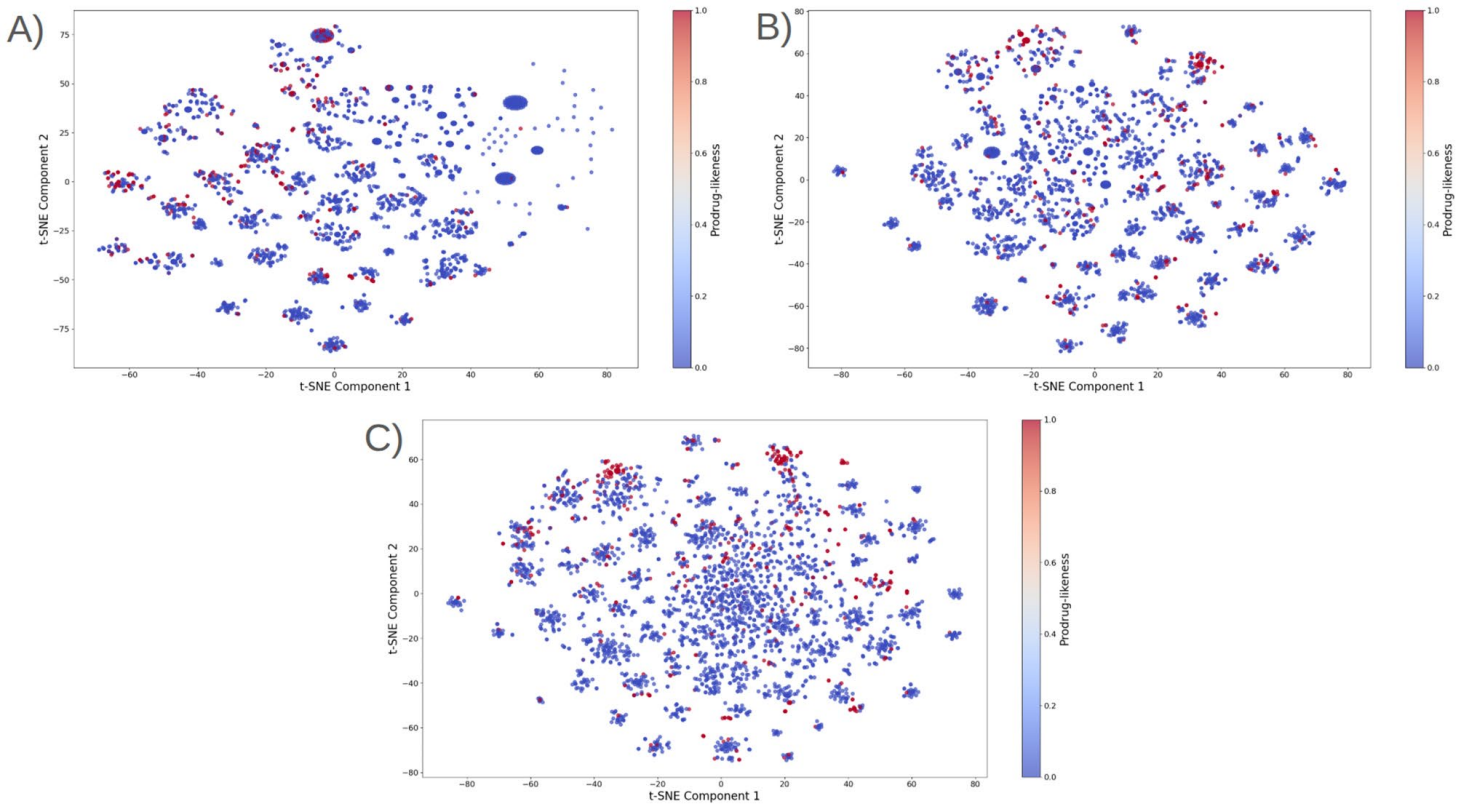
t-SNE embeddings (2D) for *List_1*** A**, *List_2*** B**, and the concatenated view *List_1+List_2*** C**; points are colored by the Prodrug-ML prodrug-likeness score scaled to [0, 1] (blue = lower, red = higher). Note: Each panel shows a two-dimensional t-SNE projection of the corresponding standardized feature set. t-SNE preserves local neighborhoods (axes have no direct physical meaning), allowing visualization of cluster structure in chemical feature space. Panels A and B display the single-view manifolds for *List_1* and *List_2*; Panel C shows the early-fusion manifold for *List_1+List_2*. The color bar encodes the model’s prodrug-likeness label (1 or 0). In A, molecules form many compact micro-clusters with scattered high-score pockets. In B, neighborhood structure is tighter, and high-score data points are more clearly delineated across several regions. In C, combining views yields broader, more continuous coverage with high-score points distributed across multiple neighborhoods, consistent with complementary information from the two views. Overall, high prodrug-likeness is not confined to a single island but appears across several locally coherent regions in the embedded space

Figure 9, Panel A (*List_1*) reveals a fragmented manifold composed of many small, loosely connected neighborhoods. High prodrug-likeness scores (warmer points) appear as scattered pockets at the fringes of several micro-clusters, while the dense cores are predominantly low-to-mid scoring. This pattern suggests that *List_1* contains useful cues, but they are distributed sparsely across local regions rather than aligned along a single dominant direction.

Figure 9, Panel B (*List_2*) shows tighter local structure with clearer, repeated high-score pockets across multiple neighborhoods. The concentration of warmer points along several arcs and cluster boundaries indicates that *List_2* features produce a manifold where prodrug-likeness varies more coherently within local neighborhoods, consistent with its stronger single-view early-enrichment behavior.

Figure 9, Panel C (*List_1+List_2*) yields a broader, more continuous embedding that bridges some of the gaps seen in A and B. High-scoring points are distributed across multiple connected regions rather than confined to isolated islands, suggesting complementary information from the two views: areas ambiguous in either single view become better resolved in the fused space.

Overall, the t-SNE patterns support a multimodel design that provides more coherent local alignment with prodrug-likeness, and combining *List_1* and *List_2* expands coverage and reveals additional high-score neighborhoods.

#### Model interpretability with SHAP

**Fig. 10:**
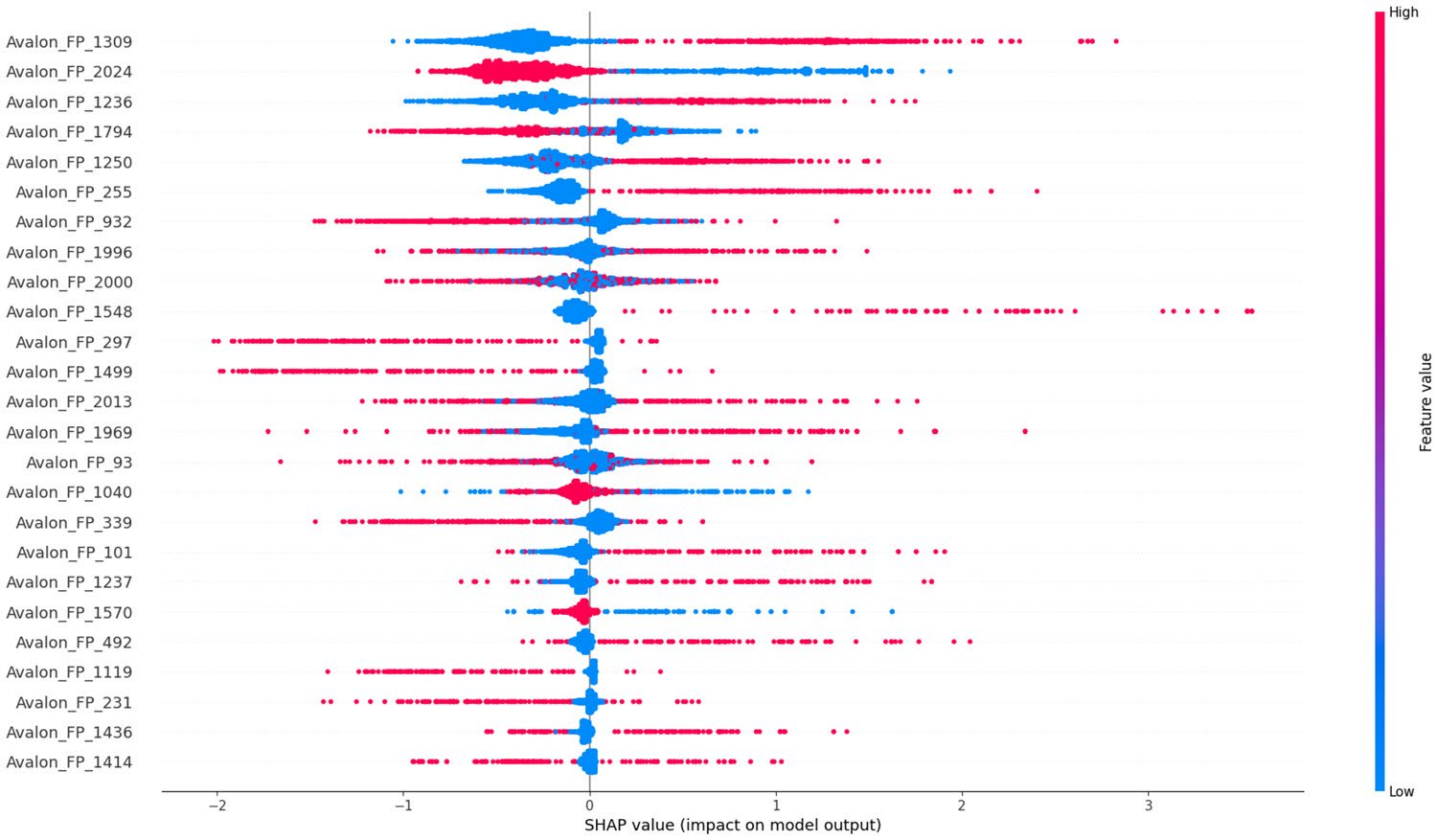
Global SHAP beeswarm for the top Avalon fingerprint features for the List 1, showing per-molecule attributions to the prodrug-likeness score. Each horizontal row corresponds to one Avalon fingerprint bit (feature), ordered by decreasing mean absolute SHAP value (most influential at top). Each dot is a single molecule’s attribution for that feature; dot density reflects the distribution of attributions across the dataset. The x-axis is the SHAP value (feature’s signed impact on the model output): values >0 push the prediction toward “prodrug-like,” values <0 push it toward “non-prodrug”; the vertical line marks zero impact. Dot color encodes the feature value: red = high/“on” (bit present), blue = low/“off” (bit absent); the color bar at right maps this encoding. Axes: y = feature names (Avalon_FP_*), x = SHAP value (impact on model output)

In the beeswarm, several top-ranked Avalon bits (e.g., Avalon_FP_1309, 2024, 1236, 1794, 1250) display dense red point clouds shifted to the right of zero, indicating that the presence of the encoded substructure increases the prodrug-likeness score (Fig. 10). Given that Avalon encodes local atom–bond environments, such right-skewed, “feature-on” patterns can be consistent with oxygen-rich, maskable functionalities and protecting-group–like motifs that commonly appear in carrier-linked prodrugs (e.g., ester/carbonate/carbamate/phosphate neighborhoods or adjacent heteroatom contexts).

Other high-influence bits (e.g., Avalon_FP_255, 932, 1996, 2000, 1548, 297) (Fig. 10) show predominantly red points shifted left of zero, indicating that the presence of the encoded environment reduces the prodrug-likeness score. Such patterns align with substructures that are typically *unmasked* or that lack obvious bioreversible linkages—for example, exposed acid/phenol/primary-amine environments, rigid aromatic neighborhoods with few heteroatom linkages, or topologies less compatible with common promoieties. In practice, these left-skewed, “feature-on” signals suggest that directly presenting strongly polar groups without a cleavable handle, or relying on unmodified heteroaromatic motifs, tends to move candidates away from prodrug-like classification; correspondingly, introducing a suitable bioreversible mask at these positions would be a rational modification to test.

Across the plot for the List_1 (Fig. 10), three behaviors are evident: (i) *positively aligned* rows where red points accumulate at SHAP > 0 (presence favors prodrug-likeness); (ii) *negatively aligned* rows with red points at SHAP < 0 (presence disfavors prodrug-likeness); and (iii) *near-neutral* rows (e.g., mid-ranked bits such as Avalon_FP_93, 1040, 339) where attributions cluster around zero or mix symmetrically, indicating limited or context-dependent influence. Because Avalon uses hashed atom–bond environments, direct one-to-one substructure names are not encoded in the bit labels; nonetheless, the molecule-level SHAP directionality can provide actionable guidance: features whose “on” state consistently increases the score correspond to neighborhoods worth installing as cleavable masks, whereas features whose “on” state consistently decreases the score flag sites where bioreversible protection may be beneficial.

**Fig. 11:**
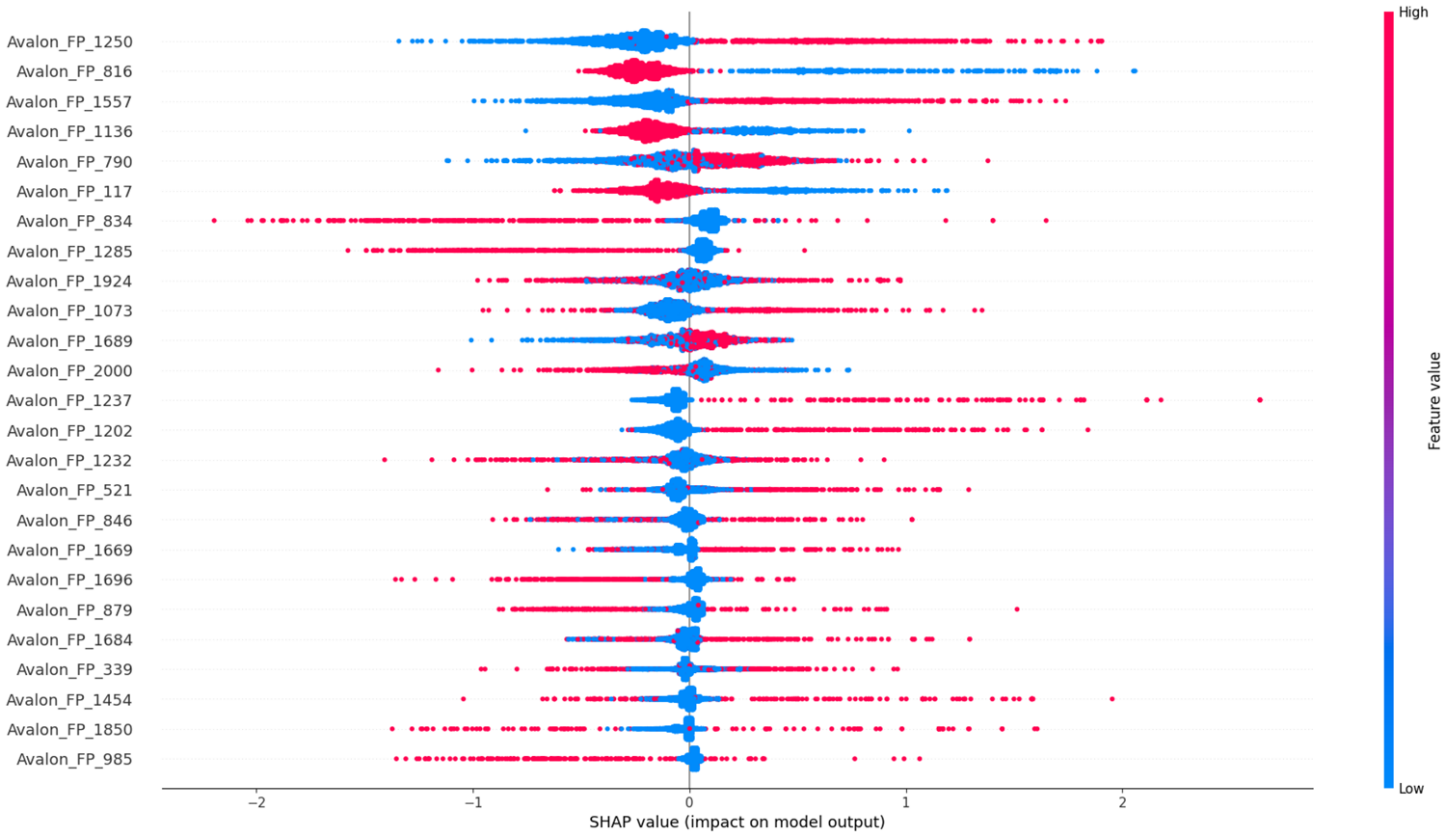
SHAP dependence–swarm summary for the top Avalon fingerprint bits in List_2, visualizing sample-wise attributions and their dependence on feature values for the deployed Prodrug-ML classifier. Each horizontal row corresponds to one Avalon bit from List_2, ordered top-to-bottom by mean absolute SHAP magnitude (most influential first). Every dot is a single molecule’s attribution for that feature; horizontal position gives the SHAP value (signed contribution to the model output), with the vertical jitter showing the distribution of attributions across molecules. The central vertical line marks zero contribution. Point colors encode the underlying feature value (blue = low, red = high), with the colorbar at right indicating the scale. The x-axis is “SHAP value (impact on model output),” and feature names appear on the y-axis. The figure aggregates attributions to provide a compact view of which List_2 bits most affect the prediction and how their values modulate that effect, without implying a one-to-one substructure identity for any hashed bit

As for the SHAP results for the List_2, several high-impact bits in Fig. 11 (e.g., Avalon_FP_1250, ..._816, ..._1557, ..._1136, ..._790) show red points (high bit value) clustered to the right of zero SHAP, while blue points (low/absent) lie nearer or left of zero. This pattern indicates that the presence of the corresponding hashed substructures increases the prodrug-likeness score. Although Avalon bits are hashed and many-to-one with respect to exact chemotypes (Fig. 11), the directionality is consistent with fragments often enriched in prodrug designs—e.g., heteroatom-rich or masked-functionality motifs (acyl/alkoxy patterns, incipient ester/carbonate/carbamate-like environments), and scaffolds compatible with installing cleavable linkers. Practically, candidate enumeration that preserves or introduces such “positively weighted” fragments tends to move molecules into higher-ranked bins under Prodrug-ML.

Other influential bits (e.g., Avalon_FP_834, ..._1285, ..._2000, ..._1237, ..._1202, ..._1232 on Fig. 11) display the opposite signature: red points lie predominantly at negative SHAP values, with blue points nearer or to the right of zero. Here, the presence of the hashed substructure depresses the prodrug-likeness score. While the hash prevents a unique structural decode, the observed pattern is compatible with features that resist derivatization or are less typical of classical prodrug motifs—e.g., highly rigid/aromatic fragments with limited handle density, strongly hydrophobic patterns lacking nucleophilic/heteroatom sites, or substructures suggestive of non-labile linkages. In a design loop, deprioritizing candidates that accumulate several such negatively associated bits—or modifying them to introduce accessible functional handles—can improve the likelihood of prioritization.

A subset of List_2 bits (Fig. 11) shows mixed clouds with red and blue points straddling zero SHAP, indicating context-dependent or near-neutral contributions. These features neither consistently raise nor depress the score and should not drive design decisions in isolation. Overall, the List_2 SHAP profile separates (i) positively associated fragments that tend to elevate prodrug-likeness, (ii) negatively associated fragments that tend to lower it, and (iii) neutral/mixed bits with weak or context-specific influence. While individual Avalon bits are not directly interpretable as single named substructures, their signed contributions provide actionable guidance: emphasize candidates that carry multiple positively weighted fragments and viable attachment/cleavage environments; minimize or remodel motifs repeatedly flagged with negative attributions; and treat mixed-effect bits as secondary to project-specific SAR.

In summary, the SHAP analyses (Fig. 10, and 11) translate hashed fingerprint signals into chemically actionable guidance: bits whose “on” state consistently yields positive attributions highlight neighborhoods compatible with maskable functionalities and cleavable linkers (e.g., ester/carbonate/carbamate/phosphate-like contexts), suggesting prioritization of designs that temporarily protect polar handles; conversely, bits with consistently negative attributions flag scaffolds or local environments that are difficult to derivatize or atypical of classical prodrug motifs, indicating candidates for deprioritization or for introduction of accessible attachment sites. While Avalon bits are many-to-one encodings, the signed directionality enables practical interventions for medicinal chemistry: enrich enumerations in positively weighted fragments, remodel or mask negatively weighted environments, and treat mixed/near-neutral bits as context dependent.

#### Feature importance analysis (ANOVA F-test)

**Fig. 12:**
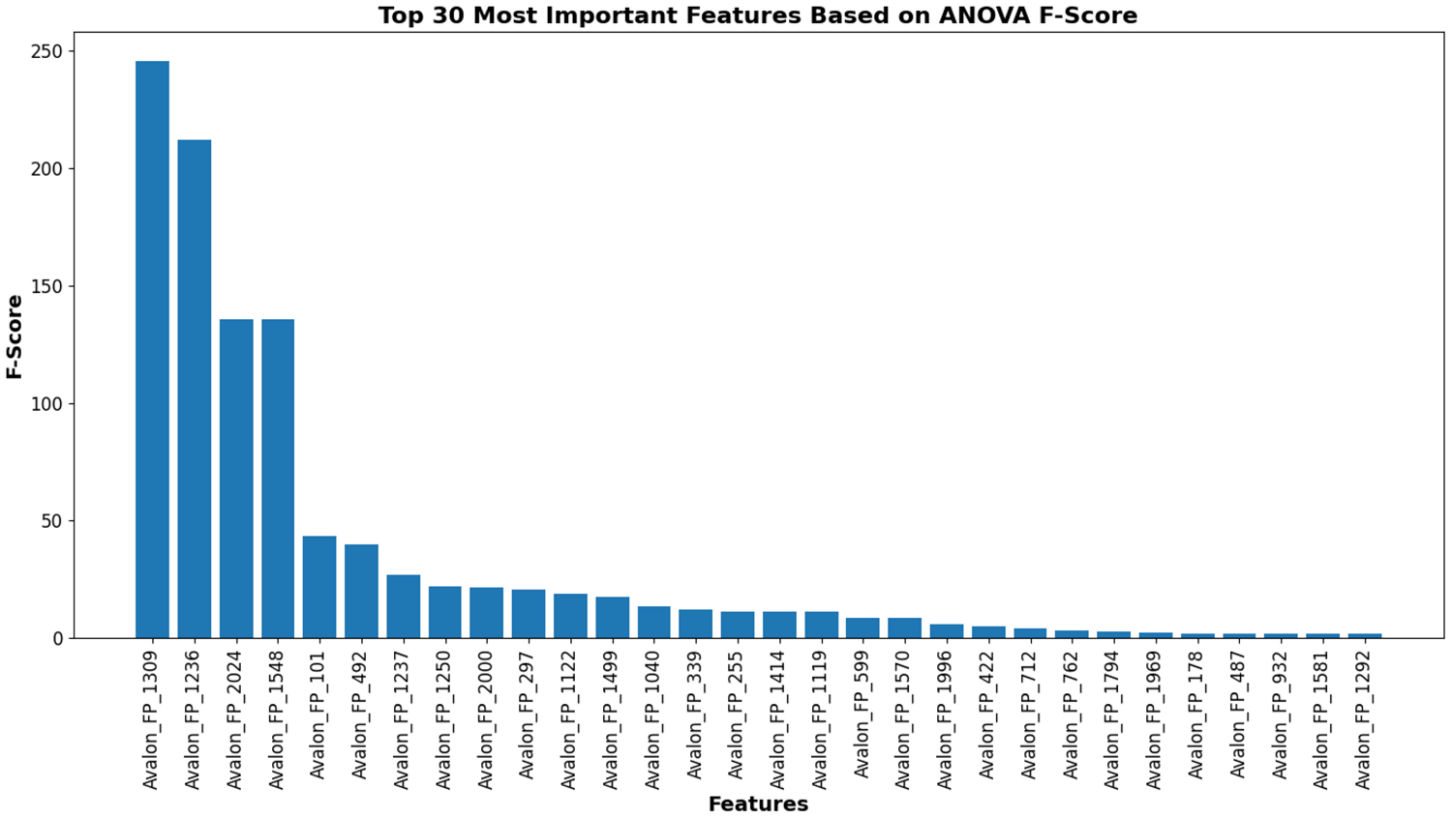
Top 30 discriminative features in *List_1* ranked by one-way ANOVA F-score (higher values indicate stronger class separation). The bar chart reports the ANOVA F-score computed independently for each *List_1* feature (Avalon fingerprint bits), quantifying the ratio of between-class to within-class variance for the prodrug-likeness labels. The x-axis lists the feature identifiers (e.g., Avalon_FP_1309, Avalon_FP_1236, Avalon_FP_2024), ordered from most to least discriminative; the y-axis gives the corresponding F-scores. A few features dominate—Avalon_FP_1309 and Avalon_FP_1236 have the largest F-scores, followed by a steep drop and a long tail of progressively smaller effects. This heavy-tailed profile indicates that a compact subset of *List_1* bits carries most of the label-separating signal, motivating the feature-selection step used in Prodrug-ML

The ANOVA screen for *List_1* (Fig. 12) exhibits a pronounced “elbow” in importance: a single dominant bit, Avalon_FP_1309, attains an F-score of roughly $F\!\approx \!245$, followed by Avalon_FP_1236 at $\approx \!212$; the next tier drops sharply to $\approx \!135$ for Avalon_FP_2024 and $\approx \!135$ for Avalon_FP_1548 (Fig. 12). Below this elbow, effects taper quickly into a long tail—e.g., Avalon_FP_101 and Avalon_FP_492 around $F\!\approx \!40$, a mid-pack of bits in the $F\!\approx \!10{-}25$ range (such as Avalon_FP_1237, Avalon_FP_1250, Avalon_FP_2000, Avalon_FP_297, Avalon_FP_1122), and many features below $F\!\approx \!10$ (e.g., Avalon_FP_599, Avalon_FP_1570, Avalon_FP_1996, Avalon_FP_712, Avalon_FP_762, Avalon_FP_1794, Avalon_FP_1969, Avalon_FP_178, Avalon_FP_487, Avalon_FP_932, Avalon_FP_1581, Avalon_FP_1292) (Fig. 12). This heavy-tailed profile indicates that a compact subset of *List_1* bits explains most of the between-class variance, while the majority contribute marginal univariate separation. Practically, these numbers justify aggressive, view-specific feature selection (to capture the high-*F* signals and reduce noise/collinearity) and help explain why a multimodel, late-fusion strategy can improve early enrichment: strongly discriminative bits from *List_1* are preserved, whereas weak, redundant bits are down-weighted by the ensemble.

**Fig. 13:**
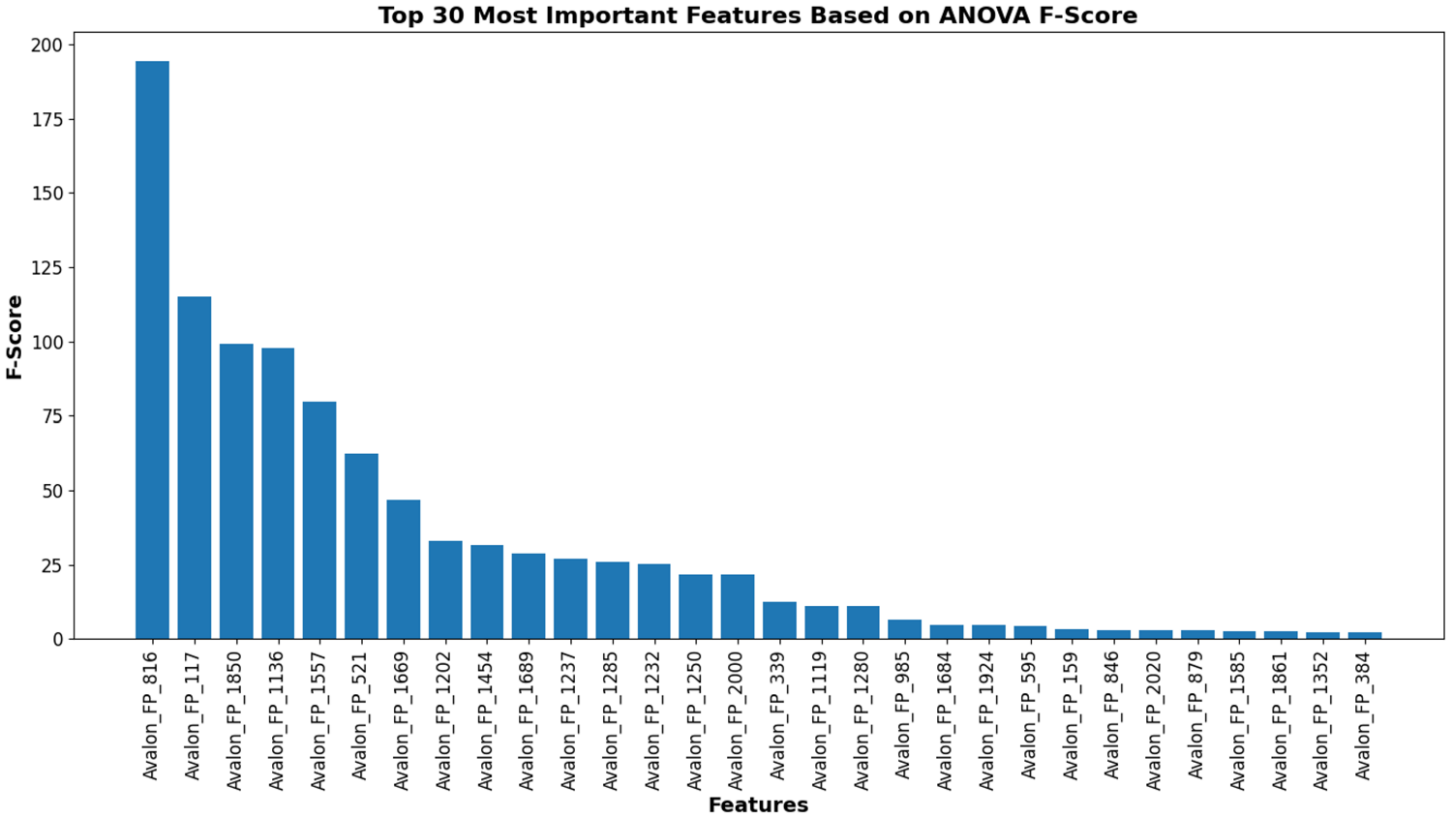
Top 30 discriminative features in *List_2* ranked by one-way ANOVA F-score (higher values indicate stronger class separation). The bar chart reports univariate ANOVA F-scores computed independently for each *List_2* descriptor (Avalon fingerprint bits) with respect to the prodrug-likeness labels. The x-axis lists feature identifiers (e.g., Avalon_FP_816, Avalon_FP_117, Avalon_FP_1850), ordered from most to least discriminative; the y-axis shows the corresponding F-score. Thus, the tallest bars correspond to the features that, by themselves, produce the largest between-class/within-class variance ratio (for example, Avalon_FP_816 has the highest score, followed by Avalon_FP_117 and Avalon_FP_1850), while the rightmost bars represent progressively smaller effects. The figure summarizes which *List_2* bits contribute the strongest univariate separation used in the view-specific feature selection step

The *List_2* ANOVA profile (Fig. 13) shows a clear head–tail structure with several strongly discriminative bits followed by a long taper. The top feature, Avalon_FP_816, reaches an F-score of $\sim \!195$, followed by Avalon_FP_117 at $\sim \!115$, Avalon_FP_1850 at $\sim \!100$, and Avalon_FP_1136 at $\sim \!98$ (Fig. 13). A second tier spans $\sim \!80{-}60$ for Avalon_FP_1557 ($\sim \!80$) and Avalon_FP_521 ($\sim \!62$), then drops to $\sim \!47$ for Avalon_FP_1669. Mid-range effects populate the $\sim \!33{-}21$ band (Avalon_FP_1202 $\sim \!33$, Avalon_FP_1454 $\sim \!30$, Avalon_FP_1689 $\sim \!29$, Avalon_FP_1237 $\sim \!27$, Avalon_FP_1285 $\sim \!26$, Avalon_FP_1232 $\sim \!24$, Avalon_FP_1250 $\sim \!22$, Avalon_FP_2000 $\sim \!21$, Avalon_FP_339 $\sim \!19$), and the tail falls below $\sim \!13$ (Avalon_FP_1119 $\sim \!13$, Avalon_FP_1280 $\sim \!12$, Avalon_FP_985 $\sim \!10$, down to $\sim \!1{-}3$ for bits like Avalon_FP_2020, Avalon_FP_1585, Avalon_FP_1861, Avalon_FP_1352, Avalon_FP_384). Compared with *List_1*, the high end is slightly less extreme, but there are more mid-strength contributors, implying that *List_2* spreads signal across a broader set of bits—consistent with its stronger cross-decoy transfer (Fig. 13). Practically, these numbers justify selecting a compact top slice (e.g., the first $20{-}30$ bits) while down-weighting the long tail, which reduces noise and helps Prodrug-ML retain the most label-informative structure from *List_2*.

In summary, the ANOVA F-test reveals a sparse–heavy–tailed structure of predictive signal in both views: *List_1* is dominated by a few very strong bits (e.g., Avalon_FP_1309 $\sim \!245$, Avalon_FP_1236 $\sim \!212$) followed by a steep falloff, whereas *List_2* distributes signal across a broader set with multiple mid-strength contributors ($\sim \!115{-}60$). Such a pattern validates the design choice of view-specific feature selection plus late fusion—retain the high-F slice from each view and down-weight the long tail—thereby reducing noise and curbing domain-specific artifacts that appear when all 2048 bits are used. The complementary importance profiles of *List_1* and *List_2* explain why a two-view ensemble is effective: each view contributes distinct high-value bits, and their combination yields the early-retrieval and BEDROC gains observed elsewhere. Overall, the feature-importance analysis supports Prodrug-ML’s multimodel ensemble feature-selection strategy as a principled driver of its robust early enrichment and stable generalization.

#### Summary of Prodrug-ML analysis

The comprehensive analyses conducted for Prodrug-ML—spanning both main-text and supplementary investigations—jointly validate the robustness, interpretability, and chemical plausibility of the framework. Together, these complementary approaches demonstrate that the observed predictive performance is not a byproduct of data leakage, random correlations, or overfitting, but arises from meaningful, transferable structure–activity relationships learned from the data.

In the main-text analyses, five orthogonal validation strategies were employed to validate and investigate the efficiency of Prodrug-ML.

1. *Label Randomness Analysis* confirmed that the pipeline behaves correctly under null conditions: when class labels are shuffled, all metrics (ROC AUC, EF, BEDROC, AP) collapse to chance levels, ensuring that true performance gains originate from genuine signal rather than artifacts or leakage (Table 7).
2. *Dimensionality Reduction with PCA* revealed that individual feature subsets (*List_1*, *List_2*) occupy distinct yet overlapping manifolds, each contributing complementary chemical variance, while the fused view (*List_1+List_2*) expands this space but introduces mild domain clustering—supporting the late-fusion design choice (Fig. 8).
3. *Non-Linear Feature Projection with t-SNE* visualized richer, locally coherent structures where high prodrug-likeness regions appear across multiple manifolds, indicating that the model captures transferable cues spread across diverse chemical neighborhoods (Fig. 9).
4. *Model Interpretability with SHAP* translated hashed Avalon features into chemically meaningful attributions: fragments positively associated with high prodrug-likeness correspond to known bioreversible motifs (e.g., ester, carbonate, carbamate, phosphate environments), while negatively weighted fragments align with unmasked or rigid aromatic scaffolds, offering actionable design guidance (Figs. 10, and 11).
5. *Feature Importance Analysis (ANOVA F-test)* revealed a heavy-tailed distribution of discriminative power—few features with high F-scores followed by many weak contributors—justifying the feature-selection and late-fusion strategy that aggregates complementary signals while reducing redundancy and noise (Fig. 12).

In the supplementary analyses, extended diagnostics strengthened these conclusions.

1. *Correlation Heatmaps* demonstrated low pairwise inter-feature correlations and diffuse bit–label associations, confirming minimal multicollinearity and validating that predictive signal emerges from combining many weakly correlated descriptors.
2. *Hierarchical Feature Clustering (Dendrogram)* revealed modular feature organization with clear cluster boundaries and small label-proximal modules anchored by key informative bits, further supporting the use of representative sampling and view-specific selection.
3. *Multivariate Feature Visualization with RadViz* showed that both views form dense, overlapping manifolds with distributed signal, underscoring the necessity of non-linear ensemble learners and confirming that Prodrug-ML’s architecture effectively amplifies subtle, multivariate cues.

Taken together, these findings demonstrate that Prodrug-ML satisfies three critical conditions for methodological soundness: (i) *Validity*—performance arises from true signal, verified through label-randomness and cross-decoy validation; (ii) *Robustness*—consistency across complementary analyses (PCA, t-SNE, ANOVA, SHAP, and correlation structure) shows stability and generalization to unseen chemistry; and (iii) *Chemical interpretability*—key model features correspond to realistic, bioreversible substructures and transferable medicinal-chemistry principles.

In conclusion, the integrated main and supplementary analyses collectively support that Prodrug-ML is a scientifically reliable and chemically meaningful framework for early prodrug screening. Its multimodel, late-fusion design successfully balances interpretability and generalization, capturing diffuse yet authentic prodrug-relevant signals while avoiding domain bias, overfitting, and redundancy. These convergent results position Prodrug-ML as a robust foundation for future extensions in data-driven prodrug discovery.

### Case study: classifier choice, fragment-level signals, and prodrug-oriented chemical suggestions

Table 8 lists *test* compounds with their fragment tags and LightGBM scores (Proba_pos). Negatives are predominantly scored near zero (e.g., 0.000-$-$0.003 for amide/sulfonamide/piperidine/piperazine/tertiary_amine motifs), including representative entries such as CCCn1cc(S(=O)(=O)N2...)=0.000, Cc1[nH]c(C)c(S(=O)(=O)N...)=0.000, and O=S(=O)(c1ccc(Cl)cc1)N1CCC...=0.003. By contrast, positives span mid to extremely high probabilities, with several high-confidence prodrugs concentrated at the top of the list (e.g., 0.962, 0.978, 0.988) (Table 8). The scores indicate that Prodrug-ML is a promising framework to screen prodrugs.

**Table 8:** Case-study compounds with their SMILES strings, binary labels, dataset sources, detected target-fragment tags, and Prodrug-ML (default classifier=LightGBM, and linearly weighted base models (1:1)) predicted positive-class probabilities

Smiles	Label	Source_dataset	Target_fragments	Proba_pos
CCCn1cc(S(=O)(=O)N2CCCC[C@@H]2N=C=O)nc1C	0	dude	piperidine;sulfonamide;tertiary_amine	0.000
Cc1[nH]c(C)c(S(=O)(=O)N(Cc2ccccc2)C(C)C)c1C(=O)N1CCCC1	0	chembl_high_conf	amide;sulfonamide;tertiary_amine	0.000
Cc1cccn2c(CN(C)C3CCOC3)c(C(=O)N3CCCCC3)nc12	0	chembl_high_conf	amide;piperidine;tertiary_amine	0.000
Cc1ccc2c(c1)nc(N1CCN(Cc3ccc(F)cc3)CC1)c1cccn12	0	chembl_high_conf	aryl_halide;piperazine;tertiary_amine	0.001
O=C(CCCNC(=O)NC1CCCCC1)Nc1ccc2c(c1)CCC(=O)N2Cc1ccc(Cl)cc1	0	dude	amide;aryl_halide;tertiary_amine;urea	0.001
CCc1cc(-c2ccccc2)cc(CC)[n+]1CC(=O)Nc1ccc(S(=O)(=O)/N=c2/sc(S(N)(=O)=O)nn2C)cc1	0	chembl_random	amide;sulfonamide;tertiary_amine	0.001
CCCCCCCCc1ccc(OCC(=O)Cn2cc(C(=O)O)c3ccccc32)cc1	0	chembl_random		0.002
O=C([C@H]1CCCN1C(=O)c1cccc(C(=O)N2CCC[C@H]2C(=O)N2CCCCCC2)c1)N1CCCC1	0	chembl_high_conf	amide;tertiary_amine	0.002
Cc1nc2nccc-2c(NCCc2cccc(N(C)C)c2)[nH]1	0	chembl_high_conf	tertiary_amine	0.002
Cc1cc([C@](C)(O)CNC(=O)C(=O)Nc2ccc3c(c2)COC3)c(C)o1	0	dude	amide;tertiary_amine	0.002
O=S(=O)(c1ccc(Cl)cc1)N1CCCc2cc(Cl)c(Oc3cc(-c4nc(C5CC5)no4)cc(Cl)n3)cc21	0	chembl_random	aryl_halide;sulfonamide;tertiary_amine	0.003
O=S(=O)(Nc1ncc(F)s1)c1cc(Cl)c(NCC23CCCN2CCC3)cc1F	0	chembl_high_conf	aryl_halide;sulfonamide;tertiary_amine	0.005
CCOc1ccc([C@H](c2sc3nc(C)nn3c2[O-])N2CCN(CCO)CC2)cc1OC	0	dude	piperazine;tertiary_amine	0.006
Nc1nc(O)cc(Nc2ccc(F)c(F)c2)n1	0	chembl_high_conf	aryl_halide;tertiary_amine	0.007
CCC(CC)Nc1cc(C)nc2c(-c3ccc(OC)cc3C)c(C)nn12	0	chembl_high_conf	tertiary_amine	0.007
CC1(C)OC(C)(C)[C@@H](N)[C@H]1CN1CCN(S(C)(=O)=O)CC1	0	dude	piperazine;sulfonamide;tertiary_amine	0.013
O=C1c2cc(S(=O)(=O)N(CCO)CCO)ccc2-c2ccc(S(=O)(=O)N(CCO)CCO)cc21	0	dude	sulfonamide;tertiary_amine	0.013
CCc1cccc(Nc2ncc3cc(-c4c(Cl)cccc4Cl)c(=O)n(C)c3n2)c1	0	chembl_random	aryl_halide;tertiary_amine	0.013
CCCc1cc(-c2cccc(Cl)c2)nc(C#N)n1	0	chembl_high_conf	aryl_halide	0.020
CCN(C(=O)c1ccc(C)cc1)c1cc(-c2ccccc2)sc1C(=O)O	0	chembl_random	amide;tertiary_amine	0.027
CN(C)CCn1c(=O)oc2ccc(NC(=O)CSc3nncs3)cc21	0	dude	amide;tertiary_amine	0.036
CC(C)(C)c1ccc([C@@H](O)Cc2ccc(Br)s2)cc1	0	dude	aryl_halide	0.062
CC(C)(O)c1ccc2c(c1)c1cc(Cl)ccc1c1nc(-c3c(C#N)cccc3C#N)[nH]c21	0	chembl_random	aryl_halide	0.062
NN[C@@H](CSc1cccc(F)c1)c1ccc[nH+]c1N	0	dude	aryl_halide	0.130
O=C(c1ccccc1F)N1CCC2(CC1)CC(Oc1ccccn1)CO2	0	chembl_high_conf	amide;aryl_halide;piperidine;tertiary_amine	0.225
CN(C)c1ccc2nc3ccc(N)cc3[s+]c2c1	0	chembl_random	tertiary_amine	0.271
CCN(CC)CC(=O)Oc1ccc(cc1)NC(C)=O	1	prodrug	amide;tertiary_amine	0.275
B(C1=CC=C(C=C1)C(=C(CC)C2=CC=CC=C2)C3=CC=C(C=C3)OCCN(C)C)(O)O	1	prodrug	tertiary_amine	0.342
C1=C(C(=CC(=C1N(CCCl)CCCl)[N+](=O)[O-])[N+](=O)[O-])C(=O)N	1	prodrug	amide;tertiary_amine	0.465
C1CCCN(c2nc(C3CC3)nc3c2CCCCN3)CC1	0	chembl_random	tertiary_amine	0.523
C1=CC=C(C=C1)C[Se]CC(C(=O)O)N	1	prodrug		0.627
CC(C)C[NH2+]CC1(N)CC1	0	dude		0.629
CC(C)(C)c1ccc(CCNC(=O)c2ccccc2)cc1	0	chembl_high_conf	amide;tertiary_amine	0.761
O=C(COc1ccc2ccccc2c1)NCCCCNC(=O)COc1ccc2ccccc2c1	0	dude	amide;tertiary_amine	0.761
CCCC(=O)OCOCC1OC(CS1)N2C=CC(=NC2=O)NC(=O)C	1	prodrug	amide;tertiary_amine	0.786
COC(=O)CCC(=O)CN	1	prodrug		0.834
CCCCCCCCCCCCC(CC)C(=O)Nc1c(OC)cc(OC)cc1OC	0	chembl_random	amide;tertiary_amine	0.871
CC(=O)OC1CCC2C3CCC4(C(C3CCC2=C1)CCC4(C#C)OC(=O)C)C	1	prodrug		0.962
CC12CCC3C(C1CC(C2(C#C)O)O)CCC4=C3C=CC(=C4)OC5CCCC5	1	prodrug		0.978
CCCC(=O)OC1(CCC2C1(CCC3C2CCC4=CC(=O)CCC34)CC)C#C	1	prodrug		0.988

The table lists one row per selected compound from the case-study set. “Smiles” provides the canonical string used in modeling (stereochemistry and charge notation shown verbatim). “Label” is the ground-truth class used in evaluation (0 = non-prodrug, 1 = prodrug). “Source_dataset” indicates the molecule’s origin in the curated test pool (e.g., *chembl_high_conf*, *chembl_random*, *dude*, *prodrug*). “Target_fragments” contains semicolon-separated fragment tags assigned by predefined SMARTS rules (e.g., piperidine, sulfonamide). “Proba_pos” is the LightGBM-predicted probability in [0, 1] for the positive (prodrug-like) class computed on the fixed test set. Duplicate SMILES may appear if retained as separate entries in the source files; values are reported exactly as produced by the pipeline without additional normalization

The strongest prodrug-like signals coincide with canonical, cleavable masks on suitable handles. For example, three steroidal/esterified-looking entries score 0.962, 0.978, and 0.988 (all label = 1) despite lacking explicit fragment tags in this view, consistent with bulky, lipophilic promoieties or simple ester/carbonate masks on alcohol/phenol handles. Additional positives with explicit tags include 0.786 and 0.834 (label = 1; both list amide/tertiary_amine) (Table 8), again compatible with masked or maskable functionalities (e.g., esters/carbonates/carbamates) that are widely used in marketed prodrugs.

A small subset of negatives receive elevated scores (e.g., 0.523, 0.629, 0.761, 0.761, 0.871; all label = 0), and they are enriched for amide/tertiary_amine (sometimes aryl_halide) tags (Table 8). These discordant cases typically resemble prodrug scaffolds in polarity/lipophilicity balance but lack an explicit cleavable trigger in the current representation; they are prime candidates for introducing a *designed* mask. Conversely, several positives appear with only moderate scores (e.g., 0.275, 0.342, 0.465, 0.627; all label = 1), often reflecting smaller or less overt promoieties; these entries illustrate how fragment context and mask placement modulate the model’s confidence.

Table 8 shows that a pragmatic rule emerges: when fragment tags indicate many non-cleavable polar motifs (e.g., repeated amide/sulfonamide with tertiary_amine), elevating prodrug-likeness generally requires *adding a cleavable mask on an available OH or NH* (Table 8). In practice, the concrete moves suggested by the distribution in Table 8 are: (i) ester or carbonate masks on alcohol/phenol handles to enable esterase-triggered release; (ii) carbamate or amide masking of primary/secondary amines to moderate basicity and improve passive diffusion, with enzymatic unmasking restoring the parent; (iii) phosphate/phosphonate esters to boost solubility for neutral lipophilic parents; (iv) acyloxymethyl/self-immolative linkers to convert a single enzymatic event into clean payload release; and (v) amino acid/peptide promoieties to exploit transporter uptake. The high scores near 0.96-$-$0.99 (label = 1) exemplify the end state of such masking, while the elevated-but-negative cases (e.g., 0.629 or 0.871 with amide/tertiary_amine) are the clearest opportunities for introducing explicit, enzymatically addressable triggers.

In summary, the case study indicates that the default LightGBM model captures fragment–level regularities that align with established prodrug design heuristics. Low Proba_pos values concentrate among scaffolds rich in non-cleavable polar motifs (e.g., amide/sulfonamide/tertiary-amine), whereas high scores track the presence—or plausibility—of cleavable masks on suitable handles, consistent with the top label = 1 entries in Table 8 (e.g., 0.962-$-$0.988). Borderline and discordant rows (label = 0 with elevated Proba_pos, or label = 1 with only moderate scores) serve as actionable diagnostics: in such cases, introducing an enzymatically addressable promoiety (ester, carbonate/carbamate, phosphate/phosphonate, or a self-immolative linker) on an existing OH/NH offers a chemically grounded route to shift candidates toward prodrug-like space. Overall, the table–anchored patterns support a classifier choice that balances accuracy and interpretability while yielding concrete, fragment-guided suggestions for lead optimization.

### Challenges and future opportunities

Prodrug-ML is the first openly available framework dedicated to prodrug screening, accelerating early discovery by transforming heterogeneous literature curation into a reproducible, bias-aware machine learning pipeline. It addresses the “no true negatives” problem and the attendant risk of domain-class leakage by (i) constructing complementary, property-aware decoy cohorts (e.g., DUD-E–style matched decoys, random and stringently filtered ChEMBL sets) and validating across decoy types to suppress recipe artefacts; (ii) enforcing robust evaluation designs (scaffold/temporal splits, domain-classifier checks) and late-fusion ensembling over view-specific feature selection to balance accuracy with generalization; and (iii) delivering an analysis suite that probes model behavior from multiple angles (feature stability, substructure attributions/SHAP, ablations, early-enrichment diagnostics, and error taxonomy), thereby converting predictions into actionable design hypotheses. These contributions substantially improve screening reliability and transparency in a space historically limited by data incompleteness and hidden shortcuts. However, as a first-generation framework centered on fingerprint-level signals and binary labels, Prodrug-ML still omits explicit mechanism/trigger modeling, species/tissue context, and calibrated per-molecule risk controls, and its positive corpus—while carefully curated—cannot yet capture the full diversity of rare promoieties.

The *smProdrugs* database (2023 release) used in this study represents the most comprehensive and up-to-date collection of experimentally validated prodrugs, containing just over 600 positive entries. While new compounds are periodically added through individual submissions that undergo expert curation and confirmation before public release, the total number of accessible, validated prodrugs remains limited. As a result, nearly all known prodrugs are already represented in this dataset, precluding a separate “unseen” validation cohort at present. Nevertheless, this limitation highlights the urgent need for data-driven acceleration in prodrug research, and frameworks such as *Prodrug-ML* are instrumental in bridging this gap—providing an extensible, bias-controlled foundation that can be directly applied to future database expansions or proprietary libraries for forward, prospective evaluation.

Prodrug-ML is an early decision-support screen that ranks candidates by learned prodrug-likeness; it does not model activation mechanisms, bioconversion rates, or species-dependent metabolism, and its binary label necessarily abstracts away pharmacological nuances (e.g., partial/slow conversion, multi-step triggers). Although the positive set (more than 600 curated prodrugs) covers established chemotypes, it limits the diversity of rare motifs and constrains the model’s applicability domain. The current fingerprint-centric features do not explicitly encode cleavable linkages or site-specific triggers (e.g., ester vs phosphate context, enzyme preferences), and we have not yet incorporated formal uncertainty calibration for per-molecule risk control beyond standard probability calibration. These constraints suggest clear extensions: (i) Mechanism-aware labels and multi-task learning to predict putative activation class (hydrolysis/oxidation/reduction) alongside prodrug-likeness; (ii) motif- and reaction-centric descriptors (bond-editing, attachment-site context, SMARTS-level cleavage signatures) to capture design intent; (iii) applicability-domain quantification and conformal risk control to provide user-set coverage guarantees; (iv) species mapping via enzyme-expression priors and knowledge graphs (e.g., esterases/phosphatases tissue distributions); (v) prospective, blinded screening on internal libraries and chemist-in-the-loop active learning to expand coverage efficiently; and (vi) integration of structure-derived features (docking/MD-informed accessibility, solvent exposure, microenvironment proxies) for designs where 3D context is available. These directions would move Prodrug-ML from triage toward mechanistically annotated prioritization with calibrated uncertainty and broader real-world utility.

Binary framing is a recognized limitation and also a practical springboard for extension: prodrug behavior spans mechanism, conversion extent/rate, and species context rather than a simple yes/no label. A concrete path forward is to augment the corpus with structured annotations—conversion fraction and half-life/time-to-conversion, activation class (e.g., esterase, phosphatase, oxidation, reduction), bond/attachment context, and species/tissue metadata—leveraging fields already present in *smProdrugs* together with targeted literature curation of high-frequency chemotypes; on top of this, a shared encoder can support multi-task heads for (i) prodrug-likeness (classification), (ii) activation class (multi-class), and (iii) conversion extent (ordinal/continuous), with optional survival modeling for time-to-conversion. Mechanism priors can be injected via motif/reaction descriptors (SMARTS-level cleavage signatures, bond-editing features, local microenvironment around labile bonds) and enzyme/tissue priors (e.g., esterase/phosphatase distributions) to capture species differences, while calibrated uncertainty (temperature scaling plus inductive conformal prediction) flags low-confidence or out-of-domain cases. Deployment guidance then becomes actionable: high-confidence, in-domain predictions inform short synthesis lists; low-confidence or mechanism-ambiguous cases are routed to orthogonal follow-ups (e.g., ADMET assays, mechanism-focused docking/MD, or enzyme-panel experiments).

The present representation deliberately employs hashed Avalon fingerprints to maintain a simple, reproducible pipeline centered on the principal methodological bottleneck—constructing reliable negative cohorts and suppressing domain/recipe bias—prior to introducing mechanistic chemistry. Consequently, the model does not explicitly encode prodrug-relevant functional motifs (e.g., esters, carbonates/carbamates, phosphates/phosphonates, azo linkages), cleavage or attachment-site context, or activation mechanisms (hydrolysis/oxidation/reduction). Although domain-bias–aware feature screening surfaces stable substructure signals implicitly, a mechanism-aware descriptor layer remains absent. A staged expansion is therefore planned as a primary avenue for future work: (i) SMARTS-based motif panels for common promoieties and labile bonds; (ii) attachment-site context descriptors (neighbor atom types, local hybridization/charge, ring/branch environment); (iii) reaction-center and bond-edit features derived from parent–prodrug pairs; (iv) enzyme/cleavage priors (e.g., esterase/phosphatase propensity proxies, tissue/expression hints) to reflect species or compartment effects; and (v) graph encoders with attention to localize labile subgraphs, optionally coupled to matched-molecular-pair transforms to model typical prodrugging edits. These additions are intended to be introduced under a pre-registered, ablation-based protocol (fingerprints $\rightarrow$ +motifs $\rightarrow$ +context $\rightarrow$ +enzyme priors $\rightarrow$ +GNN) with cross-decoy validation to ensure that observed gains reflect mechanism-relevant signal rather than renewed domain shortcuts.

Prodrug-ML framework intentionally adopts a minimalist meta-level design—equal-weight late fusion over a compact set of base learners—to keep the learning pipeline transparent and to center the contribution on constructing high-quality negative cohorts for prodrug-likeness. This simplicity, while methodologically clarifying, likely underutilizes complementary inductive biases across models. Future extensions could explore richer ensemble strategies (e.g., stacked generalization with calibrated meta-learners, Bayesian model averaging, or constrained weight optimization), expand the diversity and number of base learners (including graph neural networks and calibrated gradient-boosting variants), and incorporate uncertainty quantification (conformal prediction) to better characterize model confidence. Additional avenues include semi-supervised or positive–unlabeled learning to leverage unlabeled chemical space, scaffold- and temporal-split evaluations for stronger generalization checks, and prospective external validation on newly added *smProdrugs* entries. Finally, integrating mechanism-aware features (e.g., bioreversible trigger motifs, enzyme/tissue activation priors, and PK/ADMET descriptors) with the current fingerprint views may further improve both performance and chemical interpretability.

In summary, as the first openly available machine-learning framework purpose-built for prodrug screening, *Prodrug-ML* necessarily carries several limitations—most notably its reliance on fingerprint-level representations, binary labels, and a still-modest corpus of curated positives—yet these constraints should be viewed as a springboard rather than a ceiling. The present study establishes a transparent, bias-aware baseline that resolves the “no true negatives” challenge, stabilizes evaluation via late-fusion over view-specific selection, and translates predictions into actionable design cues through complementary analyses (PCA/t-SNE, SHAP, ANOVA). Looking forward, the roadmap we outline—mechanism-aware labels and descriptors, species/tissue priors, applicability-domain quantification and conformal risk control, richer ensembling, and prospective testing as new prodrugs are curated—provides concrete avenues to evolve *Prodrug-ML* from early triage toward calibrated, mechanistically annotated prioritization.

## Conclusion

Reliable *in silico* Prodrug screening remains constrained by missing experimentally verified negatives, the absence of mechanism- and rate-aware models with species resolution, limited treatment of applicability domain and uncertainty, and the lack of a standardized, calibrated score for early triage at library scale. Prodrug-ML is positioned as an upstream, machine learning-based screening framework to bridge these gaps in practical settings—prioritizing enumerated promoiety/attachment-site variants in lead optimization, enriching virtual libraries before docking and ADMET assays, and retrospectively ranking legacy collections to surface overlooked candidates, with operating points chosen via validation-guided thresholds or top-*k* ranking. The framework contributes to prodrug research in four ways: (i) it offers a reproducible solution to the long-standing “no-negatives” bottleneck through three orthogonal decoy cohorts coupled with cross-decoy validation; (ii) it delivers a calibrated prodrug-likeness score and clear operating guidance that enable list-size control and focused allocation of synthesis/ADMET resources; (iii) it provides component-level diagnostics—feature-set curation, correlation structure, dimensionality reductions, and cross-recipe transfer—that illuminate prodrug-relevant patterns and support mechanism-aware extensions, alongside advantages such as bias-aware dataset construction, early isolated testing, a reproducible pipeline, and deployable threshold guidance; and (iv) as one of the first openly documented, structure-based machine-learning frameworks, it establishes a transparent reference point to catalyze future, mechanism-informed developments despite acknowledged limitations.

Prodrug-ML mitigates the long-standing “no-true negatives” problem by assembling three complementary decoy sources: (i) *DUD-E–style near-misses* that match global properties yet lack prodrug-defining motifs, (ii) *random ChEMBL* molecules providing a broad chemical backdrop, and (iii) *strictly filtered ChEMBL* entries that satisfy drug-like priors while excluding plausible activation handles. This triad curbs property-mismatch shortcuts and yields more faithful early-ranking tests. Specifically, ensembles reach $\textrm{EF}@1\%\approx 6\text {--}8$ and $\textrm{EF}@5\%\approx 5\text {--}6$, with strong ranking concentration reflected by $\textrm{BEDROC}_{20}\approx 0.78\text {--}0.82$, $\textrm{BEDROC}_{50}\approx 0.90\text {--}0.95$, and $\textrm{BEDROC}_{80}\approx 0.95\text {--}0.99$. These findings—particularly the high BEDROC values—indicate that at least a prodrug is concentrated within the top $\sim 2\text {--}3\%$ of the ranking, enabling standard wet-lab workflows to focus on the early tranche and thereby reduce immediate experimental time and cost by $\sim 95\text {--}98\%$. Also, single-view baselines deliver acceptable discrimination (e.g., ROC AUC in the mid-0.8’s, AP around 0.60), but the proposed multimodel ensemble feature selection, late-fusion approach consistently improves both *early enrichment* and *global* metrics. Concomitantly, ROC AUC ($\sim 0.86\text {--}0.87$), AP ($\sim 0.60\text {--}0.65$), and F1 ($\sim 0.58\text {--}0.62$) are stabilized across folds, supporting robust screening utility.

Taken together, the domain-bias audit and cross-decoy validation demonstrate that Prodrug-ML’s performance is not driven by dataset artifacts, recipe shortcuts, or hidden leakage. Single-view feature sets (*List_1*, *List_2*) operate near chance in origin-prediction tests, confirming minimal domain bias, while naïve early fusion and the full 2048-dimensional fingerprint set reveal spurious origin signals that justify the need for careful feature selection. Cross-decoy transfer further shows that *List_2* generalizes robustly across alternative negative constructions, preserving rank quality and precision–recall balance under recipe shift. These results collectively validate that Prodrug-ML learns prodrug-relevant structure rather than decoy-specific cues, ensuring the credibility of its reported screening performance.

The multi-pronged diagnostic analyses—label randomization, PCA, t-SNE, ANOVA F-tests, correlation heatmaps, hierarchical clustering, and RadViz—clarify why Prodrug-ML achieves consistent performance gains. They show that the predictive signal is subtle, heavy-tailed, and distributed across weakly correlated feature subsets, with no single feature or linear projection dominating. This structure necessitates view-specific feature selection, late-fusion ensembling, and non-linear learners to aggregate diffuse cues while mitigating redundancy and domain artifacts. Accordingly, Prodrug-ML’s improvement stems not from brute-force feature aggregation but from a principled design that leverages modular structure, amplifies faint signals, and preserves generalizability across decoy recipes. This combination explains its stable enrichment factors, elevated BEDROC scores, and robust transfer to independent test data, reinforcing its practical utility in prodrug-likeness prediction.

## Supplementary information

In the Supplementary Information, additional background details pertinent to the study are provided to offer a deeper context and support the primary manuscript. Furthermore, supplementary experiments and comparative results are included to validate and enhance the findings presented in the main text. These supplementary materials aim to provide comprehensive insights and strengthen the overall conclusions of the research.

### Extended feature analyses of Prodrug-ML

To complement the main-text validation (domain-bias audits, cross-decoy transfer, EF/BEDROC profiles), this section gathers concise, reader-oriented diagnostics that illuminate how selected features co-vary, cluster, and distribute relative to the model’s decision function. The goal is interpretability and quality control: (i) to detect redundancy or unintended collinearity that may affect stability, (ii) to reveal higher-order groupings of descriptors that the model might implicitly exploit, and (iii) to provide an intuitive, low-dimensional view of class/score separation without claiming linear separability. The following brief summaries guide navigation; full figures and captions are provided in the corresponding subsections.

- Correlation heatmap presents pairwise (absolute) correlation among the retained features, highlighting blocks of redundancy and potential collinearity. This aids in judging whether feature selection produced a compact, non-degenerate set and in identifying descriptor groups that move together across the dataset.
- Hierarchical feature clustering (dendrogram) Applies agglomerative clustering to the correlation structure to expose multi-scale feature groupings. Branch lengths reflect inter-feature dissimilarity, helping to spot families of descriptors that may represent similar chemical signals or complementary subspaces.
- Multivariate feature visualization with radviz Provides an interpretable, radial projection of multivariate data where each feature (anchor) exerts a pull on points proportional to its value. This view facilitates a qualitative check of score/class dispersion across anchors, offering intuition about which feature sets jointly influence the prodrug-likeness landscape.

#### Correlation heatmap

**Fig. 14:**
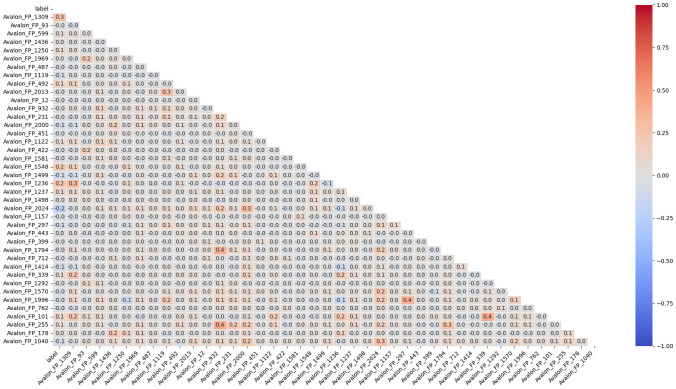
Pairwise Pearson correlations among the top *List_1* fingerprint bits (and with the *label*), shown as a down-triangular heatmap; darker red/blue indicate stronger positive/negative correlation, respectively. Axes list the selected *List_1* features (Avalon bits) plus the binary *label*; each cell shows the Pearson correlation in $[-1,1]$ and its numeric value. The color bar to the right encodes magnitude and sign

The *List_1* correlation heatmap (Fig. 14) indicates that the class signal is diffuse and that the retained bits are only weakly redundant. With respect to the label, the strongest single association is Avalon_FP_1309 at about $-0.30$, while most other bit–label correlations lie in a narrow $\pm 0.10{-}0.20$ band and many are near 0.00; this matches the ANOVA finding that no single bit explains the task and that discrimination emerges by aggregating many weak cues. Inter-feature correlations are similarly modest: the bulk of pairwise entries fall below $\sim 0.15$, with a few localized pockets reaching $\sim 0.30{-}0.40$ (e.g., around combinations such as FP_1794–FP_712). Such a profile implies limited multicollinearity—useful diversity with only mild clusters—so models trained on *List_1* can combine complementary bits without being dominated by a few highly correlated features. Practically, this supports the view-specific selection and late-fusion design in Prodrug-ML: by emphasizing the small set of informative bits and averaging over largely uncorrelated signals, the system achieves robust early enrichment while keeping overfitting risk and domain-specific artifacts in check.

**Fig. 15:**
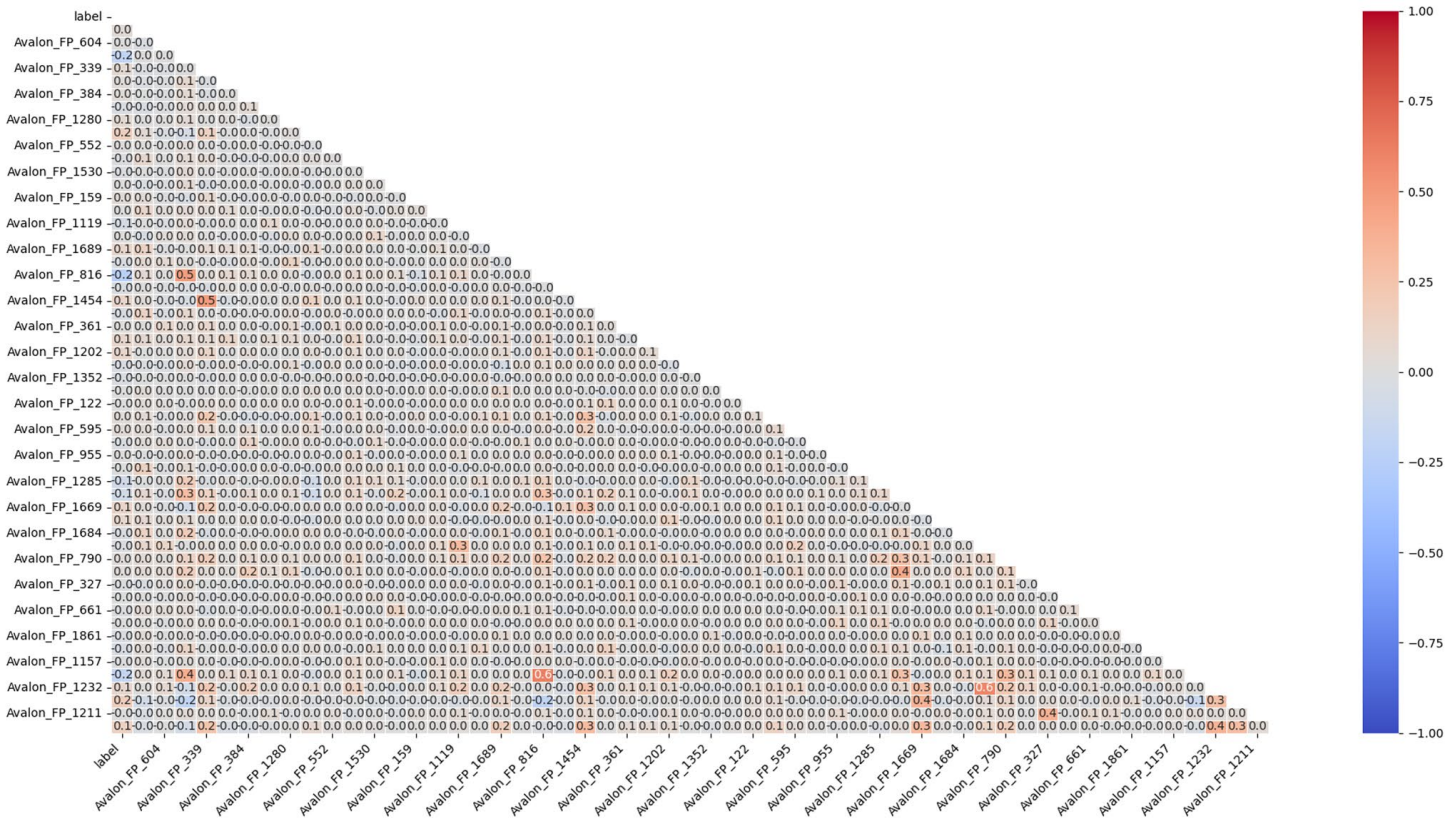
Pairwise Pearson correlations among the selected *List_2* fingerprint bits (and the *label*), shown as an upper-triangular heatmap; darker red/blue indicates stronger positive/negative correlation, respectively. Axes enumerate the retained *List_2* (Avalon) bits plus the binary *label*; each cell displays the Pearson correlation in $[-1,1]$ together with its numeric value

The *List_2* correlation map (Fig. 15) shows that class information is diffuse and that the selected bits are largely complementary rather than redundant. Bit–label associations are uniformly small: the strongest single effect is Avalon_FP_816 at roughly $r\!\approx \!-0.22$, while most features lie in a narrow $|r|\!\le \!0.10{-}0.15$ band and many are near 0. Pairwise feature–feature correlations are similarly modest—dominantly $r<0.15$ with only scattered micro-clusters reaching $\sim 0.25{-}0.35$—indicating limited multicollinearity across the retained descriptors. This pattern aligns with the ANOVA profile (several mid-strength contributors, long tail) and helps explain the stronger cross-decoy transfer observed for *List_2*: the model aggregates many weak, partly independent signals rather than relying on a few tightly correlated cues that could encode recipe-specific artifacts. Practically, it supports the view-specific selection and late-fusion design in Prodrug-ML, where these weakly correlated bits can be combined to yield robust early enrichment without inflating domain bias.

In summary, across both views, the correlation heatmaps reveal a diffuse class signal with uniformly small bit–label associations and low inter-feature correlations. In *List_1*, only a few bits reach moderate correlation with the label (e.g., $\sim \!-0.30$), and feature–feature correlations are mostly $<0.15$ with only mild local pockets; *List_2* is even more diffuse (peak $\sim \!-0.22$) and similarly low in multicollinearity. Such a pattern indicates that predictive power arises from combining many weak, largely complementary cues rather than from a handful of highly redundant descriptors. Consequently, the view-specific selection plus late-fusion design of Prodrug-ML is well matched to the data structure, helping curb overfitting, avoid recipe-specific artifacts, and support the robust early enrichment observed elsewhere.

#### Hierarchical feature clustering (dendrogram)

**Fig. 16:**
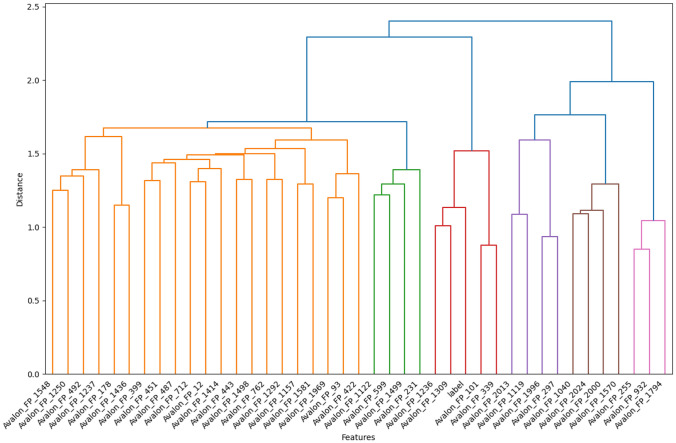
Hierarchical clustering (dendrogram) of the selected *List_1* fingerprint bits (plus the binary label), showing how features group by similarity and at what linkage distance they merge. The dendrogram arranges features as leaves on the x–axis and joins them bottom–up according to their pairwise similarity (y–axis: linkage “Distance”). Short vertical joins indicate closely related bits; long joins mark weak relationships

Figure 16 shows that interpreting the dendrogram at a color threshold of $\sim 1.7$ (the height where branch colors change) yields six coarse groups (orange, green, red, purple, brown, and pink). Most within-group merges occur at low heights ($<1.0$–1.2), whereas groups fuse with each other only at much larger distances ($\sim 1.7$–2.4); the final joins above $\sim 2.3$ indicate weak global redundancy and fairly distinct modules. The *label* lies inside a compact red micro-cluster and merges first with the most label-informative bit from earlier analyses, Avalon_FP_1309, at a short distance ($\lesssim 1.0$), then with a few mid-strength neighbors (e.g., Avalon_FP_101, Avalon_FP_231/236) before joining the rest of the tree. Outside this label-proximal module, one large orange super-cluster collects many loosely related bits (shallow branch heights), while several smaller modules (green/purple/brown/pink) sit farther away and merge only at higher distances, suggesting they encode complementary substructures (Fig. 16). Overall, the tree supports three conclusions: (i) limited multicollinearity within clusters, (ii) clear separation between clusters, and (iii) a small, label-adjacent feature set anchored by FP_1309. These properties justify selecting a few representatives per cluster and align with Prodrug-ML’s view-specific selection and late-fusion strategy to combine signals from distinct, weakly overlapping feature groups.

**Fig. 17:**
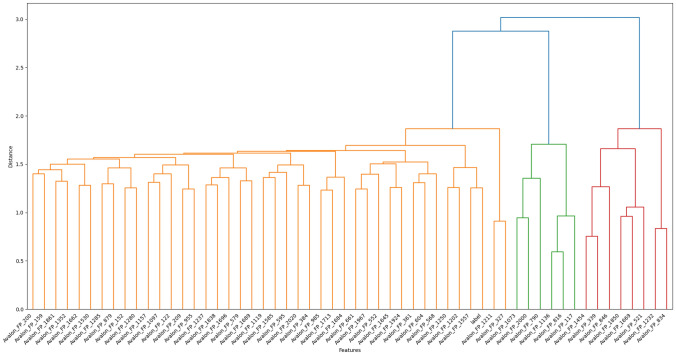
Hierarchical-clustering dendrogram of *List_2* features. Branch colors indicate clusters under a color threshold of $\sim 1.7$. The *x*-axis lists features (including the target label), and the *y*-axis shows linkage distance. Agglomerative dendrogram in which each leaf on the *x*-axis is a feature name (Avalon_FP indices and the label), and vertical position reflects the linkage distance at which merges occur. Colored branches denote clusters defined by a color threshold of $\approx 1.7$. A broad orange cluster occupies the left portion, a compact green cluster appears near the center, and a red cluster is visible on the right; higher, uncolored (blue) branches connect these colored groups at larger distances. Horizontal segments mark merge events, with their heights indicating the distance of the corresponding joins. Axis labels “Features” (*x*) and “Distance” (*y*) specify the layout, and ticked leaf labels identify individual variables

As for the hierarchical-clustering dendrogram for list_2, Fig. 17, using a color threshold of $\sim 1.7$, the tree resolves three clusters: a large orange block occupying most features (left), a compact green block of six features Avalon_FP_2000, Avalon_FP_790, Avalon_FP_1136, Avalon_FP_816, Avalon_FP_117, Avalon_FP_1454 near the center-right, and a red block of seven features Avalon_FP_339, Avalon_FP_846, Avalon_FP_1850, Avalon_FP_1669, Avalon_FP_521, Avalon_FP_1232, Avalon_FP_834 at the far right. Within-cluster merges generally occur at lower heights (orange $\approx 1.2\text {--}1.6$; green $\lesssim 1.4$; red $\lesssim 1.8$), while between-cluster joins happen later: the green and red blocks come together around $\sim 1.7\text {--}1.9$, and the combined right-hand block fuses with the large orange block only at $\sim 2.9\text {--}3.0$. The *label* is located inside the orange block, close to the orange/green boundary Fig. 17. It first pairs tightly with Avalon_FP_1211 (short distance, $\lesssim 1.0$) and then links into a small orange submodule that includes nearby leaves such as Avalon_FP_327 and Avalon_FP_1073; additional neighboring orange features in this vicinity include Avalon_FP_1557, Avalon_FP_1202, Avalon_FP_1250, Avalon_FP_568, and Avalon_FP_604.

Taken together, the hierarchical-clustering analyses of *List_1* and *List_2* demonstrate that the fingerprint features organize into a limited number of well-defined modules, with strong within-cluster cohesion and clear separation across clusters. Importantly, both trees position the *label* within compact, low-distance neighborhoods anchored by highly informative features, while broader groups merge only at larger linkage heights, indicating complementary rather than redundant signal. These properties justify representative sampling within clusters and support the strategy of view-specific feature selection combined with late fusion. In this way, Prodrug-ML exploits the modular structure of the feature space while minimizing collinearity, ensuring that retained signals are complementary, weakly overlapping, and maximally informative for prodrug-likeness prediction.

#### Multivariate feature visualization with RadViz

**Fig. 18:**
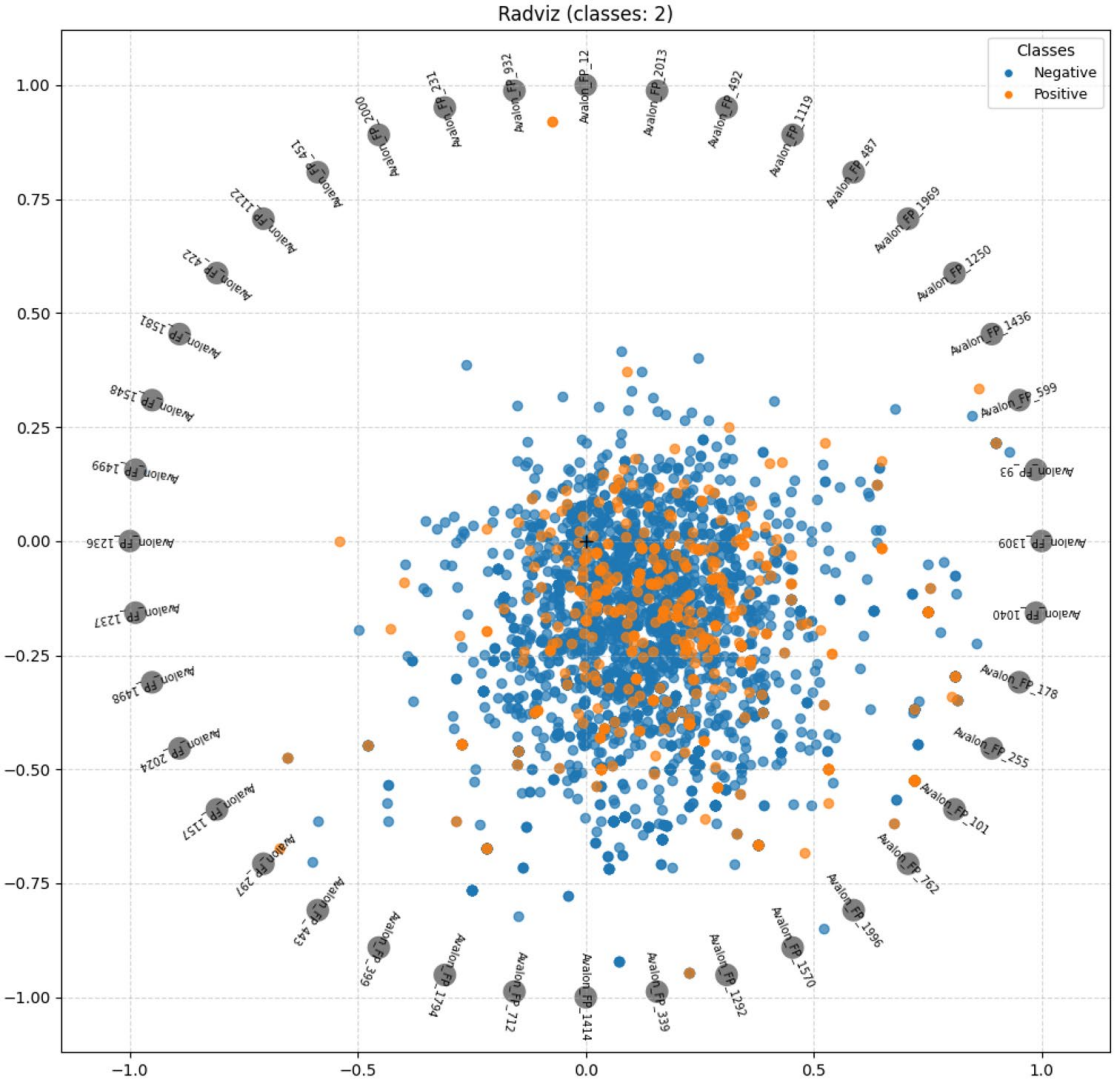
RadViz projection of the list_1 feature space: each sample is mapped inside a unit circle and attracted by Avalon fingerprint anchors placed on the perimeter, with class membership indicated by the legend. The figure provides a 2-D, model-agnostic visualization of the multivariate data used in *list_1*. Gray circular markers on the circle’s boundary denote feature anchors (e.g., *Avalon_FP_xxx*), and each point inside the circle represents one sample positioned by its normalized values relative to these anchors. Axes and gridlines show the $[-1,1]$ plotting range, while the legend encodes the two classes with distinct colors. This view documents the anchor layout, overall point dispersion, and presence of any sparsely populated or outlying regions in the *list_1* space

The RadViz projection for *List_1* (Fig. 18) shows a dense, nearly isotropic cloud of samples concentrated near the disk center, reflecting the comparable magnitudes of normalized features and the absence of any dominant anchor. Positive and negative classes largely overlap, indicating that linear separability is weak and that any discriminative signal is likely dispersed across many features and only recoverable by non-linear learners or higher-order interactions. Feature anchors are distributed along the circumference, with several positioned in close angular proximity, suggesting redundancy or correlation among those bits; most samples remain centrally attracted, consistent with the sparse, binary nature of Avalon fingerprints. A small number of rim points reflect atypical feature profiles that may correspond to unusual chemotypes, batch effects, or preprocessing heterogeneity. Overall, this visualization underscores that class information is subtle and distributed, supporting the need for Prodrug-ML’s multimodel, feature-selection, and ensembling strategy to amplify weak signals and achieve reliable screening performance.

**Fig. 19:**
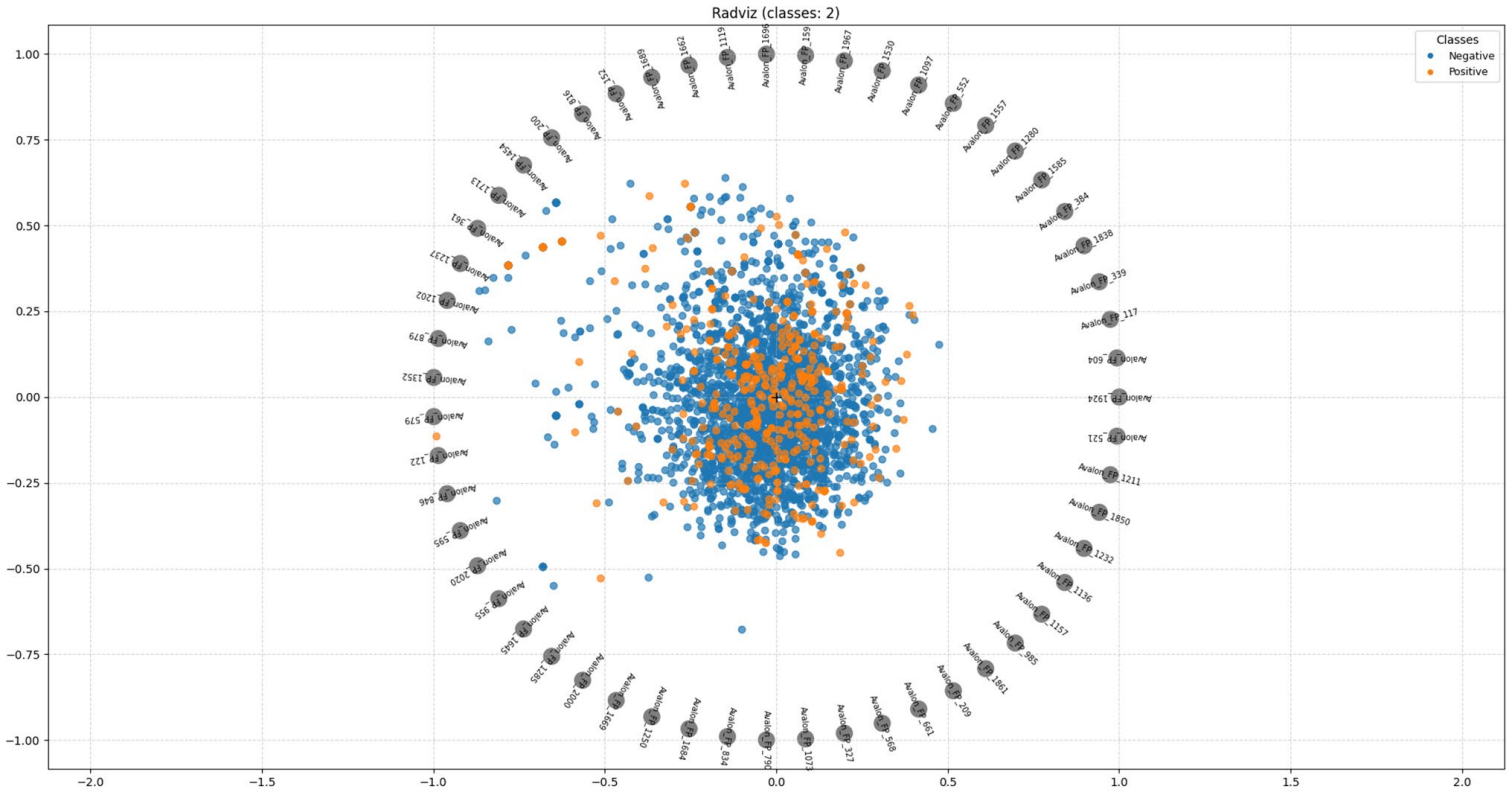
RadViz projection of the List_2 feature space for a binary dataset, with features placed as anchors on the unit circle and samples colored by class. The figure is a RadViz scatter plot titled “RadViz (classes: 2).” Gray circular nodes arranged around the perimeter are the List_2 feature anchors, each labeled with its Avalon_FP index (e.g., Avalon_FP_1211, Avalon_FP_521, Avalon_FP_604, Avalon_FP_117, Avalon_FP_339, ...). Interior points represent individual samples, colored according to the legend at the upper right (*Negative* in blue, *Positive* in orange). Coordinates arise from convex combinations of normalized feature values pulled toward their corresponding anchors; axis ticks span approximately $[-2,2]$ on the x-axis and $[-1,1]$ on the y-axis, with grid lines shown. Most points lie within the central region of the circle, while the rotated labels around the circumference indicate the positions of all anchors used to construct the projection

The RadViz projection for *List_2* (Fig. 19) reveals a dense central core where positive and negative samples substantially overlap, surrounded by a sparse ring of points drawn toward individual feature anchors. This pattern reflects the absence of any single dominant anchor and indicates that discriminative information is distributed across combinations of features rather than concentrated in univariate effects. Several anchors appear in tight angular clusters, suggesting redundancy, while more isolated anchors contribute distinctive pulls that explain the few peripheral points near the rim—likely corresponding to atypical chemotypes or substructure-specific patterns. The overall cloud exhibits mild elongations toward anchor-dense sectors, consistent with correlated feature groups, but no class-pure lobes emerge. Taken together, the geometry underscores that effective modeling with *List_2* requires multivariate interaction capture, careful handling of semi-redundant subsets, and robust learners capable of amplifying weak, distributed signals—an approach aligned with Prodrug-ML’s late-fusion and ensemble feature-selection strategy.

In summary, the RadViz visualizations of *List_1* and *List_2* converge on a common picture: class information is subtle, highly overlapping, and distributed across many weakly discriminative features rather than concentrated in a few dominant dimensions. Both projections highlight redundancy among tightly clustered anchors as well as the presence of occasional outlier points drawn toward isolated features, reflecting the sparse and correlated nature of Avalon fingerprints. The lack of clear class-pure lobes underscores the necessity of employing non-linear models and ensemble-based strategies that can capture multivariate interactions. These observations justify the design of Prodrug-ML around view-specific feature selection and late-fusion ensembling, which together amplify diffuse signals, mitigate redundancy, and deliver robust predictive performance in the face of weakly stratified input spaces.

### Selected feature sets

Two single-view Avalon fingerprint feature sets—List_1 and List_2—were selected through a domain-bias–aware screen and confirmed under cross-decoy validation. To minimize recipe-specific leakage, the views are kept separate and combined only at the score (late-fusion) stage; naïve early fusion is avoided because it can increase origin predictability without a commensurate task signal.

Full contents:

list_1 = [Avalon_FP_1309, Avalon_FP_93, Avalon_FP_599, Avalon_FP_1436, Avalon_FP_1250, Avalon_FP_1969, Avalon_FP_487, Avalon_FP_1119, Avalon_FP_492, Avalon_FP_2013, Avalon_FP_12, Avalon_FP_932, Avalon_FP_231, Avalon_FP_2000, Avalon_FP_451, Avalon_FP_1122, Avalon_FP_422, Avalon_FP_1581, Avalon_FP_1548, Avalon_FP_1499, Avalon_FP_1236, Avalon_FP_1237, Avalon_FP_1498, Avalon_FP_2024, Avalon_FP_1157, Avalon_FP_297, Avalon_FP_443, Avalon_FP_399, Avalon_FP_1794, Avalon_FP_712, Avalon_FP_1414, Avalon_FP_339, Avalon_FP_1292, Avalon_FP_1570, Avalon_FP_1996, Avalon_FP_762, Avalon_FP_101, Avalon_FP_255, Avalon_FP_178, Avalon_FP_1040]

list_2 = [Avalon_FP_1924, Avalon_FP_604, Avalon_FP_117, Avalon_FP_339, Avalon_FP_1838, Avalon_FP_384, Avalon_FP_1585, Avalon_FP_1280, Avalon_FP_1557, Avalon_FP_552, Avalon_FP_1097, Avalon_FP_1530, Avalon_FP_1967, Avalon_FP_159, Avalon_FP_1696, Avalon_FP_1119, Avalon_FP_1662, Avalon_FP_1689, Avalon_FP_152, Avalon_FP_816, Avalon_FP_200, Avalon_FP_1454, Avalon_FP_1713, Avalon_FP_361, Avalon_FP_1237, Avalon_FP_1202, Avalon_FP_879, Avalon_FP_1352, Avalon_FP_579, Avalon_FP_122, Avalon_FP_846, Avalon_FP_595, Avalon_FP_2020, Avalon_FP_955, Avalon_FP_1645, Avalon_FP_1285, Avalon_FP_2000, Avalon_FP_1669, Avalon_FP_1250, Avalon_FP_1684, Avalon_FP_834, Avalon_FP_790, Avalon_FP_1073, Avalon_FP_327, Avalon_FP_568, Avalon_FP_661, Avalon_FP_209, Avalon_FP_1861, Avalon_FP_985, Avalon_FP_1157, Avalon_FP_1136, Avalon_FP_1232, Avalon_FP_1850, Avalon_FP_1211, Avalon_FP_521]

Overlap and complementarity: Let $A=$list_1 and $B=$list_2. Cardinalities are $|A|=40$, $|B|=55$, and $|A\cup B|=89$ unique bits. The intersection contains 6 shared bits:

$$\begin{aligned} A\cap B&=\{\texttt {Avalon\_FP\_1119}, \\&\texttt {Avalon\_FP\_1157}, \\&\texttt {Avalon\_FP\_1237}, \texttt {Avalon\_FP\_1250},\\&\quad \texttt {Avalon\_FP\_2000}, \texttt {Avalon\_FP\_339}\}. \end{aligned}$$

Overlap fractions are $6/40=15.0\%$ of list_1 and $6/55\approx 10.9\%$ of list_2. Set-similarity indices indicate strong complementarity:

$$\begin{aligned} \text {Jaccard}=\frac{|A\cap B|}{|A\cup B|}=\frac{6}{89}\approx 0.067,\qquad \text {Dice}=\frac{2|A\cap B|}{|A|+|B|}=\frac{12}{95}\approx 0.126. \end{aligned}$$

The small but non-trivial intersection suggests stable substructures repeatedly selected under independent screens, while the large unique portions (34/40 in list_1, 49/55 in list_2) provide complementary coverage that improves early-recognition and BEDROC without amplifying domain-recipe cues. Maintaining the two views as separate inputs and fusing at the score level preserves diversity benefits while limiting origin predictability.

Each Avalon_FP_# index corresponds to a hashed substructure key from a fixed-length Avalon fingerprint (RDKit/pyAvalonTools). These indices do not map one-to-one to human-readable fragments; nevertheless, recurrence across views (e.g., 1119, 1157, 1237, 1250, 2000, 339) is consistent with chemically meaningful motifs that persist across alternative decoy constructions.

### Other model performances

**Fig. 20:**
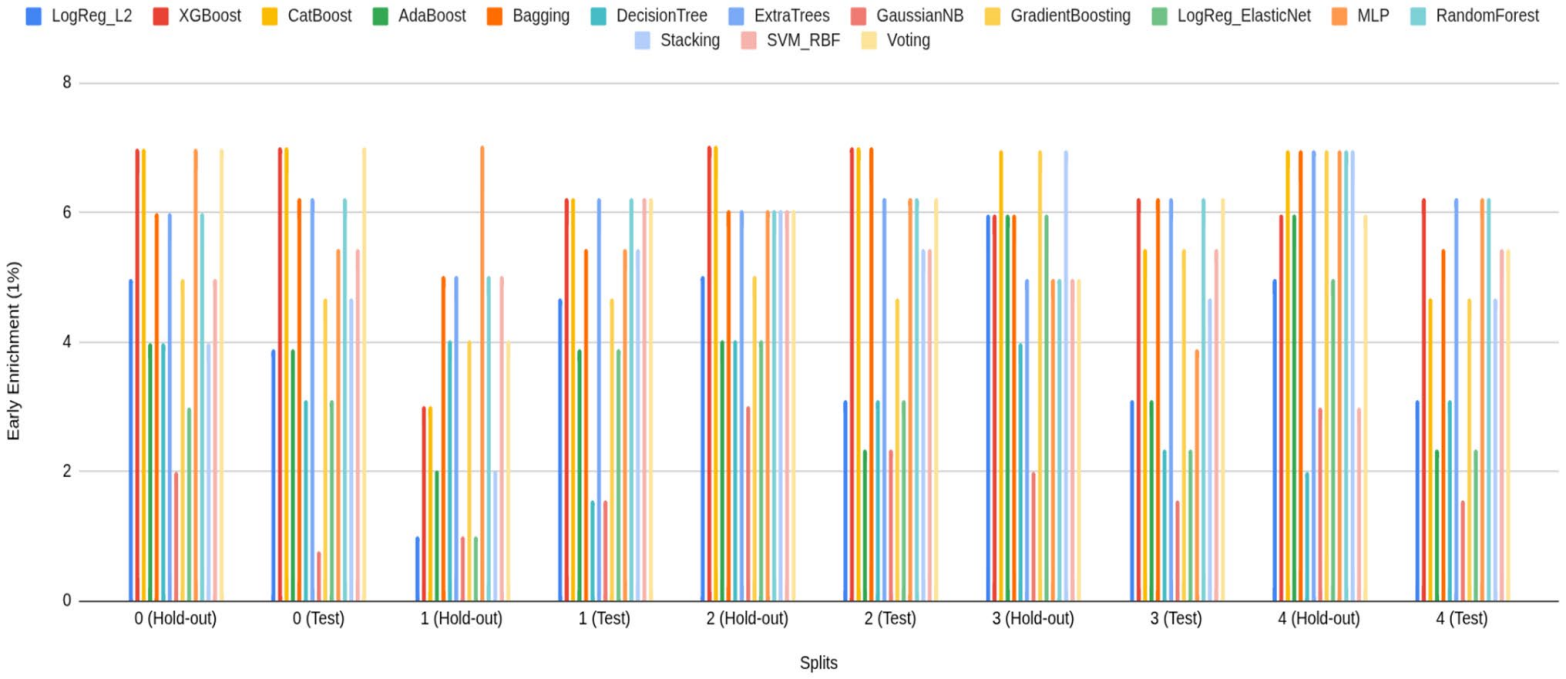
The results of Early Enrichment at 1% for multiple classifier families across five cross-validation splits, shown for both hold-out and test partitions. Bar chart of EF@1% (Early Enrichment, y-axis) for 15 models (legend) evaluated across five splits (0–4), with each split shown twice on the x-axis—once for Hold-out and once for Test—to display validation and external-set performance per model

In order to inhibit model selection bias, several models have been used and also tested using cross-validation. Across splits, ensemble and boosted trees dominate the extreme top of the ranked lists: XGBoost, CatBoost, ExtraTrees, Bagging, and Voting frequently attain $\textrm{EF}@1\% \approx 6\text {-}7$, with multiple peaks near 7 on splits $0\text {-}2$ for both Hold-out and Test, indicating highly concentrated early retrieval (Fig. 20). A second tier—LogReg_L2, RandomForest, Stacking, GradientBoosting, and MLP—typically yields $\textrm{EF}@1\% \approx 5\text {-}6$. Values above 5 are operationally meaningful in prospective screening, as they imply substantially more actives surfaced within the top $1\%$ than expected by chance. Beyond the pre-selected models (e.g., k-NN, LightGBM (default classifier in Prodrug-ML), and Bagging), additional families visible in the panel—such as RandomForest, Stacking, and GradientBoosting—also exhibit $\textrm{EF}@1\%>5$ on multiple splits, reinforcing that the observed gains are not idiosyncratic to a single algorithmic choice (Fig. 20). The Hold-out $\Rightarrow$ Test generalization gap for leading methods is modest (paired bars typically within $\sim 0.5\text {-}1$ EF point across splits), suggesting robustness to resampling and background shift. Taken together, the figure supports the conclusion that tree-based ensembles set the upper envelope for top-$1\%$ recovery, while linear, kernel, and simple tree baselines show lower ceilings and greater variance, yet several diverse learners still surpass the $\textrm{EF}@1\%>5$ significance threshold across folds.

**Fig. 21:**
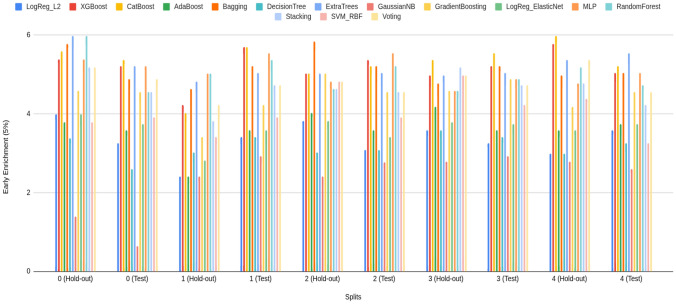
Early-enrichment at 5% for 15 learning algorithms across five splits, shown for both Hold-out and Test. The figure is a grouped bar chart comparing *15 models* (legend) on *Early Enrichment at 5%* (y-axis; roughly spanning 2–6). The *x-axis* lists *five splits* (0–4), each shown twice—“(Hold-out)” and “(Test)”—yielding *ten bar groups* in total. Each group contains one bar per model, enabling side-by-side comparison of generalization from Hold-out to Test. Tree-based ensembles (XGBoost, CatBoost, Bagging, RandomForest, ExtraTrees) form the upper band (often $\gtrsim 5$), whereas simpler baselines (e.g., GaussianNB, single DecisionTree) tend to sit lower. The close heights of paired Hold-out$\rightarrow$Test bars within each split suggest *modest* generalization gaps

Figure 21 demonstrates that tree-based ensembles again set the pace at early retrieval: XGBoost, CatBoost, ExtraTrees, Bagging, and Voting populate the upper envelope of the panel with $\textrm{EF}@5\%$ values clustering around 5–6 and multiple peaks near the top of the axis, indicating concentrated recovery well above the random baseline ($\textrm{EF}=1$). A second tier—LogReg_L2, RandomForest, Stacking, GradientBoosting, and MLP—typically occupies the 4–5 band, still materially exceeding chance and thus practically useful for prioritization. Beyond the pre-selected leaders, additional families visible in the panel (e.g., RandomForest, Stacking, GradientBoosting) also achieve $\textrm{EF}@5\% \gtrsim 4$ on multiple splits, reinforcing that the gains are not idiosyncratic to one algorithmic choice. Hold-out $\Rightarrow$ Test generalization gaps for the top methods are modest—paired bars are usually within roughly one EF point—suggesting robustness to resampling and background shift. Taken together, the figure supports the conclusion that ensemble trees provide the strongest and most stable top-$5\%$ enrichment, while linear, kernel, and single-tree baselines show lower ceilings and greater variance, yet several diverse learners still deliver practically meaningful enrichment across folds.

**Fig. 22:**
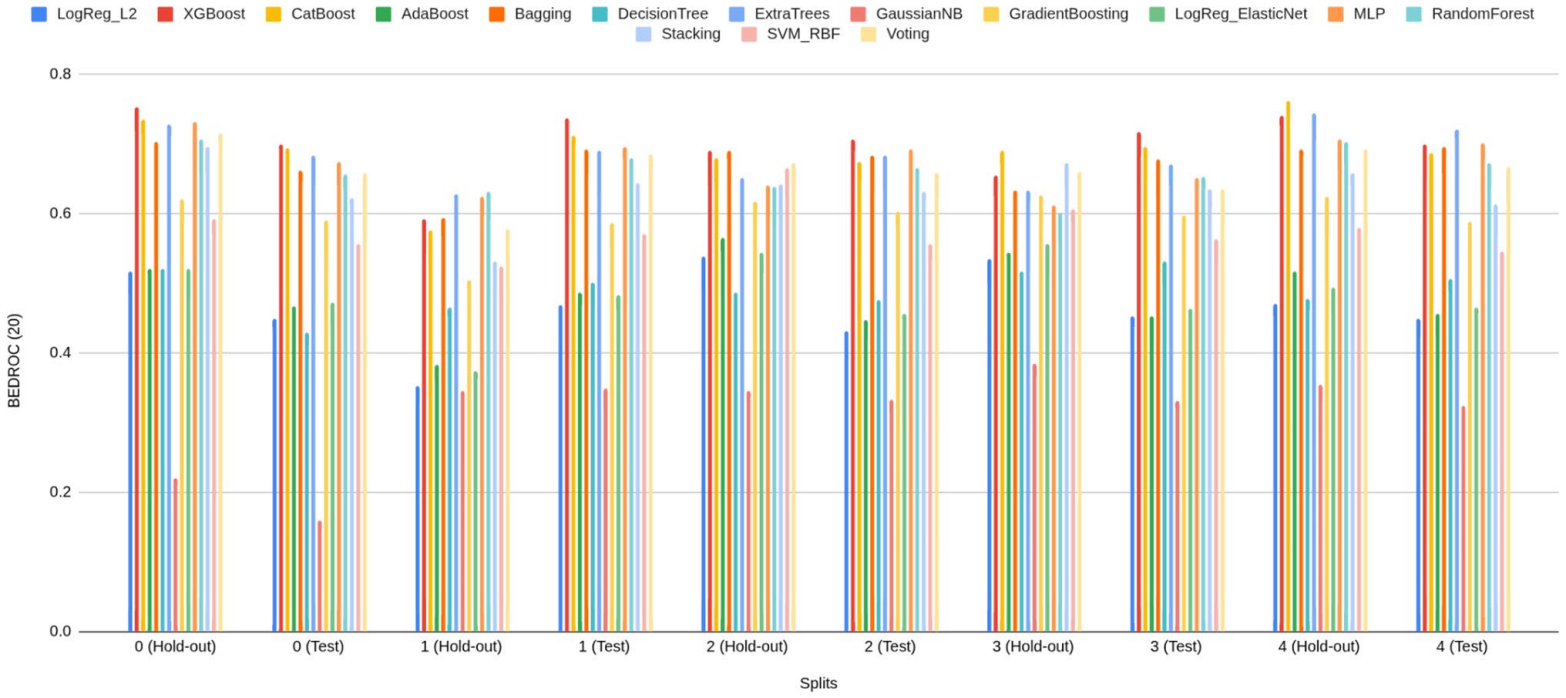
The results of $\textit{BEDROC}_{20}$ for 15 models (legend) on hold-out data and early split test set. The figure is a grouped bar plot comparing *15 models* (legend) on $\textit{BEDROC}_{20}$; the *y-axis* reports $\text {BEDROC}_{20}$ values (higher is better for early-ranking), spanning roughly 0.3-$-$0.75. The *x-axis* lists *five splits* (0–4), each shown twice—“(Hold-out)” and “(Test)”—yielding *10 groups* in total; within each group, there is one bar per model to enable side-by-side generalization checks

The Fig. 22 demonstrates that early-recognition, as quantified by $\textrm{BEDROC}_{20}$, is consistently strongest for tree-based ensembles—XGBoost, CatBoost, Bagging, RandomForest, and ExtraTrees—which form the upper envelope across all splits and both evaluation regimes (Hold-out and Test); for example, on split 0 XGBoost sits near the top on Hold-out and remains only slightly lower on Test, with CatBoost and RandomForest closely trailing, a pattern that recurs on splits 1–4 and indicates concentrated recovery well above chance. A second tier—LogReg_L2, GradientBoosting, Stacking, and MLP—occupies a mid-to-high band, often just beneath the ensembles and thus promising for diversification in downstream ensembling, whereas simpler baselines such as a single DecisionTree or GaussianNB typically inhabit the lower portion, reflecting limited capacity to capture non-linear structure under strong early weighting. Generalization is stable for the leaders: paired Hold-out$\Rightarrow$Test bars are usually close (often within a small visible margin), preserving relative rankings and arguing against overfitting to the Hold-out distribution. Operationally, because $\textrm{BEDROC}_{20}$ heavily emphasizes the head of the ranked list, the repeated dominance of ensemble trees across splits suggests a reproducible advantage for prospective prioritization when screening budgets are scarce, while the consistent presence of competitive mid-tier models (e.g., Stacking, MLP, LogReg_L2) supports their inclusion to hedge against split-specific idiosyncrasies.

**Fig. 23:**
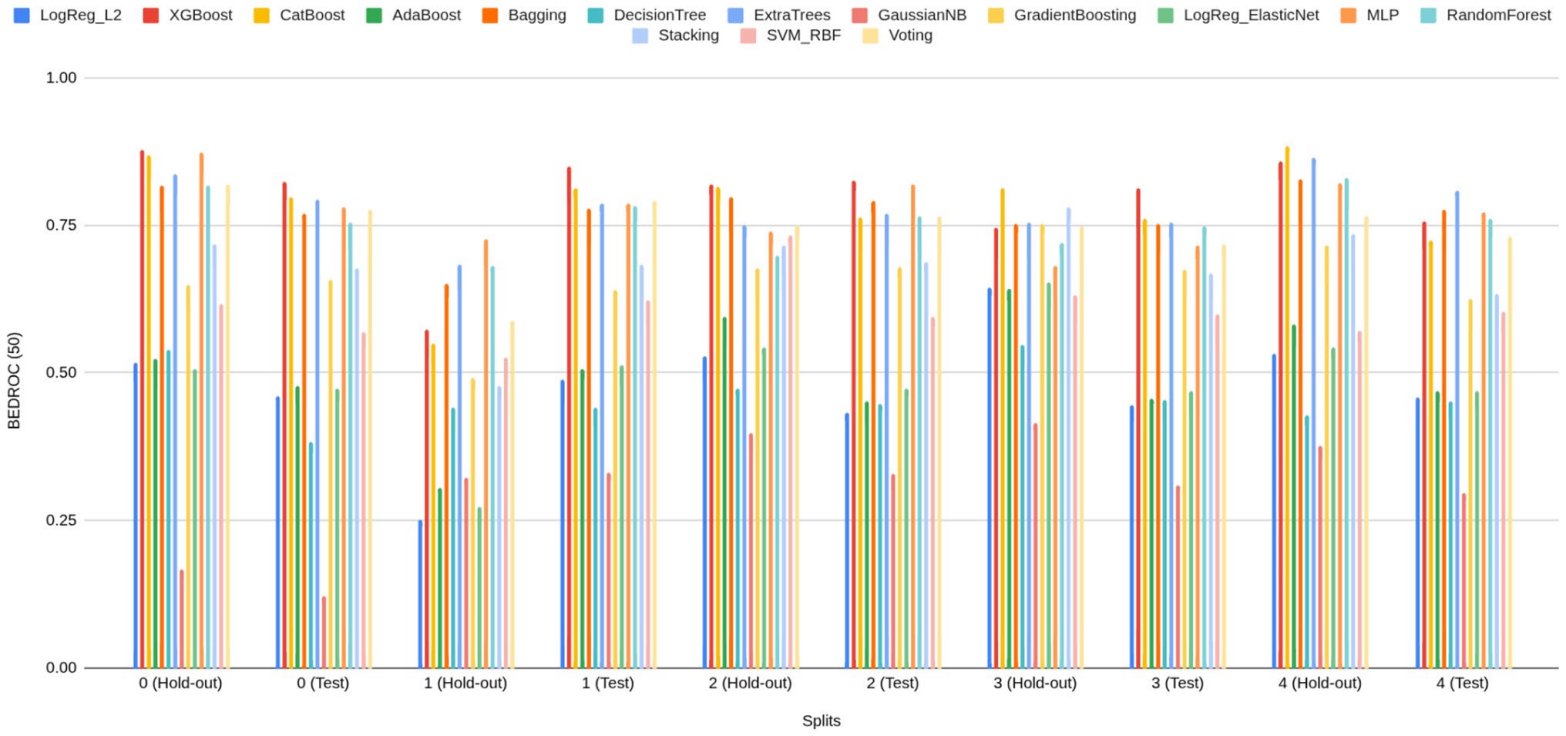
$\text {BEDROC}_{50}$ scores for 15 learning algorithms across five splits (Hold-out and Test), with tree-based ensembles (XGBoost, CatBoost, Bagging, RandomForest, ExtraTrees) consistently leading and only modest Hold-out $\rightarrow$ Test gaps. The figure is a grouped bar chart comparing *15 models* (legend) on $\textit{BEDROC}_{50}$; the *y-axis* reports $\text {BEDROC}_{50}$ (higher is better for early ranking), ranging roughly from 0.2 to 0.9, while the *x-axis* lists *five splits* (0–4), each shown twice—“(Hold-out)” and “(Test)”—for a total of *10 groups*. Each group contains one bar per model, enabling side-by-side checks of generalization from Hold-out to Test

Figure 23 indicates that the early-recognition panels (*EF@5%*, $\textit{BEDROC}_{20}$, $\textit{BEDROC}_{50}$) indicate that the pipeline yields consistently strong top-of-list recovery *without* any model cherry-picking: across all five splits and both evaluation regimes (Hold-out and Test), multiple, *diverse* learners clear practically meaningful thresholds simultaneously, and the Hold-out$\Rightarrow$Test gaps remain modest. In the EF@5% figure, tree-based ensembles—XGBoost, CatBoost, Bagging, RandomForest, ExtraTrees—repeatedly occupy the upper band (often $\gtrsim 5$), while mid-tier models such as Stacking, GradientBoosting, MLP, and LogReg_L2 track close behind, showing that gains are not idiosyncratic to a single algorithmic family. The $\textit{BEDROC}_{20}$ panel corroborates this pattern under stronger early weighting: ensemble trees again set the upper envelope with Test bars closely shadowing Hold-out bars across splits, and several non-tree baselines remain solidly above lower baselines (e.g., single DecisionTree, GaussianNB), evidencing robustness to resampling and background shift. Crucially, $\textit{BEDROC}_{50}$—which still emphasizes the head while smoothing tail effects—shows the same ordering stability, with multiple ensembles approaching the top of the axis on several splits (e.g., near $\sim 0.85$ on split 4 Hold-out) and maintaining only small declines on Test; meanwhile, mid-tier models persist in a competitive band (often $\sim 0.65$–0.8). These cross-metric, cross-split regularities imply that (i) the feature and labeling design produce a ranking signal that many algorithms can exploit, (ii) the best methods generalize with minimal drift from Hold-out to Test, and (iii) materially useful enrichment is achieved by *several* learners at once. Therefore, even without explicit model selection, the method as a whole is successful: a broad front of algorithms delivers robust early enrichment, while ensemble trees reliably set the ceiling—together supporting dependable prospective prioritization without relying on a single chosen model.

**Fig. 24:**
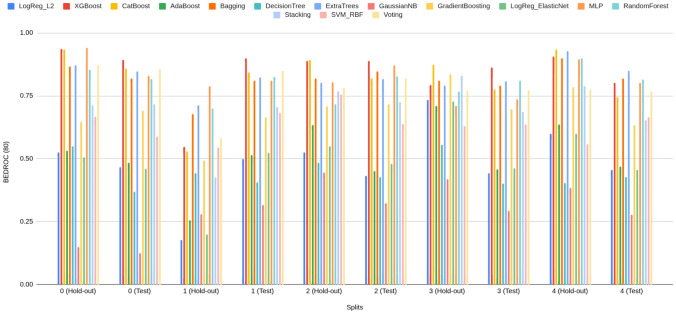
$\text {BEDROC}_{80}$ scores for 15 learning algorithms across five splits (Hold-out and Test), with tree-based ensembles (XGBoost, CatBoost, Bagging, RandomForest, ExtraTrees) consistently leading and only modest Hold-out $\rightarrow$ Test gaps. The figure is a grouped bar chart comparing *15 models* (legend) on $\textit{BEDROC}_{80}$; the *y-axis* reports $\text {BEDROC}_{80}$ (higher is better for early ranking), ranging roughly from 0.15 to 0.95, while the *x-axis* lists *five splits* (0–4), each shown twice—“(Hold-out)” and “(Test)”—for a total of *10 groups*. Each group contains one bar per model, enabling side-by-side generalization checks

Figure 24 shows that early-recognition under a very strong head-weighting consistently favors tree-based ensembles—XGBoost, CatBoost, Bagging, RandomForest, and ExtraTrees—which form the upper envelope across splits and evaluation regimes (Hold-out and Test), with several bars approaching $\sim 0.85$–0.90 (e.g., split 4 Hold-out) and only modest declines on the corresponding Test sets. A stable mid-tier comprising Stacking, GradientBoosting, MLP, and LogReg_L2 typically occupies the 0.70-$-$0.80 band, indicating practically useful prioritization even outside the very top models, while simpler baselines (single DecisionTree, GaussianNB) remain lower, reflecting limited capacity to capture non-linear structure under aggressive early weighting. Crucially, paired Hold-out$\Rightarrow$Test bars for the leading methods remain close within each split, preserving rank order and suggesting robustness to resampling and background shift. Taken together, these patterns imply that the pipeline yields reproducible top-of-list concentration without reliance on a single algorithm: ensemble trees reliably set the ceiling, yet multiple diverse learners deliver strong $\text {BEDROC}_{80}$, supporting dependable prospective prioritization without explicit model selection.

In conclusion, across five splits and both evaluation regimes (Hold-out and Test), a broad set of algorithms—spanning boosted/ensemble trees (XGBoost, CatBoost, ExtraTrees, Bagging, Voting), linear baselines (LogReg_L2, ElasticNet), kernel and neural models (SVM_RBF, MLP), and additional families (RandomForest, GradientBoosting, Stacking)—deliver consistently strong early-ranking. Top performers repeatedly reach $\textrm{EF}@1\% \approx 6\text {--}7$ with multiple peaks near 7, while $\textrm{EF}@5\%$ commonly clusters around $5\text {--}6$; simultaneously, $\textrm{BEDROC}_{20/50/80}$ occupies high bands across splits, with upper bars frequently $\gtrsim 0.75$ and examples approaching $\sim 0.85\text {--}0.90$. Paired Hold-out$\Rightarrow$Test gaps remain modest, preserving rank order and indicating robustness to resampling and background shift. Collectively, these cross-metric, cross-model, and cross-split results show that early enrichment is not confined to a single algorithmic choice; rather, many diverse learners achieve materially high $\textrm{EF}@1\%$, $\textrm{EF}@5\%$, and $\textrm{BEDROC}$ values. Therefore, the evidence validates that the evaluation is free from model-selection bias: conclusions do not hinge on any isolated model, as multiple distinct methods yield significant and reproducible early-recognition gains.

## Data Availability

Positive-class data were obtained from smProdrugs: A repository of small-molecule prodrugs. Access should be sought from the dataset’s authors. We acknowledge their support and note that we lack permission to redistribute the files. The negative datasets and codes can be found at: https://github.com/yauz3/prodrug-ml

## Code availability

Code generated during this study is available at https://github.com/yauz3/prodrug-ml from the corresponding author upon reasonable request.
